# Supplementary material for: General and Modular Strategy for Designing Potent, Selective, and Pharmacologically Compliant Inhibitors of Rhomboid Proteases
Source: Cell Chem Biol. 2017 Dec 21;24(12):1523–1536.e4. doi: 10.1016/j.chembiol.2017.09.007 (PMC5746060; doi:10.1016/j.chembiol.2017.09.007)
Supplement: Document S1. Table S1 and Methods S1 [file mmc1.pdf]

Cell Chemical Biology, Volume *24*

Supplemental Information

General and Modular Strategy for Designing Potent, Selective, and Pharmacologically Compliant Inhibitors of Rhomboid Proteases

Anežka Tichá, Stancho Stanchev, Kutti R. Vinothkumar, David C. Mikles, Petr Pachl, Jakub Began, Jan Škerle, Kateřina Švehlová, Minh T.N. Nguyen, Steven H.L. Verhelst, Darren C. Johnson, Daniel A. Bachovchin, Martin Lepšík, Pavel Majer, and Kvido Strisovsky

Supplementary Information

Methods S1

Chemical Synthesis

Fluorogenic substrates KSp35 (Ticha, et al., 2017), KSp93 (KRHRI(E-EDANS)RVRHA-DTG(K-DABCYL)IFAAISLFSLLFQPLFGLSKKR-nh2), KSp64 (KRHRI(K-TAMRA)RVRHA-DTG(C-QXL610)IFAAISLFSLLFQPLFGLSKKR-nh2) and KSp96 (KRHRINRVR(E-EDANS)A-DTG(K-DABCYL)IFAAISLFSLLFQPLFGLSKKR-nh2) were synthesised and purified as described (Ticha, et al., 2017). N-substituted peptidyl-α-ketoamides were synthesised in four stages using published methods: (1) isocyanides corresponding to the tail modification were synthesised by Hoffman isocyanide synthesis (Cao, et al., 2010), (2) the Boc-protected P1 amino acid was converted to an aminoaldehyde and then to the respective α-hydroxy-β-aminoamide by the Passerini reaction with the corresponding isocyanide (Semple, et al., 2000; Venkatraman, et al., 2006), (3) the peptide component corresponding to the P5 to P2 segment of the inhibitor was synthesised on solid phase using the 2-chlorotrityl resin, and (4) the deprotected α-hydroxy-β-aminoamide hydrochloride was coupled to the P5-P2 peptide (Coste, et al., 1994) with consequent Dess-Martin oxidation and removal of the orthogonal protection groups (Souček and Urban, 1995; Yin, et al., 2007) to yield the final N-substituted peptidyl-α-ketoamides. The unsubstituted ketoamide has been synthesized by transforming the aldehyde moiety of the Boc-protected P1 aminoaldehyde formed in step (2) to a cyanhydrin and subsequent partial hydrolysis of the cyanide moiety to an amide (Venkatraman, et al., 2006).

Peptidyl chloromethylketones were synthesised by the mixed anhydride method as described (Zoll, et al., 2014). Peptidyl acylsufonamides were synthesised by transformation of the N-protected alanine to the protected N-acylsulfonamides as described (D'Andrea and Scola, 2008) and coupling of the deprotected N-acylsulfonamide to the peptides (Coste et al., 1994). Peptidyl trifluoromethylketones were synthesized by coupling of the peptide with 3-amino-1,1,1-trifluorobutan-2-ol hydrochloride by TBTU amide coupling (Bastiaans, et al., 1997) and subsequent Dess-Martin oxidation as described (Souček and Urban, 1995; Yin, et al., 2007). Peptidyl thiazolylketones were prepared by coupling of N-Boc-alanine to 2-bromothiazol as described (Dondoni and Perrone, 1993). Consequent deprotection and coupling to Ac-RVRH-OH was done using TCFH (tetramethylchloroformamidinium hexafluorophosphate) as reported (Tulla-Puche, et al., 2008). Peptidyl boronates were synthesised by coupling of (R)-Boroalanine-(1S,2S,3R,5S)-(+)-2,3-pinanediol ester hydrochloride to the protected Ac-RVRH-OH by the mixed anhydride method as described (Zoll, et al., 2014).

All compounds were characterised by mass spectrometry and NMR as documented in the next section. Analytical reversed-phase HPLC was run on a Reprosil-100, C18, 250×4.6 mm, 5 µm column (Watrex International, Inc, San Francisco, CA, USA) using gradients of acetonitrile (solvent B) and 0.1% (v/v) aqueous trifluoroacetic acid (solvent A), flow rate of 1 mL/min and UV detection at 210 nm. Preparative HPLC separations were performed on a YMC-Pack, ODS-AM, 250×20 mm, 5 µm column (YMC America, Inc., Allentown, PA, U.S.A.) using gradients of acetonitrile and 0.1% (v/v) aqueous trifluoroacetic acid (TFA) with UV detection at 210 – 235 nm at a flow rate of 10 mL/min. Mass-spectra were acquired on Waters Micromass ZQ ESCi multimode ionization mass-spectrometer and LTQ Orbitrap XL (Thermo Fisher Scientific) using ESI (+) ionization. LC-MS analysis was performed on Agilent Technologies Liquid Chromatograph coupled with TOF 6230 ESI-MS detector, using gradient of 2 to 100% solvent B over 10 min on a Waters 1.7 µm C18 , 100×2.1 mm column at a flow rate of 0.3 mL/min, where solvent A = 0.1% formic acid/water and solvent B = 0.1% formic acid/acetonitrile). NMR spectra were acquired on a Bruker AV-400 MHz spectrometer at room temperature.

Compound Characterization Data

<u>Peptidyl chlormethylketones</u> (Zoll, et al., 2014)

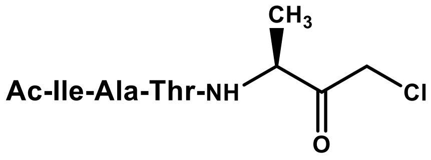

HPLC-analysis: Gradient 15-50 % B over 30 min; One peak at 13.3 min.

HR-MS: Monoisotopic mass 449.21672 calculated for $[M+H]^+$; found 449.21628.

^{1}H NMR (400 MHz, DMSO-d_6) δ (ppm) = 0.86 – 0.71(m, 6H, CH3), 1.04 (d, 3H, CH3, 3J = 6.4 Hz), 1.13 – 1.06 (m, 2H, CH2), 1.22 (d, 6H, CH3, J = 7.1 Hz), 1.47 – 1.35 (m, 1H, CH), 1.85 (s, 3H, acetyl), 4.06 – 3.93 (m, 1H, CH), 4.21 – 4.10 (m, 2H, CH), 4.40 – 4.28 (m, 2H, CH), 4.65 – 4.52 (m, 2H, COCH2Cl), 4.94 (s, 1H, OH), 7.6 (d, 1H, CONH, J = 7.9 Hz), 7.93 (d, 1H, CONH, J= 8.2 Hz), 8.23 (dd, 2H, CONH, J = 24.3, 6.9 Hz).

^{13}C NMR (101 MHz, DMSO-d_6) δ (ppm) = 201.17, 172.25, 171.12, 170.24, 169.33, 66.47, 58.04, 56.80, 52.37, 48.35, 47.45, 36.48, 24.36, 22.44, 19.78, 17.60, 15.70, 15.34, 10.98.

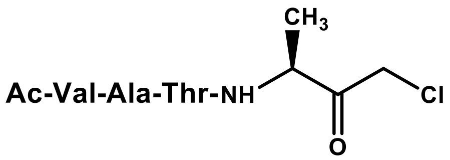

HPLC analysis: Gradient 2 – 30 % B over 30 min; One peak at 19.55 min.

ESI-MS: Monoisotopic mass: 434.2, calculated for $C_{18}H_{31}ClN_4O_6$; found $[M+H]^+$ 435.2, $[M+Na]^+$ 457.3.

^{1}H NMR (400 MHz, DMSO-d_6) δ (ppm) = 8.25 (d, J = 6.6 Hz, 1H, CONH), 8.17 (d, J = 7.1 Hz, 1H, CONH), 7.90 (d, J = 8.6 Hz, 1H, CONH), 7.59 (d, J = 8.2 Hz, 1H, CONH), 4.65 – 4.52 (m, 2H,COCH2Cl), 4.34 (td, J = 7.0, 3.9 Hz, 2H, CH), 4.19 – 4.08 (m, 2H, CH), 4.00 (dd, J = 6.3, 4.0 Hz, 1H, CH), 1.98 – 1.9 (m, 1H, CH), 1.86 (s, 3H, acetyl), 1.22 (d, J = 7.1 Hz, 6H, CH3), 1.04 (d, J = 6.3 Hz, 3H, CH3), 0.84 (dd, J = 8.2, 6.9 Hz, 6H, CH3).

^{13}C NMR (101 MHz, DMSO-d_6) δ (ppm) = 201.21, 172.32, 171.09, 170.27, 169.45, 66.50, 58.06, 57.68, 52.39, 48.33, 47.49, 30.37, 22.46, 19.83, 19.25, 18.19, 17.63, 15.73.

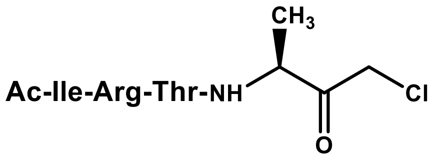

LC-MS analysis: One peak at 6.75 min, corresponding to the molecular mass $[M+H]^+$ 534.279.

HR-MS: Monoisotopic mass: 533.27286, calculated for $C_{22}H_{40}ClN_7O_6$, found $[M+H]^+$ 534.2804.

^{1}H NMR (400 MHz, DMSO-d_6) δ (ppm) = 8.33 (d, J = 6.5 Hz, 1H, CONH), 8.17 (d, J = 8.3 Hz, 1H, CONH), 7.96 (d, J = 8.6 Hz, 1H, CONH), 7.67 (d, J = 8.3 Hz, 1H, CONH), 7.53 – 7.44 (m, 1H, CONH), 4.97 (s, 1H, OH), 4.66 – 4.53 (m, 2H, $COCH_2Cl$), 4.39 – 4.26 (m, 2H, CH), 4.22 – 4.11 (m, 2H, CH), 4.06 – 3.95 (m, 1H, CH), 3.16 – 3.03 (m, 2H, CH_2), 1.85 (s, 3H, acetyl), 1.78 – 1.60 (m, 2H, CH_2), 1.60 – 1.36 (m, 5H, CH_2+CH), 1.22 (d, J = 7.1 Hz, 3H, CH_3), 1.04 (d, J = 6.3 Hz, 3H, CH_3), 0.86 – 0.70 (m, 7H, CH_3).

^{13}C NMR (101 MHz, DMSO-d_6) δ (ppm) = 201.27, 171.47, 170.26, 169.51, 156.64, 66.58, 57.99, 57.06, 52.49, 47.49, 36.34, 28.73, 25.03, 24.45, 22.44, 19.83, 15.71, 15.39, 10.97.

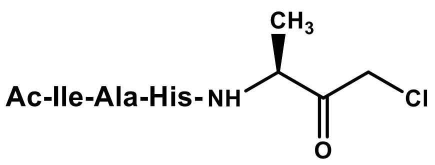

HPLC analysis: Two peaks at 6.84 and 6.94 min, both corresponding to the molecular mass $[M+H]^+$ 485.25 (diastereoisomers).

HR-MS: Monoisotopic mass 484.220096, calculated for $C_{21}H_{33}ClN_6O_5$, found $[M+H]^+$ 485.22729.

^{1}H NMR (400 MHz, DMSO-d_6) δ (ppm) = 14.15 (s, 1H, NH imidazole), 8.98 (dd, *J* = 4.3, 1.3 Hz, 1H, aromatic), 8.41 (d, *J* = 6.8 Hz, 1H, CONH), 8.34 – 8.24 (m, 1H, CONH), 8.19 (dd, *J* = 11.3, 7.0 Hz, 1H, CONH), 7.90 (dd, *J* = 8.3, 2.0 Hz, 1H, CONH), 7.36 (d, *J* = 11.3 Hz, 1H, aromatic), 4.60 (d, *J* = 2.5 Hz, 1H, $COCH_2Cl$), 4.56 (d, *J* = 5.5 Hz, 1H, $COCH_2Cl$), 4.53 – 4.48 (m, 1H, CH), 4.43 – 4.31 (m, 1H, CH), 4.22 – 4.04 (m, 2H, CH), 3.19 – 3.05 (m, 1H, CH_2), 3.02 – 2.90 (m, 1H, CH_2), 1.86 (d, *J* = 2.9 Hz, 3H, acetyl), 1.71 – 1.61 (m, 1H, CH), 1.44 – 1.36 (m, 1H, CH_2), 1.33 (s, 1H, CH_2), 1.25 – 1.15 (m, 5H, CH_3), 1.13 (d, *J* = 7.1 Hz, 2H, CH_3), 0.84 – 0.76 (m, 7H, CH_3).

^{13}C NMR (101 MHz, DMSO-d_6) δ (ppm) = 200.98, 172.42, 171.62, 170.20, 133.72, 130.17, 129.32, 116.89, 56.95, 53.58, 52.31, 51.44, 47.45, 36.25, 24.46, 22.50, 17.50, 17.23, 15.62, 15.26, 10.90.

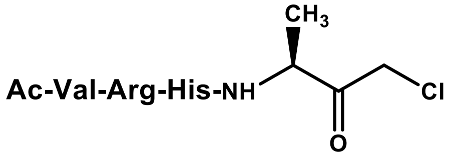

HPLC analysis: Gradient 2 – 30 % B over 30 min; One peak at 17.00 min.

ESI-MS: Monoisotopic mass: 555.27, calculated for $C_{23}H_{38}ClN_9O_5$; found $[M+H]^+$ 556.3, $[M+Na]^+$ 578.4.

^{1}H NMR (400 MHz, DMSO-d_6) δ (ppm) = 0.82 (d, *J* = 6.8 Hz, 6H, CH_3), 1.19 (d, *J* = 7.1 Hz, 3H), 1.58 – 1.34 (m, 3H, CH_2), 1.72 – 1.58 (m, 1H, CH_2), 1.87 (s, 3H, acetyl), 1.96 – 1.88 (m, 1H, CH_2), 2.95 (dd, *J* = 15.1, 7.5 Hz, 1H, CH_2), 3.17 – 3.01 (m, 3H, CH_2, CH), 4.14 – 4.02 (m, 1H, CH), 4.21 (q, *J* = 6.9 Hz, 1H, CH), 4.32 (q, *J* = 7.1 Hz, 1H, CH), 4.64 – 4.45 (m, 3H, $COCH_2Cl$ + CH), 7.35 (s, 1H, imidazole), 7.52 (t, *J* = 5.6 Hz, 1H, imidazole), 7.91 (d, *J* = 8.2 Hz, 1H, CONH), 8.12 (d, *J* = 7.6 Hz, 1H, CONH), 8.28 (d, *J* = 7.7 Hz, 1H, CONH), 8.49 (d, *J* = 6.5 Hz, 1H, CONH), 8.95 (s, 1H, NH), 14.15 (s, 1H, NH imidazole).

^{13}C NMR (101 MHz, DMSO-d_6) δ (ppm) = 200.79, 171.49, 169.91, 156.63, 135.99, 133.77, 58.07, 52.25, 51.41, 47.36, 30.07, 29.56, 28.59, 26.12, 25.02, 22.45, 19.13, 18.32, 15.54.

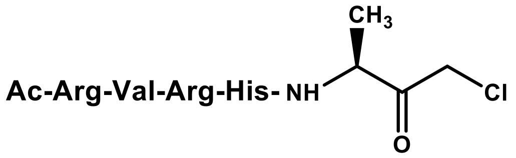

HPLC analysis: Gradient 2 – 30 % B over 30 min; One peak at 16.40 min.

HR-MS: Monoisotopic mass: 711.36955 calculated for $C_{29}H_{50}ClN_{13}O_6$; found $[M+H]^+$ 712.37716.

^{1}H NMR (400 MHz, DMSO-d_6) δ(ppm) = 0.80 (dd, *J* = 11.2, 6.8 Hz, 6H, CH_3), 1.19 (d, *J* = 7.1 Hz, 3H, CH_3), 1.57 – 1.36 (m, 6H, CH_2), 1.73 – 1.56 (m, 2H, CH_2), 1.87 (s, 3H, acetyl), 2.02 – 1.89 (m, 1H, CH), 2.95 (dd, *J* = 15.3, 8.0 Hz, 1H, CH), 3.08 (d, *J* = 4.6 Hz, 5H, CH_2, CH), 4.39 – 4.13 (m, 4H, CH), 4.61 – 4.48 (m, 3H, $COCH_2Cl$, CH), 7.34 (s, 1H, CH-imidazole), 7.74 – 7.55 (m, 3H, CONH + NH), 8.16 (d, *J* = 7.8 Hz, 2H, CONH), 8.34 (d, *J* = 7.5 Hz, 1H, CONH), 8.51 (d, *J* = 6.6 Hz, 1H, CONH), 8.94 (s, 1H, CH-imidazole), 14.26 (s, 1H).

^{13}C NMR (101 MHz, DMSO-d_6) δ (ppm) = 200.79, 171.44, 170.97, 169.94, 169.69, 156.72, 133.74, 129.11, 116.77, 57.21, 52.26, 51.42, 47.34, 30.71, 28.71, 25.03, 22.44, 21.57, 19.08, 17.91, 15.52.

Peptidyl sulfonamide

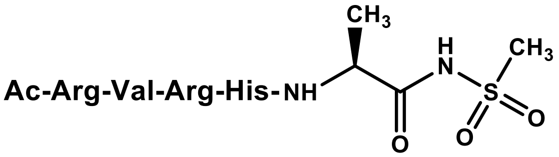

LC-MS analysis: One major peak at 1.47 min, corresponding to the mass $[M+H]^+$ 757.39.

HR-MS: Monoisotopic mass 756.381326 calculated for $C_{29}H_{52}N_{14}O_8S$, found $[M+H]^+$ 757.38868.

^{1}H NMR (401 MHz, DMSO-d_6) δ (ppm) = 8.95 (s, 1H, aromatic), 8.35 (d, *J* = 6.4 Hz, 1H CONH), 8.25 (d, *J* = 7.8 Hz, 1H, CONH), 8.15 (d, *J* = 7.8 Hz, 2H, CONH), 7.61 (t, *J* = 8.8 Hz, 3H, CONH), 7.32 (s, 1H, aromatic), 4.59 (q, *J* = 7.5 Hz, 1H, CH), 4.31 – 4.13 (m, 4H, CH), 3.21 (s, 3H, CH_3), 3.08 (d, *J* = 5.6 Hz, 5H, CH + CH_2), 2.95 (dd, *J* = 15.3, 7.8 Hz, 1H, CH), 2.00 – 1.89 (m, 1H, CH), 1.87 (s, 3H, $COCH_3$), 1.73 – 1.58 (m, 2H, CH_2), 1.57 – 1.35 (m, 6H, CH_2), 1.26 (d, *J* = 7.2 Hz, 3H, CH_3), 0.79 (dd, *J* = 11.7, 6.8 Hz, 6H, CH_3).

^{13}C NMR (101 MHz, DMSO-d_6) δ (ppm) = 171.52, 169.71, 158.56, 158.24, 156.71, 133.71, 124.59, 116.79, 115.53, 57.16, 52.42, 51.13, 48.92, 40.90, 40.42, 30.78, 28.73, 25.14, 22.47, 19.11, 17.90, 16.95.

Peptidyl thiazolylketone

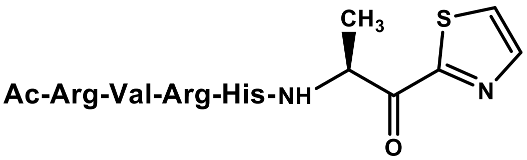

Peak 1 (used in this study)

HPLC analysis: Gradient 2-30 % B over 30 min. One peak at 19.23 min; ESI-MS $[M+H]^+$ 747.4.

HR-MS: Molecular mass 746.37588, calculated for $C_{31}H_{50}N_{14}O_6S$; found $[M+H]^+$ 747.38341.

^{1}H NMR (400 MHz, DMSO-d_6) δ (ppm) = 14.28 (s, 1H, NH imidazole), 9.08 (s, 1H, aromatic), 8.69 (d, *J* = 6.2 Hz, 1H, CONH), 8.44 – 8.35 (m, 2H, CONH), 8.35 – 8.21 (m, 3H, CONH+aromatic), 7.68 (dt, *J* = 13.0, 7.1 Hz, 3H, aromatic), 7.47 (d, *J* = 11.9 Hz, 1H, aromatic), 5.52 – 5.40 (m, 1H, CH), 4.76 (q, *J* = 7.4 Hz, 1H, CH), 4.43 – 4.24 (m, 3H, CH), 3.19 (dd, *J* = 12.2, 6.1 Hz, 5H, CH_2), 3.00 (s, 1H, CH_2), 2.04 (dd, *J* = 13.1, 6.4 Hz, 1H, CH), 1.97 (s, 3H, $COCH_3$), 1.84 – 1.52 (m, 8H, CH_2), 1.54 – 1.46 (m, 3H, CH_3), 0.90 (dd, *J* = 11.9, 6.7 Hz, 6H, CH_3).

Peak 2

HPLC analysis: Gradient 2-30 % B over 30 min. One peak at 20.01 min.

ESI-MS: $[M+H]^+$ 747.4.

HR-MS: Molecular mass 746.37588, calculated for $C_{31}H_{50}N_{14}O_6S$; found $[M+H]^+$ 747.38330.

^{1}H NMR (400 MHz, DMSO-d_6) δ (ppm) = 8.95 (s, 1H, aromatic), 8.46 (d, *J* = 6.1 Hz, 1H, CONH), 8.41 (d, *J* = 8.5 Hz, 1H, CONH), 8.34 (d, *J* = 6.3 Hz, 1H, CONH), 8.30 (d, *J* = 3.0 Hz, 1H, aromatic), 8.26 (d, *J* = 6.2 Hz, 1H, CONH), 8.21 (d, *J* = 3.0 Hz, 1H, CONH), 8.17 (s, 1H, aromatic), 7.65 – 7.52 (m, 3H, aromatic+NH_2), 7.33 (d, *J* = 5.7 Hz, 1H, aromatic), 5.45 – 5.30 (m, 1H, CH), 4.76 – 4.57 (m, 1H, CH), 4.30 – 4.05 (m, 3H, CH), 3.20 – 2.98 (m, 5H, CH_2), 2.91 (dd, *J* = 14.9, 9.3 Hz, 1H, CH_2), 1.90 (dt, *J* = 13.1, 6.5 Hz, 1H, CH), 1.84 (d, *J* = 5.0 Hz, 3H, $COCH_3$), 1.72 – 1.61 (m, 1H), 1.60 – 1.37 (m, 8H, CH_2), 0.80 (dd, *J* = 11.8, 6.8 Hz, 6H, CH_3).

^{13}C NMR (101 MHz, DMSO-d_6) δ (ppm) = 197.29, 194.51, 171.55, 169.31, 167.53, 158.06, 156.66, 133.84, 128.59, 107.86, 104.97, 63.92, 57.16, 50.62, 50.26, 48.84, 30.77, 25.12, 22.40, 19.04, 17.98, 16.72.

Peptidyl trifluoromethylketone

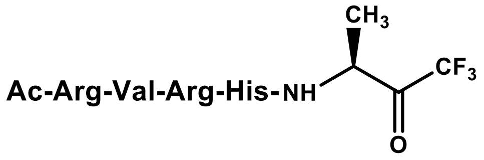

LC-MS analysis: Two peaks at 1.73 and 1.94 min, corresponding to the $[M+H]^+$ 732.39 (diastereomers). Peak 1 used in this study.

HR-MS: Molecular mass 731.38028, calculated for $C_{29}H_{48}F_3N_{13}O_6$, found $[M+H]^+$ 732.38776 and $[M+Na]^+$ 754.36969.

^{1}H NMR (401 MHz, DMSO-d_6) δ (ppm) = 14.22 (s, 2H, NH imidazole), 8.97 (s, 1H, aromatic), 8.28 (d, *J* = 8.0 Hz, 1H, CONH), 8.21 (d, *J* = 8.2 Hz, 1H, CONH), 8.19-8.10 (s, 2H, CONH), 7.82 (d, *J* = 9.3 Hz, 1H, CONH), 7.69 (d, *J* = 9.5 Hz, 1H, CONH), 7.64 – 7.50 (m, 3H, NH+NH_2), 7.36 (s, 1H, aromatic), 7.28 (s, 1H, NH), 7.06 (s, 2H, NH_2), 4.69 – 4.58 (m, 1H, CH), 4.33 – 4.07 (m, 4H, CH), 3.05 (ddt, *J* = 20.0, 14.9, 7.4 Hz, 5H, CH+CH_2), 2.92 – 2.82 (m, 1H, CH), 2.00 - 1.90 (m, 1H, CH), 1.87 (s, 3H, $COCH_3$), 1.63 (d, *J* =

5.4 Hz, 2H, CH_2), 1.46 (dt, *J* = 19.0, 10.3 Hz, 6H, CH_2), 1.08 (d, *J* = 6.8 Hz, CH_3), 1.00 (d, *J* = 6.8 Hz, CH_3), 0.78 (ddd, *J* = 11.1, 6.7, 2.5 Hz, 6H, CH_3).

^{13}C NMR (101 MHz, DMSO-d_6) δ (ppm) = 207.88, 172.52, 171.94, 169.77, 166.63, 158.63, 157.16, 152.24, 117.20, 113.16, 57.57, 52.85, 31.29, 27.23, 25.40, 22.94, 19.58, 18.35.

<u>Peptidyl boronate</u>

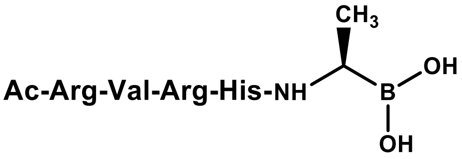

Peak 1

HPLC analysis: Gradient 2-30 % B over 30 min; One peak at 13.43 min

ESI-MS: Monoisotopic mass: 679.40, calculated for $C_{27}H_{50}BN_{13}O_7$, found $[M+H]^+$ 680.1, (diastereomer).

^{1}H NMR (401 MHz, DMSO-d_6) δ (ppm) = 14.20 (s, 1H, NH imidazole), 8.95 (s, 1H, aromatic), 8.18 (dd, *J* = 24.1, 7.7 Hz, 3H, CONH), 7.92 (t, *J* = 5.5 Hz, 1H, CONH), 7.60 (dt, *J* = 10.4, 5.0 Hz, 3H, NH_2+NH), 7.33 (d, *J* = 5.4 Hz, 2H, aromatic+NH), 4.48 (dq, *J* = 14.4, 7.6 Hz, 1H, CH), 4.30 – 4.23 (m, 1H, CH), 4.19 (q, *J* = 9.5, 8.2 Hz, 2H, CH), 3.06 (tt, *J* = 16.0, 7.1 Hz, 6H, CH_2+CH), 2.93 (dq, *J* = 15.0, 7.6 Hz, 2H,CH_2), 1.96 (dd, *J* = 13.5, 6.7 Hz, 1H, CH), 1.87 (s, 3H, $COCH_3$), 1.74 – 1.58 (m, 2H, CH_2), 1.56 – 1.35 (m, 6H, CH_2), 1.03 (d, *J* = 7.6 Hz, 1H, CH_3), 0.95 (t, *J* = 7.2 Hz, 2H, CH_3), 0.80 (ddd, *J* = 11.5, 6.7, 2.4 Hz, 6H, CH_3).

Peak 2 (used in this study)

HPLC analysis: Gradient 2-30 % B over 30 min; One peak at 14.12 min

ESI-MS: Monoisotopic mass: 679.40, calculated for $C_{27}H_{50}BN_{13}O_7$, found $[M+H]^+$ 680.1, (diastereomer).

^{1}H NMR (401 MHz, DMSO-d_6) δ (ppm) = 8.93 (s, 1H, aromatic), 8.40 (d, *J* = 8.5 Hz, 1H, CONH), 8.29 (d, *J* = 6.1 Hz, 1H, CONH), 8.18 (dt, *J* = 13.4, 6.9 Hz, 2H, CONH), 7.85 (t, *J* = 5.5 Hz, 1H, CONH), 7.60 (d, *J* = 7.4 Hz, 2H, NH_2), 7.32 (d, *J* = 7.7 Hz, 1H, aromatic), 4.61 – 4.45 (m, 1H, CH), 4.37 – 4.13 (m, 3H, CH), 4.14 – 4.03 (m, 1H, CH), 3.22 – 3.01 (m, 6H, CH_2), 3.00 – 2.82 (m, 2H, CH_2), 2.02 – 1.90 (m, 1H, CH), 1.88 (s, 3H, $COCH_3$), 1.75 – 1.58 (m, 2H, CH_2), 1.49 (dt, *J* = 17.7, 8.1 Hz, 6H, CH_2), 1.27 – 1.09 (m, 1H, CH_3), 1.07 – 0.91 (m, 2H, CH_3), 0.88 – 0.69 (m, 6H, CH_3).

<u>Peptidyl ketoamides</u>

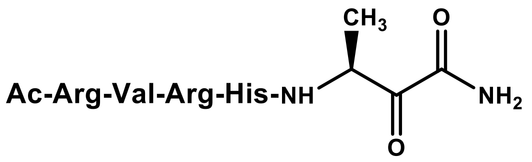

Compound ***1***

LC-MS analysis: Gradient 2 – 30 % B over 7 min; Three diastereomeric peaks at 1.85, 2.03 and 2.2 min, corresponding to $[M+H]^+$ 707.4.

HR-MS: Monoisotopic mass: 706.40, calculated for $C_{29}H_{50}N_{14}O_7$; found $[M+H]^+$ 707.40638 and $[M+2H]^{2+}$ 354.20689.

Insufficient amount for quality NMR.

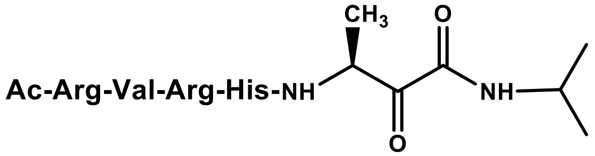

Compound ***2***

HPLC analysis: Gradient 2 – 100 % B over 36 min.; One peak at 13.45 min.

ESI-MS: $[M+H]^+$ 749.6, $[M+Na]^+$ 771.6, $[M+2H]^{2+}$ 375.3.

HR-MS: Monoisotopic mass: 748.45, calculated for $C_{32}H_{56}N_{14}O_7$; found $[M+H]^+$ 749.45324, $[M+Na]^+$ 771.43518 and $[M+2H]^{2+}$ 375.23028.

^{1}H NMR (401 MHz, DMSO-d_6) δ (ppm) = 8.93 (s, 1H, COCONH), 8.53 (d, J = 8.3 Hz, 1H, aromatic), 8.43 (d, J = 6.3 Hz, 1H, CONH), 8.25 (d, J = 7.9 Hz, 1H, CONH), 8.21 – 8.11 (m, 2H, CONH), 7.74 – 7.58 (m, 3H, CONH + NH_2), 7.33 (s, 1H, aromatic), 5.13 – 4.93 (m, 1H, CH), 4.69 – 4.56 (m, 1H, CH), 4.36 – 4.13 (m, 3H, CH), 3.93 (dt, J = 14.8, 7.3 Hz, 1H, CH), 3.09 (d, J = 5.6 Hz, 5H, CH+ CH_2), 3.00 – 2.85 (m, 1H, CH), 1.97 (dd, J = 15.0, 8.3 Hz, 1H, CH), 1.88 (s, 3H, $COCH_3$), 1.73 – 1.58 (m, 2H, CH_2), 1.49 (dt, J = 15.2, 9.0 Hz, 6H, CH_2), 1.26 (d, J = 7.3 Hz, 3H, CH_3), 1.11 (d, J = 6.6 Hz, 6H, CH_3), 0.80 (dd, J = 11.5, 6.7 Hz, 6H, CH_3).

^{13}C NMR (101 MHz, DMSO-d_6) δ (ppm) = 187.06, 171.49, 171.27, 159.60, 156.72, 135.61, 133.76, 115.70, 104.52, 57.14, 52.38, 49.54, 30.78, 28.71, 25.07, 22.46, 21.79, 19.10, 17.88, 15.61.

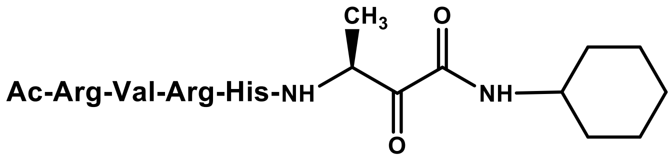

Peak 1 (compound ***3***)

LC - MS analysis: One peak at 5.87 min with the corresponding mass of the product $[M+H]^+$ 789.48.

HR-MS: Monoisotopic mass: 788.4769 calculated for $C_{35}H_{60}N_{14}O_7$ found: $[M+2H]^{2+}$ 395.24606 and $[M+3H]^{3+}$ 263.83329.

^1H NMR (401 MHz, DMSO-d_6) δ (ppm) = 14.30 (s, 2H, NH), 9.03 – 8.93 (m, 1H, CONH), 8.55 – 8.47 (m, 1H, aromatic), 8.42 (t, *J* = 6.4 Hz, 1H, CONH), 8.25 (d, *J* = 8.1 Hz, 1H, CONH), 8.16 (q, *J* = 6.7 Hz, 2H, CONH), 7.64 (dtd, *J* = 14.0, 8.7, 4.9 Hz, 3H, CONH), 7.39 – 7.31 (m, 1H, aromatic), 5.02 (tt, *J* = 9.7, 5.8 Hz, 1H, CH), 4.62 (td, *J* = 8.1, 5.6 Hz, 1H, CH), 4.31 – 4.22 (m, 1H, CH), 4.19 (dtd, *J* = 11.2, 6.2, 2.9 Hz, 2H, CH), 3.57 (dt, *J* = 19.8, 6.2 Hz, 1H, CH), 3.07 (q, *J* = 6.4 Hz, 5H, CH_2), 2.96 – 2.83 (m, 1H, CH_2 His), 2.02 – 1.89 (m, 1H, CH), 1.87 (d, *J* = 2.2 Hz, 3H, acetyl), 1.74 – 1.58 (m, 6H, CH_2), 1.49 (dtd, *J* = 25.9, 15.5, 14.7, 6.3 Hz, 7H, CH_2), 1.26 (dq, *J* = 14.3, 11.4, 10.8 Hz, 7H), 0.79 (dd, *J* = 11.4, 6.8 Hz, 6H, CH_3).

^{13}C NMR (101 MHz, DMSO-d_6) δ (ppm) = 171.49, 171.28, 169.62, 158.58 , 158.26 , 156.73 , 133.72, 129.06, 115.37, 57.16, 52.40, 49.59, 48.07, 31.78, 30.78, 28.75, 25.07, 24.73, 22.46, 19.10, 17.89, 15.60.

Peak 2

LC-MS analysis: One peak at 6.18 min with the corresponding mass of the product $[M+H]^+$ 789.48.

HR-MS: Monoisotopic mass: 788.4769 calculated for $C_{35}H_{60}N_{14}O_7$ found: $[M+2H]^{2+}$ 395.24567 and $[M+3H]^{3+}$ 263.83298.

^1H NMR (401 MHz, DMSO-d_6) δ (ppm) = 14.23 (s, 1H, NH), 9.05 – 8.93 (m, 1H, CONH), 8.51 (d, *J* = 8.3 Hz, 1H, aromatic), 8.40 (q, *J* = 9.3, 7.9 Hz, 1H, CONH), 8.30 – 8.11 (m, 3H, CONH), 7.67 – 7.53 (m, 2H, CONH), 7.35 (d, *J* = 4.0 Hz, 1H, aromatic), 5.07 – 4.97 (m, 1H, CH), 4.70 – 4.56 (m, 1H, CH), 4.25 (qd, *J* = 9.8, 8.4, 3.8 Hz, 1H, CH), 4.22 – 4.05 (m, 2H, CH), 3.65 – 3.50 (m, 1H, CH), 3.18 – 2.98 (m, 5H, CH_2), 2.90 (td, *J* = 14.7, 13.5, 7.3 Hz, 1H, CH_2), 1.87 (dd, *J* = 6.1, 2.0 Hz, 3H, acetyl), 1.76 – 1.62 (m, 5H, CH_2), 1.63 – 1.39 (m, 7H, CH_2), 1.39 – 1.15 (m, 8H, CH_2, CH_3), 1.16 -1.00 (m, 2H, CH_2), 0.80 (ddd, *J* = 11.7, 7.1, 4.6 Hz, 6H, CH_3).

^{13}C NMR (101 MHz, DMSO-d_6) δ (ppm) = 195.26, 188.02, 158.45, 156.68, 140.04, 115.29, 64.61, 48.07, 46.50, 43.74, 31.78, 30.72, 28.43, 26.91, 24.74, 22.45, 19.06, 18.00.

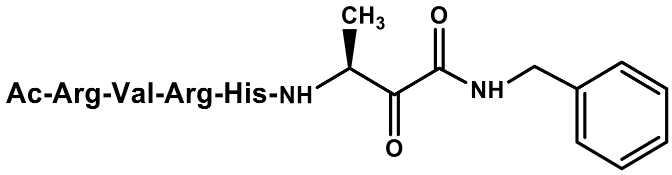

Peak 1

LC-MS analysis: One peak at 5.81 min with the corresponding mass of the product $[M+H]^+$ 797.45.

HR-MS: Monoisotopic mass: 796.4456 calculated for $C_{36}H_{56}N_{14}O_7$ found: $[M+H]^+$ 797.45301.

^{1}H NMR (401 MHz, DMSO-d_6) δ 14.27 (s, 1H, NH imidazole), 8.94 (d, *J* = 8.1 Hz, 1H, aromatic), 8.55 – 8.39 (m, 1H, CONH), 8.24 (t, *J* = 7.3 Hz, 1H, CONH), 8.21 – 8.12 (m, 1H, CONH), 7.75 – 7.55 (m, 3H, CONH), 7.49 – 7.15 (m, 8H, aromatic+NH_2), 5.08 – 4.97 (m, 1H, CH), 4.69 – 4.55 (m, 1H, CH), 4.41 – 4.06 (m, 5H, CH + CH_2), 3.19 (dd, *J* = 15.3, 5.2 Hz, 1H, CH_2 His), 3.06 (dq, *J* = 12.7, 6.2 Hz, 4H, CH_2), 2.92 (dd, *J* = 15.2, 9.0 Hz, 1H, CH_2 His), 1.98 – 1.80 (m, 4H, + CH), 1.73 – 1.57 (m, 2H, CH_2), 1.47 (dt, *J* = 15.9, 8.0 Hz, 5H, CH_2), 1.37 – 1.14 (m, 3H, CH_3), 0.87 – 0.69 (m, 6H, CH_3).

Peak 2 (compound ***4*****)**

LC-MS analysis: One peak at 6.07 min with the corresponding mass of the product $[M+H]^+$ 797.45.

HR-MS: Monoisotopic mass: 796.4456 calculated for $C_{36}H_{56}N_{14}O_7$ found: $[M+H]^+$ 797.45282.

Yield: 1.3 mg (4.2%).

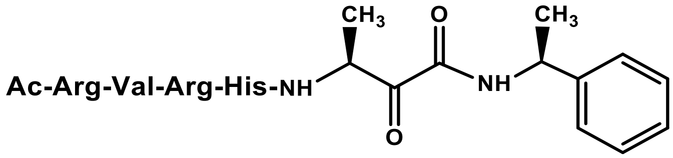

Peak 1 (compound ***5***)

HPLC analysis: One peak at 14.2 min Gradient 2 – 100 % B over 36 min.

ESI-MS: $[M+H]^+$ 811.5.

HR-MS: Monoisotopic mass: 810.46129, calculated for $C_{37}H_{58}N_{14}O_7$ found: $[M+H]^+$ 811.46909 and $[M+2H]^{2+}$ 406.23814.

^{1}H NMR (401 MHz, DMSO-d_6) δ (ppm) = 14.30 (s, 1H, NH), 9.23 – 9.10 (m, 1H, CONH), 8.97 (s, 1H, aromatic), 8.45 (dd, *J* = 14.2, 6.5 Hz, 1H, CONH), 8.19 (dd, *J* = 31.1, 7.9 Hz, 3H, CONH), 7.61 (s, 3H), 7.34 (dt, *J* = 9.9, 4.3 Hz, 6H, Ar), 7.28 – 7.20 (m, 1H, CONH), 4.98 (dt, *J* = 15.2, 7.5 Hz, 2H, CH), 4.61 (q, *J* = 7.8 Hz, 1H, CH), 4.22 (dq, *J* = 33.3, 7.4, 5.5 Hz, 3H, CH), 3.18 – 2.98 (m, 5H, CH_2), 2.87 (dd, *J* = 15.7, 8.2 Hz, 1H, CH_2), 1.93 (dd, *J* = 12.7, 6.0 Hz, 1H, CH), 1.87 (s, 3H, acetyl), 1.72 – 1.58 (m, 2H, CH_2), 1.43 (td, *J* = 25.5, 22.6, 8.5 Hz, 9H, CH_2, CH_3), 1.27 (d, *J* = 7.3 Hz, 2H, CH_3), 1.16 (d, *J* = 7.1 Hz, 1H, CH_3), 0.79 (dt, *J* = 10.9, 5.7 Hz, 6H, CH_3).

^{13}C NMR (101 MHz, DMSO-d_6) δ (ppm) = 198.27, 173.20, 171.62, 169.73, 156.75, 139.20, 128.31, 126.18, 104.52, 67.45, 59.85, 52.41, 51.24, 48.24, 47.47, 28.75, 25.13, 22.46, 21.70, 19.09, 17.87, 15.54.

Peak 2

HPLC analysis: One peak at 14.6 min; Gradient 2 – 100 % B over 36 min.

ESI-MS: $[M+H]^+$ 811.4.

HR-MS: Monoisotopic mass: 810.46129, calculated for $C_{37}H_{58}N_{14}O_7$ found: $[M+H]^+$ 811.46901 and $[M+2H]^{2+}$ 406.23809.

^{1}H NMR (401 MHz, DMSO-d_6) δ (ppm) = 9.20 – 9.11 (m, 1H, CONH), 8.93 (s, 1H, aromatic), 8.51 – 8.30 (m, 1H, CONH), 8.31 – 8.11 (m, 3H, CONH), 7.62 (s, 3H, NH_2), 7.47 – 7.28 (m, 6H, aromatic), 7.28 – 7.18 (m, 1H, CONH), 5.04 – 4.91 (m, 1H, CH), 4.71 – 4.52 (m, 1H, CH), 4.35 – 4.06 (m, 3H, CH), 3.17 – 2.97 (m, 4H, CH_2), 2.96 – 2.80 (m, 1H, CH_2), 1.90 – 1.80 (m, 3H, acetyl), 1.73 – 1.60 (m, 2H, CH_2), 1.44 (d, J = 17.1 Hz, 9H, CH_2, CH_3), 1.26 (dd, J = 14.0, 7.3 Hz, 3H, CH_3), 0.79 (dd, J = 10.3, 6.7 Hz, 6H, CH_3).

^{13}C NMR (101 MHz, DMSO-d_6) δ (ppm) = 198.92, 170.15, 162.64, 158.45, 156.70, 138.57, 128.30, 126.91, 126.18, 118.63, 117.47, 61.95, 56.71, 53.49, 49.73, 48.18, 28.71, 25.13, 22.45, 21.66, 19.07, 17.99.

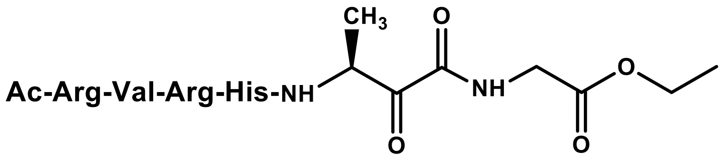

Compound ***6***

HPLC analysis: Gradient 2 – 30 % B over 30 min; One peak at 12.4 min.

ESI-MS: $[M+H]^+$ 792.4, $[M+2H]^{2+}$ 397.2.

HR-MS: Monoisotopic mass: 792.43552, calculated for $C_{33}H_{56}N_{14}O_9$; found: $[M+H]^+$ 793.44299, $[M+2H]^{2+}$ 397.22539.

^{1}H NMR (401 MHz, DMSO-d_6) δ (ppm) = 14.17 (s, 1H, NH imidazole), 9.03 (t, J = 5.9 Hz, 1H, CONH), 8.45 (d, J = 6.3 Hz, 1H, aromatic), 8.26 (d, J = 7.5 Hz, 1H, CONH), 8.15 (d, J = 7.8 Hz, 2H, CONH), 7.58 (t, J = 9.1 Hz, 2H, CONH), 7.37 – 7.26 (m, 1H, aromatic), 5.01 (p, J = 6.4, 5.8 Hz, 1H, CH), 4.61 (q, J = 8.0 Hz, 1H, CH), 4.35 – 4.05 (m, 5H, CH +CH_2), 3.90 (d, J = 6.8 Hz, 2H, CH_2), 3.19 – 2.97 (m, 5H, CH_2), 2.91 (dd, J = 15.2, 8.0 Hz, 1H, CH_2), 1.98 – 1.90 (m, 1H, CH), 1.87 (s, 3H, acetyl), 1.74 – 1.58 (m, 2H, CH_2), 1.49 (dq, J = 16.1, 7.7 Hz, 6H, CH_2), 1.31 – 1.12 (m, 6H, CH_3), 0.79 (dd, J = 11.9, 6.7 Hz, 6H, CH_3).

^{13}C NMR (101 MHz, DMSO-d_6) δ (ppm) = 168.83, 166.37, 164.30, 162.00, 156.67, 146.64, 139.66, 135.98, 134.39, 123.99, 60.73, 57.15, 53.86, 52.36, 49.54, 45.52, 28.75, 22.47, 21.18, 19.10, 14.05.

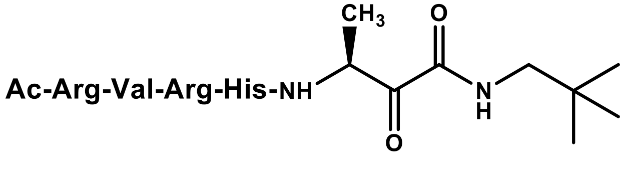

Compound **7**

LC-MS analysis: One peak at 5.66 min, corresponding to $[M+H]^+$ 777.5, $[M+2H]^{2+}$ 389.3, $[M+3H]^{3+}$ 259.85.

HR-MS: Monoisotopic mass: 776.476941, calculated for $C_{34}H_{60}N_{14}O_7$; found $[M+H]^+$ 777.48454, $[M+Na]^+$ 799.46650 and $[M+2H]^{2+}$ 389.24594.

^1H NMR (401 MHz, DMSO-d_6) δ (ppm) = 14.29 (s, 1H, NH imidazole), 8.94 (s, 1H, COCONH), 8.57 (s, 1H, aromatic), 8.42 (d, *J* = 6.4 Hz, 1H, CONH), 8.25 (d, *J* = 8.1 Hz, 1H, CONH), 8.15 (t, *J* = 7.4 Hz, 2H, CONH), 7.64 (dd, *J* = 21.6, 10.8 Hz, 2H, CONH+NH), 7.33 (s, 1H, aromatic), 5.12 – 4.94 (m, 1H,CH), 4.68 – 4.56 (m, 1H, CH), 4.35 – 4.12 (m, 3H, CH), 3.18 – 3.00 (m, 5H, CH_2), 2.96 (d, *J* = 6.8 Hz, 3H, CH_2), 2.03 – 1.90 (m, 1H, CH), 1.87 (s, 3H, $COCH_3$), 1.65 (dt, *J* = 16.3, 8.0 Hz, 2H, CH_2), 1.47 (dq, *J* = 24.5, 12.2, 10.4 Hz, 6H, CH_2), 1.23 (dd, *J* = 17.4, 7.3 Hz, 3H, CH_3), 0.94 – 0.63 (m, 15H, CH_3).

^{13}C NMR (101 MHz, DMSO-d_6) δ (ppm) = 196.92, 171.82, 171.51, 169.70, 161.17, 158.66, 156.75, 133.76, 57.16, 52.42, 51.05, 49.54, 32.62, 30.76, 28.75, 27.32, 25.14, 22.46, 19.10, 17.88, 15.46.

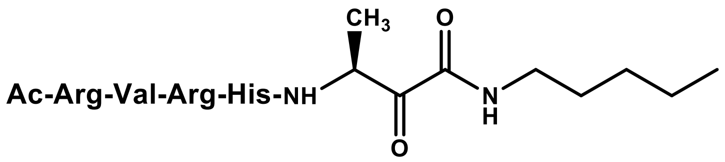

Peak 1 (compound **8**)

HPLC analysis: One peak at 14.34 min, Gradient 2 – 100 % B over 36 min.

ESI-MS: $[M+H]^+$ 777.5, $[M+Na]^+$ 799.5.

HR-MS: Monoisotopic mass: 776.476941, calculated for $C_{34}H_{60}N_{14}O_7$; found: $[M+H]^+$ 777.48440, $[M+2H]^{2+}$ 389.24586.

^1H NMR (401 MHz, DMSO-d_6) δ (ppm) = 14.20 (s, 1H, NH imidazole), 8.91 (s, 1H, CONH), 8.68 (t, *J* = 5.8 Hz, 1H, CONH), 8.41 (d, *J* = 6.4 Hz, 1H, aromatic), 8.20 (dd, *J* = 35.0, 7.4 Hz, 3H, CONH), 7.61 (d, *J* = 6.4 Hz, 3H, CONH + NH_2), 7.32 (s, 2H, NH_2), 5.02 (p, *J* = 7.2 Hz, 1H, CH), 4.61 (q, *J* = 7.8, 7.0 Hz, 1H, CH), 4.22 (dt, *J* = 31.2, 7.7 Hz, 3H, CH), 3.09 (dq, *J* = 12.1, 6.9 Hz, 7H, CH_2), 2.98 – 2.82 (m, 1H, CH_2), 2.18 – 2.03 (m, 1H, CH), 1.87 (s, 3H, acetyl), 1.64 (td, *J* = 14.1, 12.8, 7.1 Hz, 2H, CH_2), 1.58 – 1.34 (m, 8H, CH_2), 1.23 (tt, *J* = 15.5, 7.3 Hz, 7H, CH_2 + CH_3), 0.93 – 0.67 (m, 9H, CH_3).

Peak 2

HPLC analysis: One peak at 14.7 min, Gradient 2 – 100 % B over 36 min.

ESI-MS: $[M+H]^+$ 777.5, $[M+2H]^{2+}$ 389.2

HR-MS: Monoisotopic mass: 776.476941, calculated for $C_{34}H_{60}N_{14}O_7$; found: $[M+H]^+$ 777.48473, $[M+2H]^{2+}$ 389.24590.

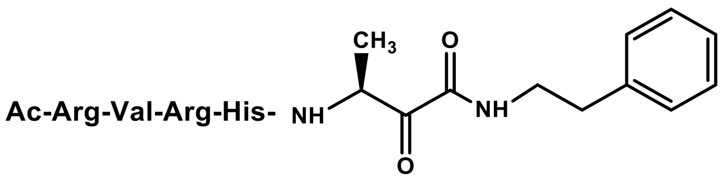

Compound ***9***

HPLC analysis: Gradient 2 – 30 % B over 30 min; One peak at 22.2 min.

ESI-MS: $[M+H]^+$ 811.3, $[M+2H]^{2+}$ 406.2

HR-MS: Monoisotopic mass 810.461290, calculated for $C_{37}H_{58}N_{14}O_7$; found: $[M+H]^+$ 811.46886, $[M+2H]^{2+}$ 406.23800.

^{1}H NMR (401 MHz, DMSO-d_6) δ (ppm) = 14.20 (s, 1H, NH imidazole), 8.93 (s, 1H, NH), 8.77 (t, *J* = 5.9 Hz, 1H, CONH), 8.41 (d, *J* = 6.2 Hz, 1H, aromatic), 8.25 (d, *J* = 7.9 Hz, 1H, CONH), 8.15 (t, *J* = 7.4 Hz, 2H, CONH), 7.62 (t, *J* = 10.9 Hz, 3H, CONH), 7.30 (dd, *J* = 14.5, 6.8 Hz, 5H, aromatic), 7.20 (d, *J* = 7.6 Hz, 5H, aromatic + NH_2), 5.01 (p, *J* = 7.2 Hz, 1H, CH), 4.62 (q, *J* = 8.0 Hz, 1H, CH), 4.34 – 4.12 (m, 3H, CH), 3.16 – 2.99 (m, 5H, CH_2), 2.91 (dd, *J* = 15.3, 8.1 Hz, 1H, CH_2), 2.77 (t, *J* = 7.5 Hz, 2H, CH_2), 2.00 – 1.90 (m, 1H, CH), 1.87 (s, 3H, acetyl), 1.73 – 1.57 (m, 2H, CH_2), 1.48 (dt, *J* = 19.0, 11.3 Hz, 6H, CH_2), 1.20 (d, *J* = 7.3 Hz, 3H, CH_3), 0.79 (dd, *J* = 11.0, 6.8 Hz, 6H, CH_3).

^{13}C NMR (101 MHz, DMSO-d_6) δ (ppm) = 196.62, 171.49, 171.28, 169.69, 169.59, 160.23, 158.33, 156.73, 138.95, 133.74, 128.60, 128.35, 126.21, 118.66, 57.16, 55.97, 52.41, 51.05, 49.45, 40.41, 34.57, 30.78, 28.74, 25.14, 22.47, 19.11, 17.89, 15.50.

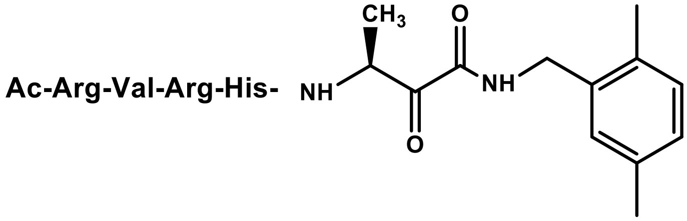

Compound ***10***

HPLC analysis: One peak at 6.35 min, corresponding to $[M+H]^+$ 825.5, $[M+2H]^{2+}$ 413.3, $[M+3H]^{3+}$ 275.84.

HR-MS: Monoisotopic mass: 824.47694, calculated for $C_{38}H_{60}N_{14}O_7$ found: $[M+H]^+$ 825.48462, $[M+2H]^{2+}$ 413.24584.

^1H NMR (401 MHz, DMSO-d_6) δ (ppm) = 14.25 (s, 1H, NH imidazole), 9.09 (t, J = 6.0 Hz, 1H, CONH), 8.95 (s, 1H, NH), 8.45 (d, J = 6.3 Hz, 1H, aromatic), 8.25 (d, J = 8.1 Hz, 1H, CONH), 8.15 (t, J = 6.7 Hz, 2H, CONH), 7.62 (dt, J = 13.8, 6.6 Hz, 2H, CONH), 7.33 (s, 1H, aromatic), 7.10 – 6.89 (m, 3H, aromatic), 5.04 (p, J = 7.2 Hz, 1H, CH), 4.61 (q, J = 7.9 Hz, 1H, CH), 4.33 – 4.11 (m, 5H, CH+CH_2), 3.16 – 3.00 (m, 5H, CH_2), 2.90 (dd, J = 15.1, 7.9 Hz, 1H, CH_2), 2.22 (s, 6H, CH_3),1.95 (dt, J = 13.3, 8.1 Hz, 1H, CH) , 1.87 (s, 3H, acetyl), 1.72 – 1.58 (m, 2H, CH_2), 1.57 – 1.33 (m, 6H, CH_2), 1.27 (d, J = 7.3 Hz, 3H, CH_3), 0.79 (dd, J = 11.1, 6.8 Hz, 6H, CH_3).

^{13}C NMR (101 MHz, DMSO-d_6) δ (ppm) = 200.01, 171.50, 171.30. 170.98, 169.70, 165.73, 160.58, 158.48, 156.72, 135.75, 134.51, 133.74, 129.93, 128.40, 127.59, 57.15, 52.41, 51.06, 49.58, 30.77, 28.71, 25.06, 22.46, 20.68, 19.10, 18.08, 15.53.

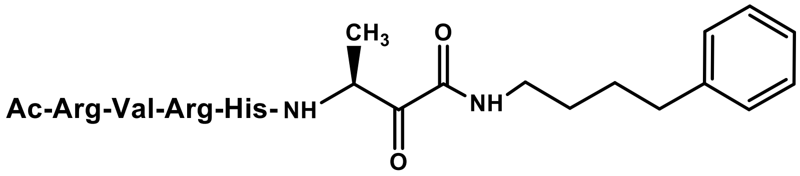

Compound ***11***

HPLC analysis: Gradient 2 – 100 % B over 36 min; One peak at 16.0 min.

ESI-MS: $[M+H]^+$ 839.5, $[M+2H]^{2+}$ 420.3.

HR-MS: Monoisotopic mass: 838.49263 calculated for $C_{39}H_{62}N_{14}O_7$; found $[M+H]^+$ 839.5007, $[M+2H]^{2+}$ 420.25366.

^1H NMR (401 MHz, DMSO-d_6) δ (ppm) = 14.17 (s, 1H, NH imidazole), 8.88 (s, 1H, COCONH), 8.71 (t, J = 5.8 Hz, 1H, aromatic), 8.41 (d, J = 6.4 Hz, 1H, CONH), 8.24 (d, J = 7.8 Hz, 1H, aromatic, CONH), 8.15 (d, J = 7.6 Hz, 2H, CONH), 7.59 (d, J = 6.2 Hz, 3H CONH), 7.27 (dd, J = 14.8, 7.1 Hz, 4H, aromatic), 7.18 (d, J = 7.9 Hz, 3H, aromatic), 5.08 – 4.95 (m, 1H, CH), 4.61 (q, J = 8.2, 7.0 Hz, 1H,CH), 4.22 (dt, J = 30.8, 8.0 Hz, 3H, CH), 3.11 (dd, J = 28.1, 6.1 Hz, 7H, CH_2), 2.99 – 2.84 (m, 1H, CH_2), 2.56 (d, J = 7.5 Hz, 3H, CH_2), 2.00 – 1.89 (m, 1H, CH), 1.87 (s, 3H, $COCH_3$), 1.75 – 1.33 (m, 12H, CH_2), 1.21 (dd, J = 18.8, 7.3 Hz, 3H, CH_3), 0.79 (dd, J = 11.5, 6.8 Hz, 6H, CH_3).

^{13}C NMR (101 MHz, DMSO-d_6) δ (ppm) = 207.16, 187.80, 169.69, 160.34, 156.70, 142.01, 128.27, 125.68, 71.09, 69.06, 57.12, 52.39, 44.45, 34.68, 28.29, 25.05, 22.46, 19.12, 17.87.

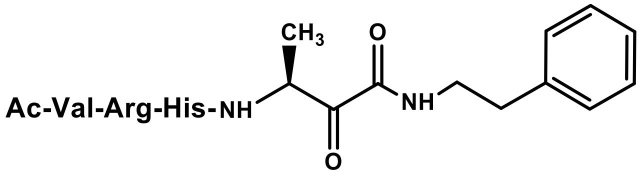

Compound ***12***

LC-MS analysis: One peak at 5.70 min, corresponding to $[M+H]^+$ 655.37 and $[M+2H]^{2+}$ 328.19.

HR-MS: Monoisotopic mass: 654.360179 calculated for $C_{31}H_{46}N_{10}O_6$, found $[M+H]^+$ 655.36774 and $[M+Na]^+$ 677.34979.

Yield: 1.5 mg (14 %).

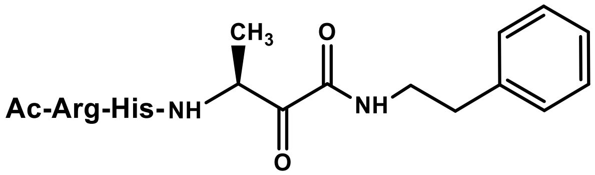

Compound ***13***

LC-MS analysis: One peak at 5.97 min, corresponding to $[M+H]^+$ 556.30 and $[M+2H]^{2+}$ 278.65.

HR-MS: Monoisotopic mass: 555.291765 calculated for $C_{26}H_{37}N_9O_5$, found $[M+H]^+$ 556.29939.

^{1}H NMR (401 MHz, DMSO-d_6) δ (ppm) = 14.14 (s, 2H, NH imidazole), 8.95 (s, 1H, aromatic), 8.77 (t, *J* = 4.7 Hz, 1H, COCONH), 8.38 – 8.08 (m, 3H, CONH), 7.54 (t, *J* = 5.3 Hz, 1H, aromatic), 7.41 – 7.25 (m, 4H, aromatic+NH_2), 7.25 – 7.16 (m, 3H, aromatic), 5.01 (p, *J* = 7.3 Hz, 1H, CH), 4.61 (t, *J* = 11.0 Hz, 1H, CH), 4.17 (dt, *J* = 13.4, 6.5 Hz, 1H, CH), 3.16 – 3.00 (m, 3H, CH_2 + CH), 2.92 (dt, *J* = 15.8, 8.3 Hz, 1H, CH), 2.77 (t, *J* = 7.7 Hz, 2H, CH_2), 1.86 (s, 3H, $COCH_3$), 1.67 – 1.53 (m, 1H, CH), 1.44 (tt, *J* = 12.7, 6.0 Hz, 3H, CH_2 + CH), 1.20 (dd, *J* = 10.6, 7.3 Hz, 3H, CH_3).

^{13}C NMR (101 MHz, DMSO-d_6) δ (ppm) = 213.71, 172.17, 166.88, 160.73, 157.11, 150.18, 134.19, 129.08, 128.83, 126.69, 52.99, 51.34, 49.87, 35.03, 29.04, 25.49, 22.87, 15.95.

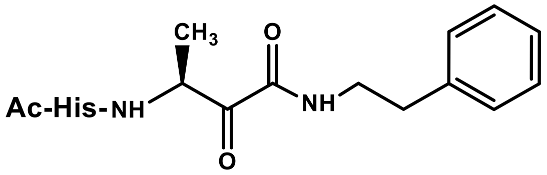

Compound ***14***

LC-MS analysis: One peak at 6.66 min, corresponding to $[M+H]^+$ 400.199 and $[M+H_2O+H]^+$ 418.21.

HR-MS: Monoisotopic mass: calculated 399.190654 for $C_{20}H_{25}N_5O_4$, found $[M+H]^+$ 400.19802 and $[M+Na]^+$ 422.17988.

^{1}H NMR (400 MHz, DMSO-d_6) δ (ppm) = 14.16 (s, 1H, imidazole), 8.96 (s, 1H, aromatic), 8.76 (dt, *J* = 11.7, 6.0 Hz, 1H, CONH), 8.49 – 8.38 (m, 1H, CONH), 8.21 – 8.11 (m, 1H, CONH), 7.38 – 7.25 (m, 3H, aromatic), 7.20 (d, *J* = 8.7 Hz, 3H, aromatic), 5.04 – 4.94 (m, 1H, CH), 4.70 – 4.60 (m, 1H, CH), 3.10 – 2.97 (m, 1H, CH_2), 2.93 – 2.81 (m, 1H, CH_2), 2.77 (t, *J* = 7.7 Hz, 2H, CH_2), 1.82 (d, *J* = 3.7 Hz, 3H, $COCH_3$), 1.19 (dd, *J* = 15.4, 7.3 Hz, 3H, CH_3).

^{13}C NMR (101 MHz, DMSO-d_6) δ (ppm) = 193.52, 169.85, 160.86, 129.07, 128.81, 126.66, 51.35, 49.85, 35.03, 22.99, 15.89.

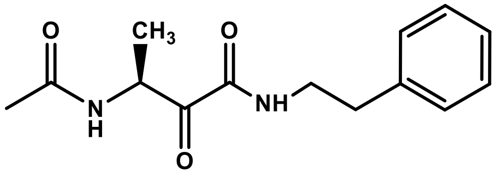

Compound ***15***

LC-MS analysis: One peak at 7.34 min, corresponding to the molecular mass: $[M+H]^+$ 263.14.

HR-MS: Monoisotopic mass: calculated 262.13176 for $C_{14}H_{18}N_2O_3$, found $[M+H]^+$ 236.13902 and $[M+Na]^+$ 285.12096.

^{1}H NMR (401 MHz, DMSO-d_6) δ (ppm) = 8.71 (t, *J* = 5.8 Hz, 1H, COCONH), 8.31 – 8.23 (m, 1H, CONH), 7.34 – 7.14 (m, 5H, aromatic), 4.98 – 4.89 (m, 1H, CH), 2.77 (t, *J* = 7.5 Hz, 3H, aliphatic), 1.82 (s, 3H, $COCH_3$), 1.18 (d, *J* = 7.3 Hz, 3H, CH_3).

^{13}C NMR (101 MHz, DMSO-d_6) δ (ppm) = 197.36, 169.06, 160.60, 139.04, 128.61, 126.17, 49.22, 34.57, 21.97, 15.57.

Supplementary Tables

Table S1, related to Fig. 1 and 7: Crystallographic data collection and refinement statistics

Ligand	Ac-VRHA-cmk	Ac-RVRHA-cmk	Compound ***9***	Compound ***10***
PDB code	5MT7	5MT8	5MT6	5MTF
**Data Collection**				
Cell dimensions				
a, *b*, *c* (Å)	110.9, 110.9, 126.3	110.2, 110.2, 128.9	111.7, 111.7, 124.2	111.3, 111.3, 124.5
γ (°)	120	120	120	120
Resolution (Å)	44.9-2.05 (2.11-2.05)*	44.9-1.95 (2.0-1.95)*	52.3-2.16 (2.23-2.16)*	44.9-1.78 (1.8-1.78)*
R_{merge}	0.036 (0.95)	0.032 (1.0)	0.08 (0.72)	0.045 (0.75)
$I / \sigma I$	28.0 (2.4)	24.5 (1.6)	10.2 (1.9)	18.64 (1.86)
Completeness (%)	99.7 (96.5)	99.6 (95.5)	99.4 (99.6)	99.8 (99.6)
CC1/2	0.99 (0.8)	0.99 (0.59)	0.99 (0.56)	1.0 (0.70)
Redundancy	9.9 (9.4)	6.5 (6.0)	4.7 (4.6)	5.1 (5.0)
Wilson B factor (Å^2)	53.6	48.2	43.9	37.9
**Refinement**				
Resolution (Å)	44.9-2.05	44.9-1.95	52.3-2.16	44.969-1.79
No. of reflections	18930	21617	16096	26791
R_{work} / R_{free}**	0.201/0.224	0.199/0.225	0.213/0.266	0.169/0.192
No. of atoms				
Total	1486	1531	1528	1670
Protein	1431	1426	1431	1480
Ligand***	37	42	58	60
Heteroatoms***	-	34	11	-
Water	18	29	28	68
B-factors (Å^2)				
Total	59.7	53.2	47.5	35.7
Protein	60.3	54.5	47.9	35.1
Ligand	66.3	57.4	53.8	38.1
Heteroatoms	-	71.5	68.5	39.9
Water	58.2	56.2	49.8	41.7
R.m.s.deviations				
Bond lengths (Å)	0.01	0.01	0.01	0.018
Bond angles (°)	1.4	1.3	1.5	1.65

* Values in parentheses are for highest-resolution shell.
** A subset of reflections (5 %) was used for calculation of R_{free} and remaining (95 %) reflections were used for calculation of R_{work}.
*** Heteroatoms denote detergent and ions. Ligand denotes the inhibitor covalently bonded to S201 and or H254.
