# Supplementary material for: General and Modular Strategy for Designing Potent, Selective, and Pharmacologically Compliant Inhibitors of Rhomboid Proteases
Source: Cell Chem Biol. 2017 Dec 21;24(12):1523–1536.e4. doi: 10.1016/j.chembiol.2017.09.007 (PMC5746060; doi:10.1016/j.chembiol.2017.09.007)
Supplement: Document S2. Article plus Supplemental Information [file mmc2.pdf]

Article

Cell Chemical Biology

General and Modular Strategy for Designing Potent, Selective, and Pharmacologically Compliant Inhibitors of Rhomboid Proteases

Graphical Abstract

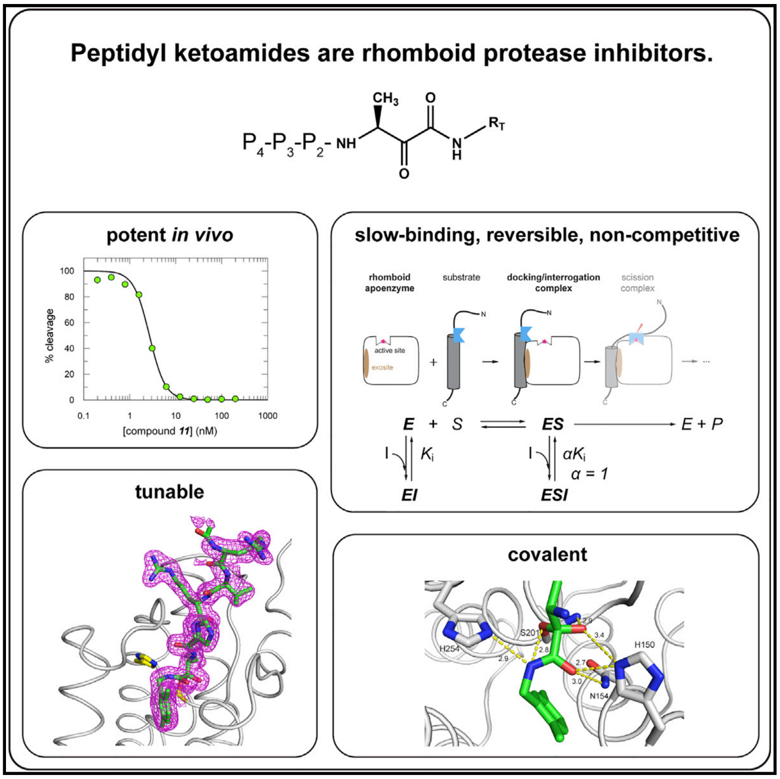

Authors

Anežka Tichá, Stancho Stanchev, Kutti R. Vinothkumar, ..., Martin Lepšík, Pavel Majer, Kvido Strisovsky

Correspondence

kvido.strisovsky@uochb.cas.cz

In Brief

Ticha et al. discover rhomboid protease inhibitors that are unprecedentedly potent and selective. They are based on a pharmacologically compliant chemotype and can thus serve as hitherto unavailable specific tools for cell biology or can yield lead compounds targeting rhomboids in medically relevant contexts such as malaria or Parkinson's disease.

Highlights

- N-substituted peptidyl α-ketoamides are nanomolar inhibitors of rhomboid proteases
- Peptidyl ketoamides inhibit rhomboids covalently, reversibly, and non-competitively
- The peptide and ketoamide substituent independently modulate potency and selectivity
- Peptidyl ketoamides are selective for rhomboids, sparing most human serine proteases

Tichá et al., 2017, Cell Chemical Biology *24*, 1523–1536
December 21, 2017 © 2017 The Authors. Published by Elsevier Ltd.
https://doi.org/10.1016/j.chembiol.2017.09.007

CellPress

Cell Chemical Biology
Article

CellPress

General and Modular Strategy for Designing Potent, Selective, and Pharmacologically Compliant Inhibitors of Rhomboid Proteases

Anežka Tichá,[1,4,9] Stancho Stanchev,[1,9] Kutti R. Vinothkumar,[5] David C. Mikles,[1] Petr Pachl,[1] Jakub Began,[1,3] Jan Škerle,[1,2] Kateřina Švehlová,[1] Minh T.N. Nguyen,[6] Steven H.L. Verhelst,[6,7] Darren C. Johnson,[8] Daniel A. Bachovchin,[8] Martin Lepšík,[1] Pavel Majer,[1] and Kvido Strisovsky[1,10,*]

[1]Institute of Organic Chemistry and Biochemistry, Academy of Sciences of the Czech Republic, Flemingovo n. 2, Prague 166 10, Czech Republic
[2]Department of Biochemistry, Faculty of Science, Charles University, Hlavova 2030/8, Prague 128 43, Czech Republic
[3]Department of Genetics and Microbiology, Faculty of Science, Charles University, Viničná 5, Prague 128 44, Czech Republic
[4]First Faculty of Medicine, Charles University, Kateřinská 32, Prague 121 08, Czech Republic
[5]Medical Research Council Laboratory of Molecular Biology, Cambridge Biomedical Campus, Francis Crick Avenue, Cambridge CB2 0QH, UK
[6]Leibniz Institute for Analytical Sciences ISAS, Otto-Hahn-Strasse 6b, 44227 Dortmund, Germany
[7]KU Leuven - University of Leuven, Herestraat 49, Box 802, 3000 Leuven, Belgium
[8]Chemical Biology Program, Memorial Sloan Kettering Cancer Center, 1275 York Avenue, Box 428, New York, NY 10065, USA
[9]These authors contributed equally
[10]Lead Contact
*Correspondence: kvido.strisovsky@uochb.cas.cz
https://doi.org/10.1016/j.chembiol.2017.09.007

SUMMARY

Rhomboid-family intramembrane proteases regulate important biological processes and have been associated with malaria, cancer, and Parkinson's disease. However, due to the lack of potent, selective, and pharmacologically compliant inhibitors, the wide therapeutic potential of rhomboids is currently untapped. Here, we bridge this gap by discovering that peptidyl α-ketoamides substituted at the ketoamide nitrogen by hydrophobic groups are potent rhomboid inhibitors active in the nanomolar range, surpassing the currently used rhomboid inhibitors by up to three orders of magnitude. Such peptidyl ketoamides show selectivity for rhomboids, leaving most human serine hydrolases unaffected. Crystal structures show that these compounds bind the active site of rhomboid covalently and in a substrate-like manner, and kinetic analysis reveals their reversible, slow-binding, non-competitive mechanism. Since ketoamides are clinically used pharmacophores, our findings uncover a straightforward modular way for the design of specific inhibitors of rhomboid proteases, which can be widely applicable in cell biology and drug discovery.

INTRODUCTION

Rhomboid intramembrane proteases are evolutionarily conserved proteins with numerous important biological functions, including growth factor secretion, regulation of mitochondrial dynamics, and membrane protein quality control (Fleig et al., 2012). As such, they are being increasingly explored as potential drug targets, for example, for malaria (Baker et al., 2006; Lin et al., 2013; O'Donnell et al., 2006), cancer (Song et al., 2015), Parkinson's disease (Meissner et al., 2015), and diabetes (reviewed in Chan and McQuibban, 2013). These efforts are, however, hindered by the lack of selective and potent rhomboid inhibitors that could be used for cell biological studies, validation of therapeutic potential of rhomboids, and as templates for drug development (Strisovsky, 2016a). As explained elsewhere in more detail (Strisovsky, 2016a), the currently used inhibitors of rhomboid proteases suffer from drawbacks, making them unsuitable for these purposes. Isocoumarins are highly reactive and lack selectivity (Harper et al., 1985; Powers et al., 2002; Powers et al., 1989), β-lactams have limited potency *in vivo* (half maximal inhibitory concentration [IC_{50}] ~5–10 μM) (Pierrat et al., 2011), and β-lactones are not very potent (apparent IC_{50} of ~40 μM) (Wolf et al., 2013). Furthermore, no rational strategy for modulation of their selectivity exists for any of these inhibitor classes. Here, we address both of these bottlenecks.

The principles of the mechanism and specificity of a protease determine to a large extent the strategies for inhibitor development (Drag and Salvesen, 2010). Rhomboids are serine proteases with a Ser-His catalytic dyad (Wang et al., 2006), and they recognize their transmembrane substrates in a two-tier process. It is assumed that first a portion of the transmembrane domain of the substrate docks into an intramembrane interaction site of rhomboid within the plane of the lipid bilayer, upon which a linear segment of the substrate (possibly generated by local unfolding of the top of the substrate's transmembrane helix) interacts with the water-exposed active site (reviewed in Strisovsky, 2016a; Strisovsky, 2013). This "recognition motif" encompasses the P4 to P2′ (Schechter and Berger, 1967) residues of the substrate (Strisovsky et al., 2009), it largely determines the k_{cat} of the

This is an open access article under the CC BY license (http://creativecommons.org/licenses/by/4.0/).

reaction (Dickey et al., 2013), and thus modulates selectivity toward a given rhomboid protease (Ticha et al., 2017). Recent reports have shown that peptidyl chloromethylketones (Zoll et al., 2014) and peptidyl aldehydes (Cho et al., 2016) are weakly inhibiting rhomboid proteases at medium to high micromolar concentrations, but they lack selectivity and their potency is insufficient for use as research tools.

Inspired by the current knowledge of the rhomboid protease mechanism (reviewed in Strisovsky, 2016a), we set out to explore the chemical space of oligopeptides equipped with electrophilic warheads in search of new rhomboid inhibitors of greater potency. Our systematic analysis resulted in the discovery of a modular scaffold based on peptidyl-ketoamide substituted with hydrophobic groups that represents a novel class of potent and selective rhomboid inhibitors. The *in vivo* activity of these compounds is in the low nanomolar range, which is up to three orders of magnitude more potent than any other currently known rhomboid inhibitors. Furthermore, we gained insight into the mode of binding of peptidyl ketoamides by solving their co-crystal structures with rhomboid protease, and we present strategies to modify their selectivity and potency on a systematic basis. We expect this compound class to find a widespread use in cell biology in rhomboid protease related contexts and to provide templates for the development of drugs targeting rhomboid proteases.

RESULTS

The Potency of Substrate-Derived Peptidyl Chloromethylketone Inhibitors Can be Markedly Enhanced by Optimizing the Amino Acid Sequence of the P5 to P1 Region

Rhomboid proteases exhibit discernible sequence preferences in the P5 to P2′ region of their substrates (Strisovsky et al., 2009; Zoll et al., 2014). To gain insight into these preferences and their possible interactions, we have generated tetra- and pentapeptidyl chloromethylketones (CMK or cmk henceforth) harboring amino acids preferred in positions P5 to P1 by the *Escherichia coli* rhomboid GlpG (GlpG henceforth), using the sequence background of the *Providencia* TatA (Stevenson et al., 2007), represented by the parent compound Ac-IATA-cmk. We measured the inhibitory properties of this series of compounds using a newly developed *in vitro* assay employing a fluorogenic transmembrane peptide substrate (Ticha et al., 2017) that faithfully represents a native rhomboid substrate. The effects of the mutations were additive, and the inhibitor containing the most favored amino acids in positions P5 to P1 (Ac-RVRHA-cmk) is approximately 26-fold more potent than the parent compound Ac-IATA-cmk (Figure 1A).

To provide mechanistic explanation for the observed increase in inhibitory potency, we determined the structures of GlpG in complex with Ac-RVRHA-cmk and Ac-VRHA-cmk (Figure 1B). The side chain of Arg in the P5 position of Ac-RVRHA-cmk could not be modeled due to poor electron density, and the two structures are otherwise virtually identical; superposition of all corresponding Cα atoms yields a root-mean-square deviation of 0.19 Å per atom (using the SSM method as implemented in CCP4MG v2.10.4; Krissinel and Henrick, 2004; Mitchell et al., 1990). Both inhibitors interact with the L3 and L5 loops via main-chain hydrogen bonds, and via hydrogen bonds involving the side chains of the strongly preferred Arg and His in the P3 and P2 position, respectively (Figure 1C). These interactions are not observed in the structure of the parent compound Ac-IATA-cmk (Zoll et al., 2014), suggesting that they contribute to the higher potency of Ac-RVRHA-cmk and Ac-VRHA-cmk over Ac-IATA-cmk. The interactions of the residues in the P4, P3, and P2 positions with the enzyme are structurally independent, explaining why the effects of substitutions in these positions are additive (Figure 1A). The overall binding mode of both compounds into the rhomboid active site is similar to the binding mode of peptide aldehyde Ac-VRMA-cho (Cho et al., 2016) (Figure 1D). Collectively, these data show that rhomboid subsite preferences are additive in the context of an active site targeted inhibitor and that sequence optimization in this region can significantly increase the inhibitory potency of the compounds.

A Screen of Covalent Reversible Warheads for Inhibition of Rhomboid

Since the sequence-optimized chloromethylketones are poor inhibitors with low micromolar IC_{50}, we searched for alternative, more suitable electrophilic warheads that might improve the inhibitory potency. Furthermore, we reasoned that extending the inhibitor to the prime side of the active site might offer additional binding energy. We therefore synthesized a series of compounds based on the Ac-RVRHA sequence equipped with a selection of electrophilic, reversibly binding warheads commonly used for serine proteases in pharmacological settings (reviewed in Hedstrom, 2002; Walker and Lynas, 2001), including trifluoromethylketones, boronates, acylsulfonamides, thiazolylketones, and ketoamides (Figure 2), the last three of which can be extended into the prime side. We measured the apparent IC_{50} values of these compounds, and while trifluoromethylketones, acylsulfonamides, and thiazolylketones showed none or very weak inhibition in the millimolar range, the apparent IC_{50} of the boronate was 8 μM and of the ketoamide 203 μM under identical reaction conditions (Figure 2). Although the peptidyl boronate was the best of the series, it was still a relatively weak inhibitor comparable with the parent chloromethylketone, and it was not clear how to further improve its potency. The ketoamide was about 25-fold less potent, but since it could be extended to the prime side by a modification at the ketoamide nitrogen (Chatterjee et al., 1999; Liu et al., 2004), we next focused our attention on this class of compounds.

Extensions at the Prime Side of Peptidyl Ketoamides Greatly Enhance Their Inhibitory Potency

We hypothesized that extending the peptidyl ketoamides to the prime side of the active site might increase their potency, since the P2′ residue (hydrophobic in case of GlpG) was shown to be important for substrate recognition by rhomboids (Dickey et al., 2013; Strisovsky et al., 2009), and interactions of the substrate transmembrane domain beyond P2′ potentiate substrate cleavage in a detergent micelle assay (Ticha et al., 2017). We synthesized a series of peptidyl ketoamides based on the Ac-RVRHA sequence, bearing a mostly hydrophobic "tail" of increasing size at the ketoamide nitrogen (Figure 3A) that could reach far into the prime side of the rhomboid active site. The tail substituent indeed had a dramatic effect on the potency of

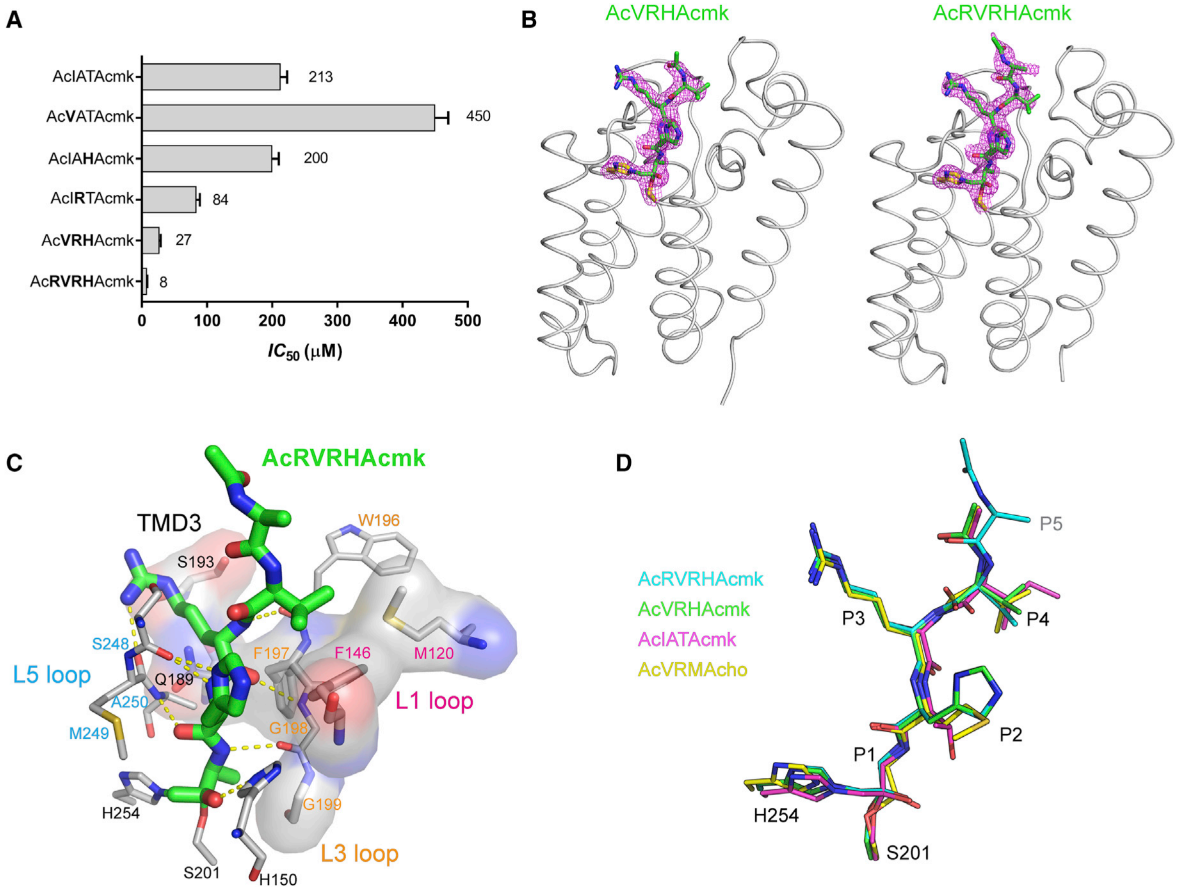

Figure 1. The Potency of Substrate-Derived Inhibitors Can be Improved by Modifying the Amino acid Sequence of the P5 to P1 Region

(A) The parent inhibitor Ac-IATA-cmk was modified by introducing strongly preferred amino acids (Zoll et al., 2014) into the P4, P3, P2, and P5 positions to yield the listed compounds. Their apparent IC_{50} values were measured with 1 hr preincubation using 10 μM fluorogenic substrate KSp35 and 0.05% (w/v) DDM. The reported values are best-fit means with SD representative of 2–3 measurements.

(B) The sequence-optimized peptidyl chloromethylketones were soaked into the native crystals of GlpG and structures of the complexes were solved by X-ray diffraction (for statistics, see Table S1). In the displayed structures, the catalytic dyad is shown as yellow sticks and the inhibitors are shown as green sticks surrounded by the $2mF_o - DF_c$ electron density map contoured at 1σ and shown 1.6 Å around the stick model. Note that in the Ac-RVRHA-cmk structure (right), the side chain of the Arg residue in the P5 position of the inhibitor has not been modeled due to poor or missing electron density peaks.

(C) Interactions of RVRHA-cmk with GlpG were analyzed by Ligplot+ (Laskowski and Swindells, 2011). Ligands are shown as thick sticks with carbons in green, proteins as thin sticks with carbons in gray, hydrogen bonds as yellow dashed lines, and amino acids involved in van der Waals contacts are highlighted as transparent surfaces. The inhibitor forms covalent bonds with S201 and H254 via the chloromethylketone warhead, and it hydrogen bonds with the backbone of residues 196–198 from the L3 loop and residues 248–250 from the L5 loop. van der Waals contacts with the inhibitor are formed by the residues from the L3 loop of GlpG, by S193 and Q189 from TMD3, and by F146 and M120 from the L1 loop that pack against the Val side chain of the P4 position of the inhibitor, as observed previously (Zoll et al., 2014).

(D) The conformations of peptide inhibitors bound to the active site of GlpG were compared by performing structural alignment of the complexes of VRHAcmk (PDB: 5MT7), RVRHAcmk (PDB: 5MT8), IATAcmk (PDB: 4QO2), and VRMAcho (PDB: 5F5B) in PyMOL (Schrodinger, 2012). Note that the structure of RVRHAcmk suggests where the P5 amino acid points, but the density for this side chain is not visible beyond its β-carbon. The backbone of the ligands in all complexes has virtually identical conformation with the exception of the distortion of the oxyanion by the chloromethylketone, and the biggest differences are found in the conformation of the P2 side chain, which is not surprising, because almost any side chain can be accommodated in this position (Zoll et al., 2014).

the inhibitors *in vitro* (Figure 3A). The most effective compound of the series, bearing a 4-phenyl-butyl tail (compound **11**), already displayed about 1,000-fold lower IC_{50} than the parent compound **1**. The IC_{50} of **11** reaches half of the enzyme concentration used in the assay, suggesting that **11** is a potent inhibitor of GlpG.

Next, we examined the relative importance of the peptidyl part for the inhibitory potency. We generated a series of progressively N-terminally truncated variants of **9** and measured their inhibitory potency against GlpG (Figure 3B). Removing the P5 Arg from **9** to yield **12** had virtually no effect on IC_{50} (0.44 versus 0.55 μM), while removing the P5 and P4 residues in **13** led to a ~20-fold decrease in potency in comparison with the parent compound **9** (IC_{50} changes from 0.44 to 9 μM). Removing three residues (from P5 to P3) in **14** led to a dramatic ~150-fold loss of potency, yielding a weak inhibitor with about 65 μM IC_{50}, and the absence of the P5 to P2 residues in **15** resulted in a total ~2,250-fold reduction in potency compared with **9** and IC_{50} higher than 1 mM. This experiment demonstrates that the non-prime (P4 to P1) and prime sides of the inhibitor contribute to its potency almost equally. The P5 residue can be omitted with only a

Ac-Arg-Val-Arg-His-Ala-X

warhead	Ala-X	IC_{50}
chloromethylketone	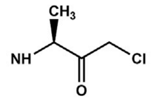	8 μM
trifluoromethylketone	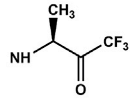	> 1 mM
boronate	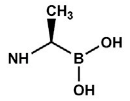	8 μM
acylsulfonamide	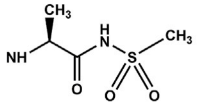	> 1 mM
thiazolylketone	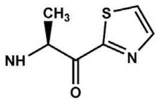	> 1 mM
α-ketoamide	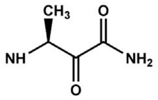	203 μM

Figure 2. A Screen of Electrophilic Warheads for the Inhibition of Rhomboid Proteases

The optimized parent sequence Ac-RVRHA was linked to electrophilic warheads commonly used for targeting serine proteases (reviewed in Hedstrom, 2002; Walker and Lynas, 2001). The apparent IC_{50} values of the compounds were measured in 0.05% DDM using 10 μM substrate KSp35 (Ticha et al., 2017) with 1 hr preincubation. Given are the mean values of 2–3 measurements.

marginal effect on inhibitory potency, which can be probably compensated by a suitable prime side tail substituent.

Ketoamides are known to be covalent reversible inhibitors of soluble serine proteases with a classical catalytic triad (Liu et al., 2004). Since rhomboids are unusual serine proteases using only a Ser-His dyad for catalysis (Wang et al., 2006), we investigated the mechanism of rhomboid inhibition by these compounds more closely. Progress curves measured at varying inhibitor concentrations (Figure 4A) had biphasic character; especially at the highest inhibitor concentrations tested, the reaction rate decreased over approximately the first hour and became more or less constant over the next hour (Figures 4A and 4B). This indicates that inhibition was time dependent, which is typical for slow-binding inhibitors (Copeland, 2013b). In addition, upon rapid dilution of inhibitor-saturated enzyme to a subinhibitory concentration, the reaction rate was partially recovered (Figure 4C), together indicating that peptidyl ketoamides exhibit slow-binding reversible behavior (Copeland, 2013a; Singh et al., 2011).

The slow-binding reversible inhibition mechanism can be formally divided into two steps. First, an initial encounter complex (EI) forms, and then a slow step leads to the much more stable EI* complex (E + I ↔ EI ↔ EI*), usually involving a significant conformational change of the enzyme (Copeland, 2013a). To analyze the contribution of each of these two steps to the mechanism of inhibition of rhomboids by peptidyl ketoamides, we investigated the concentration and time dependence of inhibition kinetics by **10**. The "bending" of biphasic progress curves (Figures 4A and 4B) reflects the rate of "onset of inhibition" described by the rate constant k_{obs}, which can be obtained from progress curve data using non-linear fitting to Equation 1:

$$[P] = v_s t + \frac{(v_i - v_s)}{k_{obs}}[1 - \exp(-k_{obs}t)], \qquad \text{(Equation 1)}$$

where $[P]$ is the concentration of the reaction products, v_i is the initial reaction rate in the first phase of the biphasic progress curve, and v_s is the steady-state reaction rate (Figure 4B). Analysis of progress curves from Figure 4A showed that v_i was independent of inhibitor concentration, and the plot of k_{obs} against inhibitor concentration fitted well to a linear dependence (Figure 4D). Both phenomena are typical for simple (single-step) slow-binding inhibition (E + I ↔ EI) (Figure 4D); in other words, peptidyl ketoamides behave as "regular" reversible inhibitors but with very low rate constants for association and dissociation (Copeland, 2013a; Morrison, 1982), leading to the slow-binding kinetics. The application of this model yields the apparent inhibitory constant K_i^{app} (i.e., not taking into account the inhibition modality and the influence of the substrate) for **10** of (123 ± 47) nM (Figure 4D).

The true inhibitory constant K_i, which is an important, substrate-independent property of an inhibitor, can be calculated from the apparent inhibitory constant K_i^{app}, depending on the inhibitory modality and kinetic parameters of the substrate used. Global non-linear regression fitting of Michaelis curves measured in the presence of increasing concentrations of **10** (plotting v_s against [S]) shows that the experimental data are best described by a non-competitive inhibition model (Figure 4E). This inhibition mode means that the inhibitor can bind both to the free enzyme and to the enzyme-substrate complex; in this case specifically, the affinities of the inhibitor to both forms of the enzyme are equal (α = 1) (Copeland, 2013a). Although non-competitive modality is non-typical for slow-binding inhibitors, it is conceivable why it is plausible in the case of peptidyl ketoamides and rhomboids. Several studies have suggested that substrate recognition by rhomboid proteases proceeds in two steps, via a docking/interrogation complex, where only a part of substrate's transmembrane domain interacts with rhomboid, followed by the interaction of the recognition motif with the active site forming the scission-competent complex (Cho et al., 2016; Strisovsky, 2016a, 2016b; Strisovsky et al., 2009) (Figure 4E). Since the active site is unoccupied in the docking complex, binding of an active site-directed inhibitor is possible (Figure 4E), resulting in non-competitive behavior. Under this mechanism of inhibition, the true K_i is identical to K_i^{app} (Copeland, 2013a; Purich, 2010). Similar progress curve analyses of **9** and **11** yield their k_{on} and k_{off} rate constants, their K_i^{app} ($K_i^{app} = k_{off}/k_{on}$), and the true K_i values of (219 ± 76) nM and (45 ± 8) nM, respectively (Figure 4F and Table 1). In summary, this kinetic analysis shows that the peptidyl ketoamides described here are high-affinity inhibitors of rhomboid proteases unprecedented in the literature.

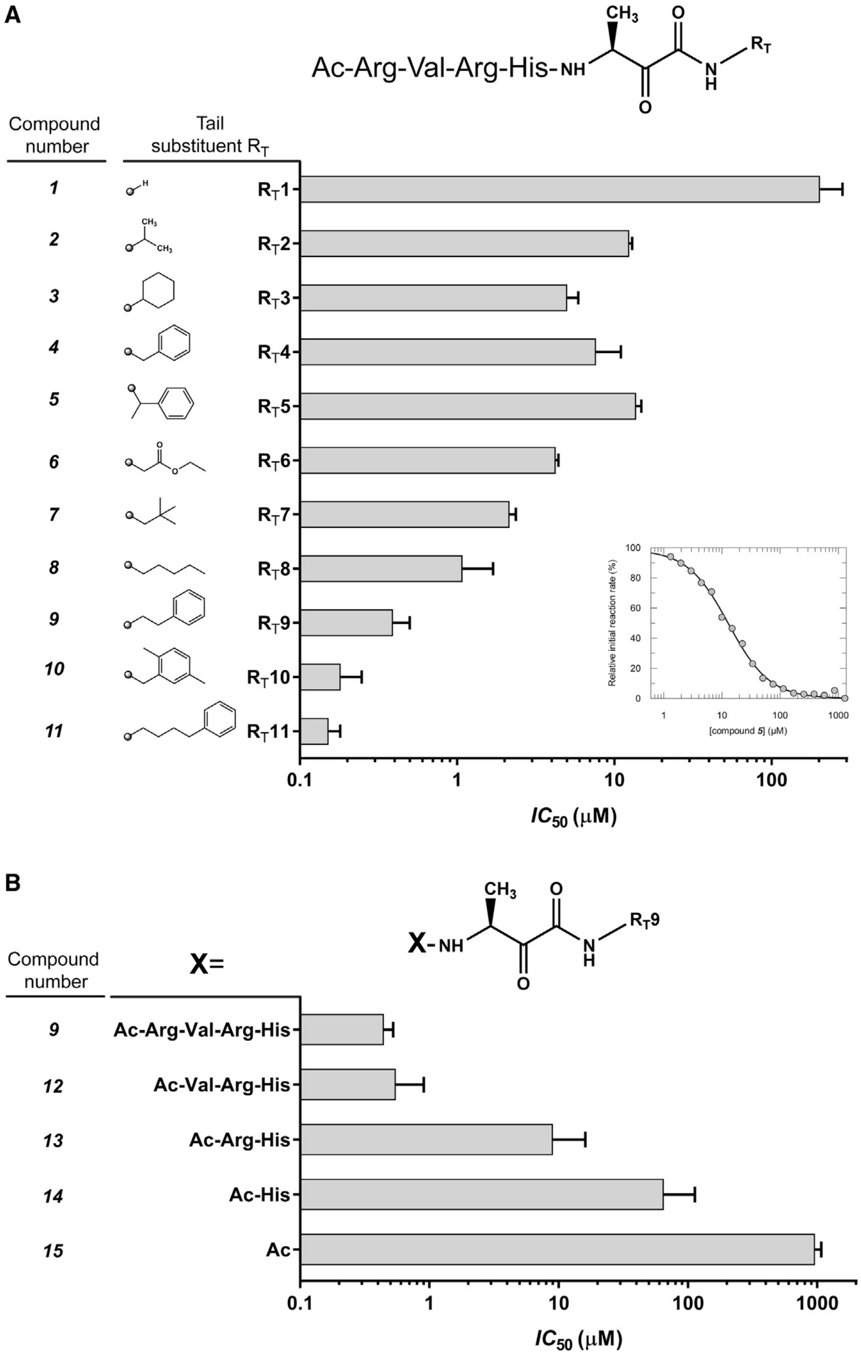

Figure 3. Modification of the Prime-Side Substituent at the Amide Group of Peptidyl Ketoamides Enhances Their Potency by Orders of Magnitude

(A) A screen of the effect of the tail substituent RT on the inhibitory properties of ketoamide inhibitors of GlpG based on the parent compound Ac-RVRHA-$CONH_2$. The apparent IC_{50} values of all compounds were measured in 0.05% (w/v) DDM and 10 μM KSp35 (Ticha et al., 2017) with 1 hr preincubation. The IC_{50} values of the most effective compounds **9**, **10**, and **11** are three orders of magnitude lower than that of the parent compound **1**. The reported values are best-fit means with SD representative of 2–3 measurements. The inset shows a typical inhibition curve.

(B) The significance of the peptidyl part in compound **9**. The peptidyl part of **9** was progressively truncated from the N terminus, and the apparent IC_{50} values of all compounds were measured in 0.05% (w/v) DDM and 10 μM KSp35 (Ticha et al., 2017) with 1 hr preincubation. The reported values are best-fit means with SD representative of 2–3 measurements.

Selectivity of Peptidyl Ketoamides

Any enzyme inhibitors to be used as specific tools for cell biology or as starting points for drug development must show sufficient level of selectivity toward their intended target. This is particularly important for compounds that react with the catalytic nucleophile common to many serine hydrolases. Only limited tests of selectivity have been conducted for the currently used rhomboid inhibitors isocoumarins, β-lactams and β-lactones, at best interrogating them against trypsin or chymotrypsin (Pierrat et al., 2011; Vosyka et al., 2013). To map the selectivity of peptidyl ketoamides more objectively and widely, we employed activity-based probe (ABP) competition assays that enable a more general and substrate-independent measurement of inhibitory potency, because they rely solely on the competition between a fluorescently labeled activity-based probe and the tested inhibitor (Nguyen et al., 2015; Serim et al., 2012). The assays we employed used fluorophosphonate ABPs that target the catalytic serine of a wide-range of serine hydrolases (Bachovchin et al., 2014), including rhomboids (Xue et al., 2012), and are thus very practical general detection reagents even for serine hydrolases for which sensitive substrates might not be available.

First, we tested **9**, **10**, and **11** against a panel of bacterial and eukaryotic rhomboid proteases (Wolf et al., 2015), and found that all three compounds potently competed with ABP labeling of rhomboids from bacterium *Providencia stuartii* (AarA), archaebacterium *Methanocaldococcus jannaschii* (MjROM), and three closely related rhomboids from bacteria *E. coli* (EcGlpG), *Haemophilus influenzae* (HiGlpG), and *Vibrio cholerae* (VcROM). Compounds **9**, **10**, and **11** outcompeted the ABP even at a concentration of 500 nM, suggesting that they were potent inhibitors of these rhomboid proteases. In contrast, none of these compounds were able to compete with the ABP labeling of rhomboid protease from the bacterium *Aquifex aeolicus* (AaROM), rhomboids from *Drosophila* (DmRho1) and mouse (MmRHBDL3), and they only partially inhibited labeling of rhomboid protease from bacterium *Thermotoga maritima* (TmROM) at 50 μM (Figure 5A). These data demonstrate that already these first-generation

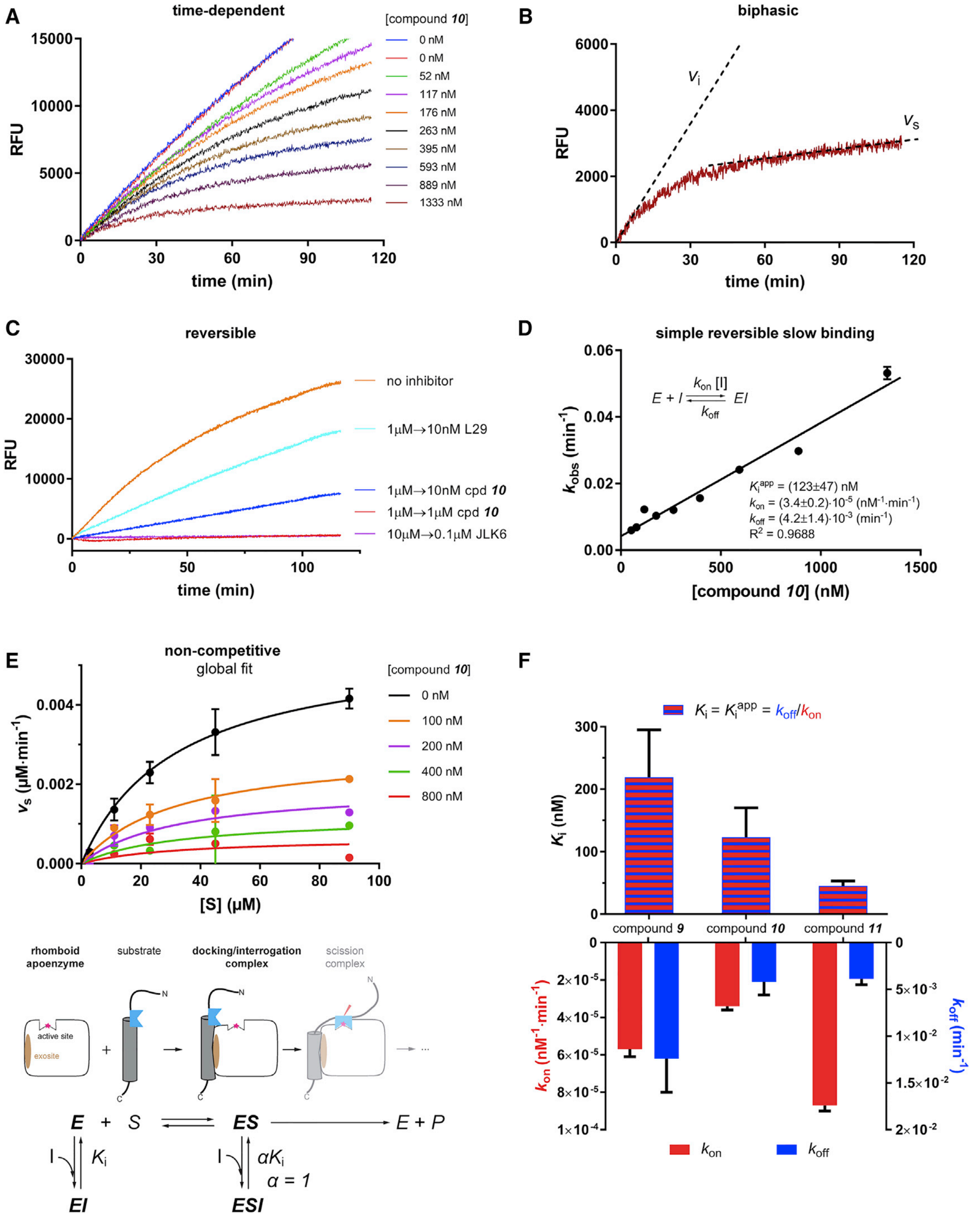

Figure 4. Mechanism of Inhibition of Rhomboid Protease GlpG by Peptidyl Ketoamides Analyzed Using Fluorogenic Transmembrane Peptide Substrates

(A) Progress curves in the presence of increasing concentrations of compound **10** show biphasic character, which is typical for slow-binding inhibitors (Copeland, 2013a; Morrison, 1982). GlpG (0.5 nM) was incubated with 25 μM substrate KSp93 in the presence of 0.05% (w/v) DDM and 0–1,333 nM **10**. Fluorescence at 493 nm was followed to monitor substrate cleavage.

(B) Biphasic progress curves characterized by an initial reaction rate (v_i) and steady state reaction rate (v_S). The progress curve at 1,333 nM compound **10** from the experiment in (A) is shown in detail, and both reaction rates obtained from non-linear regression into Equation (1) are shown as dotted lines.

(C) Reversibility of inhibition by ketoamides was assessed by the rapid dilution method (Harper et al., 1985; Harper and Powers, 1985). Compound **10** (1 μM) was pre-incubated with 0.4 μM GlpG, 0.05% (w/v) DDM at 37°C for 1 hr, leading to complete inhibition. This solution was then rapidly diluted 100-fold either into the reaction buffer containing 10 μM substrate KSp64 (Ticha et al., 2017) (yielding final 10 nM inhibitor) or into the reaction buffer with 10 μM substrate KSp64 and 1,000 nM **10**. For comparison, we used β-lactam L29 (Pierrat et al., 2011) at 1 μM and isocoumarin JLK6 (Vinothkumar et al., 2010) at 10 μM as known reversible

(legend continued on next page)

Table 1. Summary of the Inhibition Properties of Compounds 9–11

	GlpG				YqgP
Compound	K_i (nM)	k_{on} (10^{-6} $nM^{-1}\cdot min^{-1}$)	k_{off} (10^{-3} min^{-1})	IC_{50} *In Vivo* (nM)	IC_{50} *In Vivo* (nM)
**9**	220 ± 80	5.7 ± 0.4	12.0 ± 0.4	8.8 ± 0.4	ND
**10**	120 ± 50	3.4 ± 0.2	4.2 ± 1.4	6.0 ± 0.1	ND
**11**	45 ± 8	8.7 ± 0.3	3.9 ± 0.6	2.7 ± 0.1	~5–10

Values for GlpG are reported as means ± SD.

peptidyl ketoamides can discriminate between diverse rhomboid proteases.

We next examined peptidyl ketoamides for their possible off-target effects on other serine proteases. To get a representative picture of the selectivity of peptidyl ketoamides, we employed a recently developed EnPlex technology, which allows multiplex analysis of ABP competition with about 100 human serine hydrolases, mostly proteases (Bachovchin et al., 2014). Profiling of **9**, **10**, and **11** showed that in the concentration range where they inhibit rhomboid proteases, they fail to inhibit most of the tested human serine hydrolases with the exception of prolylcarboxypeptidase (PRCP) and the sequence related dipeptidyl-peptidase 7 (DPP7) (Figure 5B). To put this into the context of the current generation of rhomboid inhibitors, isocoumarins S006 and S016 (Vosyka et al., 2013) hit about a dozen serine hydrolases in the same concentration range. The β-lactam L41 (Pierrat et al., 2011) inhibited appreciably only one enzyme (predicted serine carboxypeptidase CPVL), but it is much less potent on rhomboids than **9**, **10**, and **11**, and it does not inhibit GlpG completely *in vivo* (Pierrat et al., 2011). The selectivity profile of ketoamide inhibitors of rhomboids is similar to the profile of clinically used ketoamide inhibitors of the hepatitis C protease (Bachovchin et al., 2014), indicating that the rhomboid-targeting N-modified peptidyl ketoamides are sufficiently selective with minimal risk of cross-reactivity against other serine proteases.

Peptidyl Ketoamides Potently Inhibit Rhomboids in Living Cells

Having established the mechanism of rhomboid inhibition by peptidyl ketoamides in detergent micelles, and having shown that **9**, **10**, and **11** are able to inhibit potently rhomboid proteases from several Gram-negative bacteria (Figure 5A), we next tested whether the inhibitors will be able to target rhomboid proteases embedded in their native lipid bilayer in live cells. First, we expressed the model substrate derived from LacYTM2 in *E. coli* expressing endogenous levels of GlpG, incubated the bacterial cultures in the presence of increasing concentrations of **9**, **10**, and **11**, and detected the steady-state levels of substrate processing by quantitative near-infrared western blotting (Figure 6A). The calculated substrate conversion values relative to the uninhibited reaction were plotted against the inhibitor concentration yielding the *in vivo* IC_{50} values. Strikingly, the most effective compound **11** had an *in vivo* IC_{50} value of 2.7 nM, which is three orders of magnitude lower than any other currently known rhomboid inhibitors (Cho et al., 2016; Pierrat et al., 2011).

We then extended the range of organisms to *Bacillus subtilis*, a representative of Gram-positive bacteria, which have a thick cell wall and include major pathogens such as *Staphylococcus*, *Listeria*, *Streptococcus*, and others. Since the endogenous substrate of the *B. subtilis* rhomboid protease YqgP is unknown, and no robust and rescuable phenotypes have been reported for YqgP, we focused on inhibition of cleavage of a model substrate. Of the common model rhomboid substrates, YqgP cleaves LacYTM2 reasonably well (Ticha et al., 2017). We have thus expressed MBP-LacYTM2-Trx (Strisovsky et al., 2009) from the ectopic *xkdE* locus (Gerwig et al., 2014) in the wild-type *B. subtilis* 168 (BS87) and its *yqgP* deletion mutant (BS88) on an otherwise rhomboid-free background. Although the substrate was to some extent truncated by unknown processes in the *ΔyqgP* strain, a specific, closely co-migrating rhomboid-generated N-terminal cleavage product (Figure 6B) was produced in the YqgP wild-type strain BS87 but not in the *ΔyqgP*

and irreversible inhibitors of rhomboid proteases, respectively. Activity recovery was followed by measuring fluorescence over the course of 120 min with excitation at 553 nm and emission at 583 nm.

(D) Progress curves of KSp93 cleavage at increasing concentrations of **10** measured under (A) were analyzed by non-linear regression as described for slow-binding inhibition (Copeland, 2013a; Morrison, 1982) using GraphPad Prism version 7.02 for Windows (GraphPad Software, La Jolla, California, USA) to yield the rate constant for the onset of inhibition, k_{obs}. The linear character of the dependence of k_{obs} on inhibitor concentration is typical for a simple slow-binding mechanism (inset), and its linear regression allows determination of the underlying apparent inhibitory constant K_i^{app} and its constituent rate constants k_{on} and k_{off} (inset). The k_{obs} values are reported as best-fit mean ± SD.

(E) The influence of inhibitor concentration on the apparent K_M and k_{cat} suggests the mode of inhibition by compound **10**. Michaelis curves at the indicated inhibitor concentrations were measured by plotting v_S (measured as in Figure 4B) against substrate concentration using 1 nM GlpG, 0.15% (w/v) DDM and highly sensitive substrate KSp96. The data were globally fitted to the models of competitive, non-competitive (figure top), uncompetitive, and mixed inhibition as implemented in GraphPad Prism 7.02, and their statistical analysis yielded the non-competitive mechanism (figure bottom) as the best fit. The middle of the figure shows a schematic mapping of this mechanism onto the consensual model of substrate recognition by rhomboid proteases. The data points in the Michaelis plots (figure top) represent means ± SD of duplicate measurements.

(F) Summary of inhibition kinetics parameters of compounds **9**, **10**, and **11**. The apparent inhibitory constants K_i^{app} (blue-red striped columns) and the constituent rate constants k_{on} (red columns) and k_{off} (blue columns) were determined from progress curve analysis as shown in (A and C) (note that $K_i^{app} = k_{off}/k_{on}$). For non-competitive inhibitors, the true inhibitory constant K_i equals K_i^{app}. Note that **11** is a highly potent inhibitor with K_i of (45 ± 8) nM. Graphs show best-fit means with SDs.

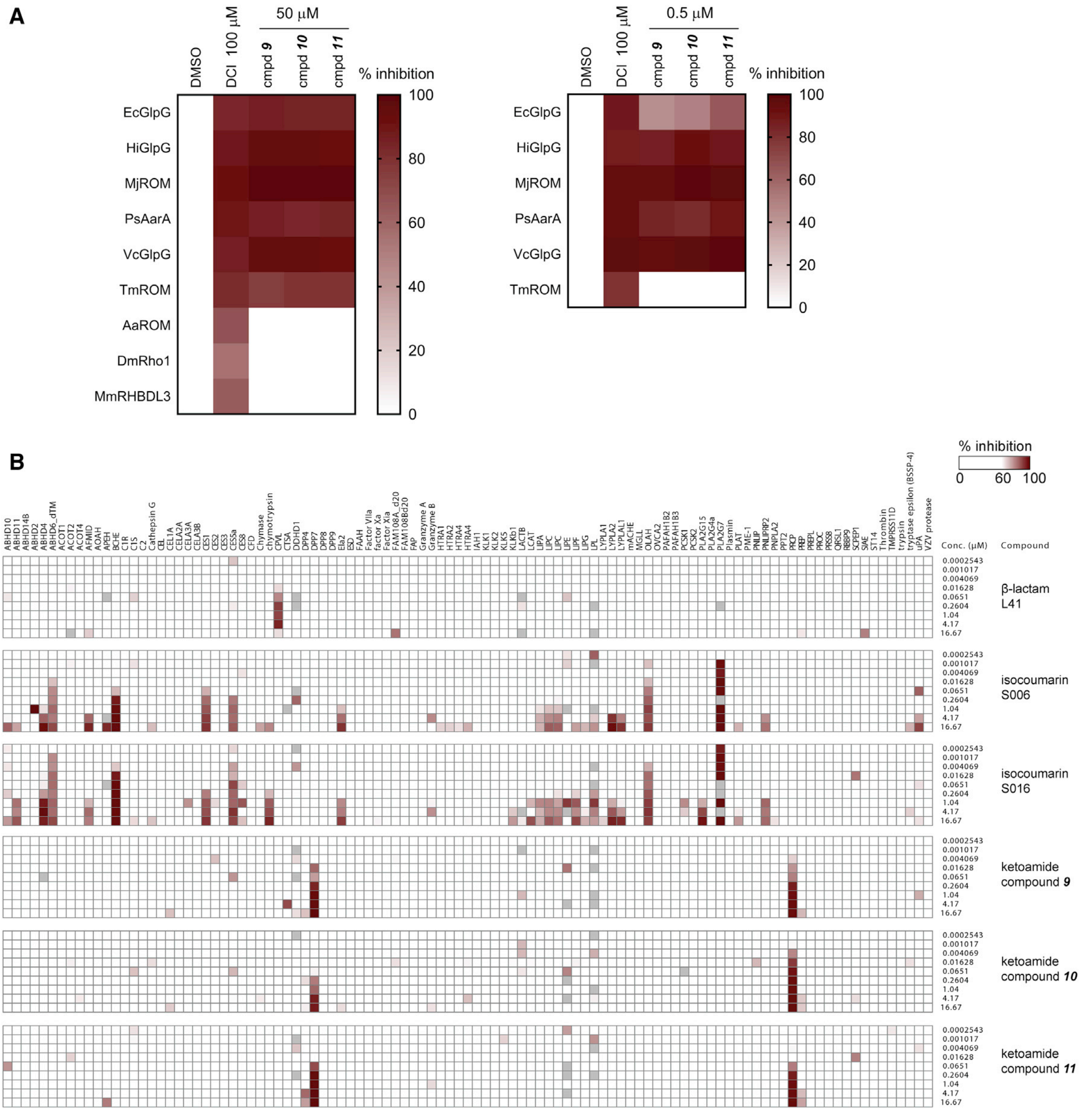

Figure 5. Selectivity of Peptidyl Ketoamides

(A) Selectivity of compounds **9**, **10**, and **11** for nine rhomboid proteases was profiled using activity-based probe competition assay at 50 μM and 0.5 μM concentration. The upper limit of enzyme concentration was 0.4 μM.

(B) Selectivity of compounds **9**, **10**, and **11** against human serine hydrolases was analyzed using EnPlex as described (Bachovchin et al., 2014).

strain BS88 (Figure 6B). In the absence of any inhibitors, MBP-LacYTM2-Trx was cleaved to about 75% conversion by the endogenous YqgP, and addition of **11** into the growth media completely inhibited substrate cleavage at 50 nM (Figure 6B), indicating that the compound can penetrate the Gram-positive cell wall easily. Moreover, since compound **11** also inhibits several homologs of GlpG (Figure 5A), it is safe to assume that YqgP orthologs in other *Bacilli*, *Lactobacilli*, *Staphylococci*, and *Listeria* might be equally susceptible to inhibition by the described inhibitors, and compound **11** and its analogs can be directly used for chemical proteomics and cell biological studies of rhomboid proteases in Gram-positive bacteria.

N-Modified Peptidyl Ketoamides Bind the Rhomboid Active Site in a Substrate-like Manner Occupying the S4 to S2′ Subsites

To understand why peptidyl ketoamides are such efficient rhomboid inhibitors and to establish the basis for structure-guided design of their improved variants, we determined the co-crystal structures of GlpG with **9** and **10** (Figure 7A). The complexes were formed by soaking the inhibitors into apoenzyme crystals, and the structures were solved using diffraction data to 2.16 and 1.78 Å resolution, respectively, allowing detailed comparison of their binding modes. In both cases, the pentapeptide RVRHA binds the active site cavity as an extended β strand,

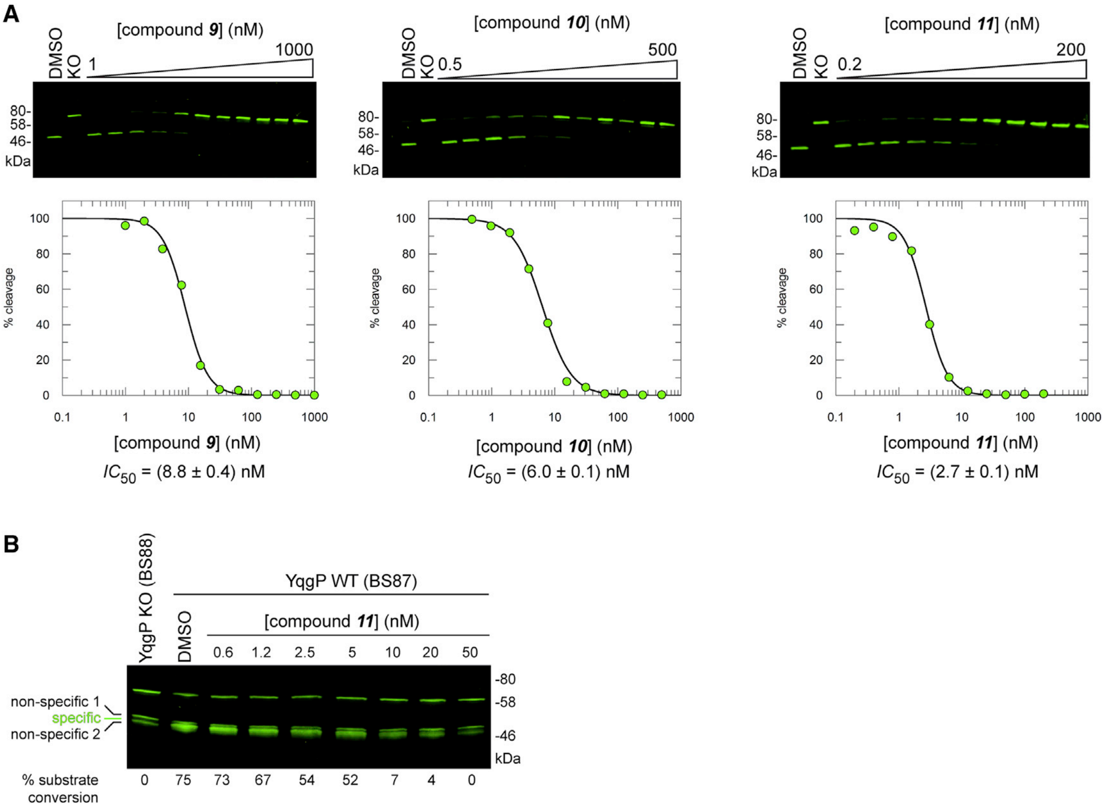

Figure 6. Peptidyl Ketoamides Potently Inhibit Rhomboid Activity in the Membranes of Living Cells

(A) Inhibition of endogenous GlpG by compounds **9**, **10**, and **11** in the membranes of live *E. coli.* The substrate MBP-FLAG-LacYTM2-Trx (Strisovsky et al., 2009) was expressed in wild-type *E. coli* NR698 with genetically permeabilized outer membrane (Ruiz et al., 2005) in the presence of increasing concentrations of inhibitors as described in STAR Methods. Substrate cleavage was measured in cell lysates by immunoblotting for FLAG and quantified using near-infrared fluorescence. The reported *in vivo* IC_{50} values are best-fit means with SD representative of 2–3 measurements. DMSO, dimethylsulfoxide vehicle control; KO, *E. coli glpG::tet.*

(B) Inhibition of endogenous YqgP by compound **11** in the membranes of live *B. subtilis.* The substrate $AmyE_{SP}$-MBP-FLAG-LacYTM2-Trx-HA was expressed in *Bacillus subtilis* 168 (*ydcA::neo*, *xdkE::AmyE$_{SP}$-MBP-LacYTM2-Trx(erm, lin)*) (BS87) in the presence of increasing concentrations of inhibitors as described in STAR Methods. Substrate cleavage was detected in cell lysates by immunoblotting for FLAG and detection by near-infrared fluorescence. Unspecific cleavage of the substrate was corrected for by substracting the intensity of the unspecific bands formed in the YqgP knockout control cells (BS88) from the product band and the closely co-migrating unspecific bands observed in the YqgP positive cells (BS87). This treatment was necessary because the specific cleavage product could not be resolved sufficiently well from the non-specific bands to be integrated separately. DMSO, dimethylsulfoxide vehicle control; YqgP KO, *Bacillus subtilis* 168 (*ydcA::neo*, *yqgP::tet, xdkE::AmyE$_{SP}$-MBP-LacYTM2-Trx(erm, lin)*) (BS88).

virtually identically to the binding mode of Ac-RVRHA-cmk (Figure 1C). We do observe electron density for the side chain of arginine in the P5 position in both structures, but its conformation differs between **9** and **10** (Figure 7A), and it is influenced by crystal contacts with the same residue from a neighboring molecule in the crystal (data not shown).

In both inhibitors, the ketoamide warhead is covalently bonded via its proximal carbon to the side-chain oxygen of the catalytic S201, and it engages in a network of six hydrogen bonds in the active site (Figure 7B). The oxyanion formed by the proximal carbonyl oxygen accepts hydrogen bonds from His150 and the main-chain amide nitrogen of the catalytic serine, and the distal ketoamide carbonyl oxygen accepts hydrogen bonds from both H150 and N154, thus amply saturating the hydrogen-bonding groups engaged in the stabilization of the oxyanion (Cho et al., 2016). Furthermore, the ketoamide nitrogen donates a hydrogen bond to H254 and to the S201 side-chain oxygen covalently bound to the warhead. The resulting network of six hydrogen bonds (Figure 7B) probably helps position the ketoamide warhead in the proximity of the hydroxyl of the catalytic S201 to enhance its chemical reactivity in a conformation-dependent manner.

The tail substituents of **9** and **10** (R_T9 and R_T10) interact with the prime side of GlpG, buried in a cavity delimited by the side chains of amino acids F245, M247, and M249 from the L5 loop, W236 from TMD5, F153 and W157 from TMD2, and residues V204, M208, Y205, H254, H150, and N154 (Figure 7C). The different sets of residues making van der Waals contacts with each ketoamide tail are shown in magenta. The NH group of the side chain of W236 seems to form a weak H-π bond with the phenyl ring of the tail of both compounds, and F245 engages in $\pi \ldots \pi$ stacking against the dimethylbenzyl in R_T10 (Figure 7C). Structural alignment of both ketoamide complexes to the complex of the β-lactam L29 (Vinothkumar et al., 2013) and isocoumarin S016 (Vosyka et al., 2013) (Figure 7D) shows that the tails of **9** and **10** bind in a similar area (the S2′ subsite)

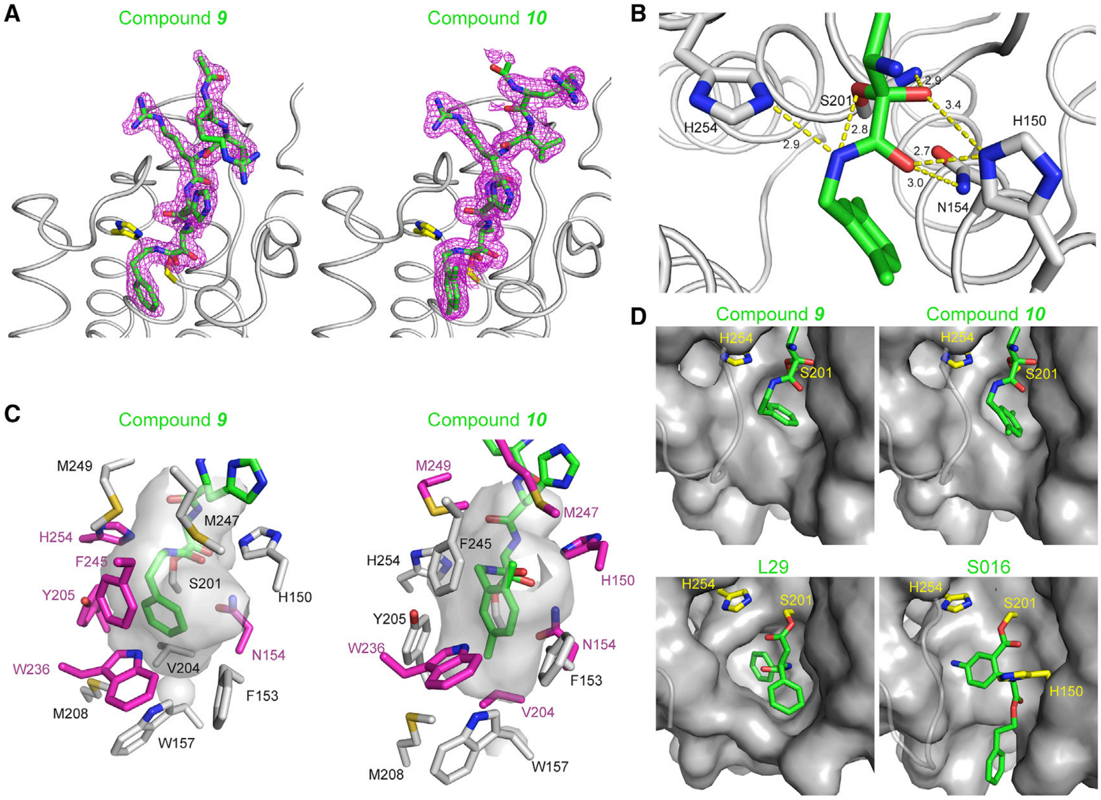

Figure 7. N-Substituted Peptidyl Ketoamides Bind GlpG in a Substrate-like Manner and Occupy the S4 to S2′ Subsites of the Rhomboid Active Site

(A) Electron density map and ligand stick model of **9** and **10** in complex with GlpG. Compounds **9** and **10** were soaked into the native crystals of GlpG, and the structures of the complexes were solved by X-ray diffraction (for statistics, see Table S1). The catalytic dyad is shown as yellow sticks and the inhibitors as green sticks surrounded by the $2mF_o - DF_c$ electron density map contoured at 1σ and shown 1.6 Å around the inhibitor model. In the complex of **9**, the electron density for the Arg residue in the P5 position was weaker, and the side chain has been modeled in a different conformation than in the complex of **10**, which was solved to a higher resolution and where the side chain of the Arg in the P5 position is defined clearly.

(B) Hydrogen bond engagement by the warhead of compound **10** in the active site of GlpG was analyzed using the HBplus program (McDonald and Thornton, 1994) implemented in Ligplot+ (Laskowski and Swindells, 2011) with default criteria (donor … acceptor [D … A] distance cutoff of 3.9 Å; donor … acceptor-acceptor antecedent [D … A-AA] angle of 90°).

(C) Interaction pattern of inhibitor tails in the S2′ site of GlpG. The cavity surrounding the tails of **9** and **10** is shown as an inverse surface, and the side chains lining the cavity are shown as sticks. The residues engaged in van der Waals interactions (identified by Ligplot+) with the tails of the inhibitors are shown in magenta.

(D) Comparison of binding modes of the S2′ binding moieties in compounds **9**, **10**, L29 (Pierrat et al., 2011), and S016 (Vosyka et al., 2013) in the respective complex structures PDB: 5MT6, 5MTF, 3ZMI, and 3ZEB. Protein is showed as a gray surface, catalytic dyad carbons in yellow, and ligand carbons in green. The L5 loop residues 245–250 are shown as semitransparent loops for clarity. All structures are oriented in the same way.

as the significantly larger groups of inhibitors L29 and S016. This alignment shows that the prime side of the GlpG active site is rather malleable, and larger or branched tails could be accommodated at the amide nitrogen of peptidyl ketoamides. This is likely to provide additional selectivity or binding energy and delineates one possible direction of further development of ketoamides as rhomboid inhibitors. The results presented here open the door to systematic development of rhomboid protease inhibitors in medically relevant contexts such as malaria (Baker et al., 2006; O'Donnell et al., 2006), Parkinson's disease (Chu, 2010; Meissner et al., 2015), and cancer (Song et al., 2015).

DISCUSSION

Here, we discover that peptidyl ketoamides bearing a substantial hydrocarbon modification at the ketoamide nitrogen are efficient inhibitors of rhomboid intramembrane proteases, superior to any known rhomboid inhibitors in selectivity and by up to three orders of magnitude in potency. We also show that both of these properties are tunable by optimization of the peptide sequence and the character of the ketoamide "tail" substituent, defining a platform for the development of specific and potent rhomboid inhibitors. Since ketoamides are clinically used pharmacophores (Njoroge et al., 2008), our discovery of this pharmacologically compliant chemotype for rhomboid proteases enables the design of rhomboid inhibitors for cell biological and pharmacological use.

Structural analysis of peptidyl ketoamides complexed to GlpG reveals that they bind in a substrate-like manner, occupying the P4 to P2′ subsites (Figure 7A). The presence of residues in the P5 and P6 positions has been reported to improve the inhibition potency of peptidyl aldehydes significantly, but these residues

could not be observed in any co-crystal structures (Cho et al., 2016). We do observe weak electron density for the side chain of Arg in the P5 position, but its conformation in the final crystallographic models of the complexes of **9** and **10** differs, indicating some degree of flexibility, and it is probably influenced by crystal contacts. In addition, the P5 residue does not contribute significantly to the inhibition potency of **9** (Figure 3B) and is thus dispensable.

The binding mode of peptidyl ketoamides suggests that they can access the rhomboid active site from bulk solvent, and probably do not need prior partitioning into the membrane. They are covalent (Figure 7) and reversible (Figure 4C), and their kinetics of binding to rhomboid is adequately described by a one-step slow-binding mechanism (Figure 4D). Their inhibition modality is non-competitive (Copeland, 2013a) (Figure 4E), implying that they can bind to the free enzyme as well as the docking/interrogation complex during rhomboid catalysis (Strisovsky, 2016a). This is consistent with the proposed mechanism of inhibition of rhomboid protease GlpG by peptidyl aldehydes (Cho et al., 2016).

For the development of peptidyl ketoamides as rhomboid inhibitors, subsite preferences of the given rhomboid protease must be mapped efficiently. This could be achieved using classical positional scanning peptide libraries starting from a known substrate sequence. Given that the effects of the amino acids in the P5–P1 positions are additive (Figure 1A), the optimal substrate could be formed by combining the single subsite preferences identified in the positional scan. An alternative method for mapping subsite preferences at both the prime and non-prime sides could be multiplex substrate profiling using designed peptide libraries and mass spectrometry (O'Donoghue et al., 2012), although its application to rhomboids has not been tested yet.

The second module determining the potency and selectivity is the tail substituent at the ketoamide nitrogen. Here, the effects of flexibility versus rigidity, branching, and polarity of the substituents need to be investigated to explore the available chemical and conformational space. A more speculative direction of further improvement of the inhibitors may involve cyclization via the tail substituent and the P2 residue, which seems sterically possible and unobstructive in the enzyme-inhibitor complex (Figure 7A). Such cyclization could improve the potency of the inhibitor by conformationally restricting it near the bound conformation.

Finally, peptidyl ketoamides have been used clinically to treat hepatitis C infection (boceprevir, telaprevir) (Njoroge et al., 2008), suggesting that both the intracellular availability and metabolic stability of rhomboid-targeting peptidyl ketoamides can most likely be modified for compliance with pharmacological needs. The potential of rhomboid inhibitors in pharmacologically relevant settings has yet to be proven, but it currently seems that inhibitors of *Plasmodium* rhomboids might be therapeutic for malaria (Baker et al., 2006; Lin et al., 2013), inhibitors of the human mitochondrial rhomboid protease PARL might stimulate mitophagy (Meissner et al., 2015) and thus be disease-modifying in the context of Parkinson's disease (Chan and McQuibban, 2013), and inhibitors of human RHBDL4 could be targeting EGF receptor signaling by transforming growth factor α in colorectal cancer (Song et al., 2015). Specific rhomboid protease inhibitors such as those that we describe here will serve as key tools for the validation and exploitation of these and other upcoming therapeutic opportunities involving rhomboid proteases.

SIGNIFICANCE

Intramembrane proteases of the rhomboid family are widely conserved and have been implicated in malaria, colon cancer, and Parkinson's disease. They represent potentially attractive drug targets, but until now, no specific, potent, and pharmacologically compatible inhibitors have been available. Here, we discover that peptidyl ketoamides are the first such potent and specific inhibitors of rhomboid proteases, and we delineate a general modular way for their design against diverse rhomboid enzymes. This discovery can have a broad impact on the cell biology of rhomboid proteases and on drug discovery targeting this family of enzymes in the context of infectious diseases, cancer, and neurodegeneration.

STAR★METHODS

Detailed methods are provided in the online version of this paper and include the following:

- KEY RESOURCES TABLE
- CONTACT FOR REAGENTS AND RESOURCE SHARING
- EXPERIMENTAL MODEL AND SUBJECT DETAILS
- METHOD DETAILS
  - Constructs and Cloning
  - Protein Expression and Purification
  - Chemical Synthesis
  - Protein Crystallography
  - Rhomboid Activity and Inhibition Assays
  - Inhibitor Selectivity Profiling
- QUANTIFICATION AND STATISTICAL ANALYSIS
- DATA AND SOFTWARE AVAILABILITY

SUPPLEMENTAL INFORMATION

Supplemental Information includes one table and supplemental text and can be found with this article online at https://doi.org/10.1016/j.chembiol.2017.09.007.

AUTHOR CONTRIBUTIONS

K.S. conceived and coordinated the study, designed and evaluated experiments, and wrote the paper with the input of the co-authors. P.M. and S.S. designed and S.S. performed all chemical syntheses, and A.T. designed, performed, and evaluated all kinetics and inhibition measurements with the help of K.Š. and J.Š. in the initial stages. K.R.V., D.C.M., and P.P. performed all crystallographic experiments and evaluated the data with the input of M.L. M.T.N.N. and S.H.L.V. performed selectivity profiling against rhomboid proteases, J.B. performed all experiments on *B. subtilis*, and D.C.J. and D.A.B. performed and evaluated the EnPlex experiments.

ACKNOWLEDGMENTS

We thank Mirka Blechová and Lenka Monincová for peptide synthesis and HPLC/MS analyses, Radko Souček for amino acid analysis, Jiří Brynda for help with crystallographic data analysis, Pavel Srb and Marek Ingr for help and advice on data fitting and kinetics, Petra Rampírová for DNA cloning

and laboratory assistance, Libor Krásný and Tom Silhavy for reagents and advice, and Colin Adrain and Cyril Bařinka for critical reading of the manuscript. K.S. was a recipient of a Purkyne Fellowship of the Academy of Sciences of the Czech Republic and acknowledges support also from EMBO (installation grant no. 2329), Ministry of Education, Youth and Sports of the Czech Republic (project nos. LK11206 and LO1302), Marie Curie Career Integration Grant (project no. 304154), Gilead Sciences & IOCB Research Centre, and the National Subvention for Development of Research Organisations (RVO: 61388963) to the Institute of Organic Chemistry and Biochemistry. P.P. acknowledges support from the Ministry of Education of the Czech Republic (program "NPU I"), project no. LO1304, and J.S. and J.B. acknowledge support from the Grant Agency of Charles University (GA UK) in Prague (PhD grant project nos. 232313 and 170214). K.R.V. was supported by an MRC grant (MC_U105184322) as part of R. Henderson's group. D.A.B. was supported by Josie Robertson Foundation and the MSKCC (core grant P30 CA008748), and D.C.J. by the NIH (T32 GM115327-Tan). M.L. was supported by the Czech Science Foundation (grant number P208/12/G016). M.T.N.N. and S.H.L.V. are supported by the Deutsche Forschungsgemeinschaft and the Ministerium für Innovation, Wissenschaft und Forschung des Landes Nordrhein-Westfalen.

Received: April 13, 2017
Revised: August 19, 2017
Accepted: September 18, 2017
Published: October 26, 2017

SUPPORTING CITATIONS

The following references appear in the Supplemental Information: Bastiaans et al., 1997; Cao et al., 2010; Coste et al., 1994; D'Andrea and Scola, 2008; Dondoni and Perrone, 1993; Semple et al., 2000; Souček and Urban, 1995; Tulla-Puche et al., 2008; Venkatraman et al., 2006; Yin et al., 2007.

REFERENCES

Ahlrichs, R., Bär, M., Häser, M., Horn, H., and Kölmel, C. (1989). Electronic structure calculations on workstation computers: the program system turbomole. Chem. Phys. Lett. *162*, 165–169.

Bachovchin, D.A., Koblan, L.W., Wu, W., Liu, Y., Li, Y., Zhao, P., Woznica, I., Shu, Y., Lai, J.H., Poplawski, S.E., et al. (2014). A high-throughput, multiplexed assay for superfamily-wide profiling of enzyme activity. Nat. Chem. Biol. *10*, 656–663.

Baker, R.P., Wijetilaka, R., and Urban, S. (2006). Two *Plasmodium* rhomboid proteases preferentially cleave different adhesins implicated in all invasive stages of malaria. PLoS Pathog. *2*, e113.

Bastiaans, H.M.M., vanderBaan, J.L., and Ottenheijm, H.C.J. (1997). Flexible and convergent total synthesis of cyclotheonamide B. J. Org. Chem. *62*, 3880–3889.

Cao, H., Liu, H., and Domling, A. (2010). Efficient multicomponent reaction synthesis of the schistosomiasis drug praziquantel. Chemistry *16*, 12296–12298.

Chan, E.Y., and McQuibban, G.A. (2013). The mitochondrial rhomboid protease: its rise from obscurity to the pinnacle of disease-relevant genes. Biochim. Biophys. Acta *1828*, 2916–2925.

Chatterjee, S., Dunn, D., Tao, M., Wells, G., Gu, Z.Q., Bihovsky, R., Ator, M.A., Siman, R., and Mallamo, J.P. (1999). P2-achiral, P'-extended alpha-ketoamide inhibitors of calpain I. Bioorg. Med. Chem. Lett. *9*, 2371–2374.

Cho, S., Dickey, S.W., and Urban, S. (2016). Crystal structures and inhibition kinetics reveal a two-stage catalytic mechanism with drug design implications for rhomboid proteolysis. Mol. Cell *61*, 329–340.

Chu, C.T. (2010). A pivotal role for PINK1 and autophagy in mitochondrial quality control: implications for Parkinson disease. Hum. Mol. Genet. *19*, R28–R37.

Copeland, R.A. (2013a). Reversible modes of inhibitor interactions with enzymes. In Evaluation of Enzyme Inhibitors in Drug Discovery (John Wiley), pp. 57–121.

Copeland, R.A. (2013b). Slow binding inhibitors. In Evaluation of Enzyme Inhibitors in Drug Discovery (John Wiley), pp. 203–244.

Coste, J., Frerot, E., and Jouin, P. (1994). Coupling N-methylated amino-acids using pybrop and pyclop halogenophosphonium salts - mechanism and fields of application. J. Org. Chem. *59*, 2437–2446.

D'Andrea, S., and Scola, P.M.. (2008). Inhibitors of hepatitis C virus. Bristol-Myers Squibb Company. US patent US2008107623 (A1), filed October 25, 2007, and published May 8, 2008.

Dickey, S.W., Baker, R.P., Cho, S., and Urban, S. (2013). Proteolysis inside the membrane is a rate-governed reaction not driven by substrate affinity. Cell *155*, 1270–1281.

Dondoni, A., and Perrone, D. (1993). 2-Thiazolyl alpha-amino ketones - a new class of reactive intermediates for the stereocontrolled synthesis of unusual amino-acids. Synthesis (Stuttg), 1162–1176.

Drag, M., and Salvesen, G.S. (2010). Emerging principles in protease-based drug discovery. Nat. Rev. Drug Discov. *9*, 690–701.

Eggert, U.S., Ruiz, N., Falcone, B.V., Branstrom, A.A., Goldman, R.C., Silhavy, T.J., and Kahne, D. (2001). Genetic basis for activity differences between vancomycin and glycolipid derivatives of vancomycin. Science *294*, 361–364.

Emsley, P., and Cowtan, K. (2004). Coot: model-building tools for molecular graphics. Acta Crystallogr. Sect. D Biol. Crystallogr. *60*, 2126–2132.

Evans, P.R. (2011). An introduction to data reduction: space-group determination, scaling and intensity statistics. Acta Crystallogr. Sect. D Biol. Crystallogr. *67*, 282–292.

Fanfrlik, J., Holub, J., Ruzickova, Z., Rezac, J., Lane, P.D., Wann, D.A., Hnyk, D., Ruzicka, A., and Hobza, P. (2016). Competition between halogen, hydrogen and dihydrogen bonding in brominated carboranes. ChemPhysChem *17*, 3373–3376.

Fleig, L., Bergbold, N., Sahasrabudhe, P., Geiger, B., Kaltak, L., and Lemberg, M.K. (2012). Ubiquitin-dependent intramembrane rhomboid protease promotes ERAD of membrane proteins. Mol. Cell *47*, 558–569.

Gerwig, J., Kiley, T.B., Gunka, K., Stanley-Wall, N., and Stulke, J. (2014). The protein tyrosine kinases EpsB and PtkA differentially affect biofilm formation in Bacillus subtilis. Microbiology *160*, 682–691.

Gibson, D.G. (2011). Enzymatic assembly of overlapping DNA fragments. Methods Enzymol. *498*, 349–361.

Grimme, S. (2006). Semiempirical GGA-type density functional constructed with a long-range dispersion correction. J. Comput. Chem. *27*, 1787–1799.

Harper, J.W., and Powers, J.C. (1985). Reaction of serine proteases with substituted 3-alkoxy-4-chloroisocoumarins and 3-alkoxy-7-amino-4-chloroisocoumarins: new reactive mechanism-based inhibitors. Biochemistry *24*, 7200–7213.

Harper, J.W., Hemmi, K., and Powers, J.C. (1985). Reaction of serine proteases with substituted isocoumarins: discovery of 3,4-dichloroisocoumarin, a new general mechanism based serine protease inhibitor. Biochemistry *24*, 1831–1841.

Hedstrom, L. (2002). Serine protease mechanism and specificity. Chem. Rev. *102*, 4501–4524.

Jensen, F. (2006). Introduction to Computational Chemistry (Wiley).

Kabsch, W. (2010). Xds. Acta Crystallogr. Sect. D Biol. Crystallogr. *66*, 125–132.

Klamt, A., and Schüürmann, G. (1993). COSMO: a new approach to dielectric screening in solvents with explicit expressions for the screening energy and its gradient. J. Chem. Soc. Perkin Trans. *2*, 799–805.

Krissinel, E., and Henrick, K. (2004). Secondary-structure matching (SSM), a new tool for fast protein structure alignment in three dimensions. Acta Crystallogr. Sect. D Biol. Crystallogr. *60*, 2256–2268.

Laskowski, R.A., and Swindells, M.B. (2011). LigPlot+: multiple ligand-protein interaction diagrams for drug discovery. J. Chem. Inf. Model *51*, 2778–2786.

Lebedev, A.A., Young, P., Isupov, M.N., Moroz, O.V., Vagin, A.A., and Murshudov, G.N. (2012). JLigand: a graphical tool for the CCP4 template-restraint library. Acta Crystallogr. Sect. D Biol. Crystallogr. *68*, 431–440.

Lee, C., Kang, H.J., Hjelm, A., Qureshi, A.A., Nji, E., Choudhury, H., Beis, K., de Gier, J.W., and Drew, D. (2014). MemStar: a one-shot *Escherichia coli*-based approach for high-level bacterial membrane protein production. FEBS Lett. *588*, 3761–3769.

Lemberg, M.K., Menendez, J., Misik, A., Garcia, M., Koth, C.M., and Freeman, M. (2005). Mechanism of intramembrane proteolysis investigated with purified rhomboid proteases. EMBO J. *24*, 464–472.

Lin, J.W., Meireles, P., Prudencio, M., Engelmann, S., Annoura, T., Sajid, M., Chevalley-Maurel, S., Ramesar, J., Nahar, C., Avramut, C.M., et al. (2013). Loss-of-function analyses defines vital and redundant functions of the *Plasmodium* rhomboid protease family. Mol. Microbiol. *88*, 318–338.

Liu, Y., Stoll, V.S., Richardson, P.L., Saldivar, A., Klaus, J.L., Molla, A., Kohlbrenner, W., and Kati, W.M. (2004). Hepatitis C NS3 protease inhibition by peptidyl-alpha-ketoamide inhibitors: kinetic mechanism and structure. Arch. Biochem. Biophys. *421*, 207–216.

McCoy, A.J. (2007). Solving structures of protein complexes by molecular replacement with Phaser. Acta Crystallogr. Sect. D Biol. Crystallogr. *63*, 32–41.

McDonald, I.K., and Thornton, J.M. (1994). Satisfying hydrogen bonding potential in proteins. J. Mol. Biol. *238*, 777–793.

Meissner, C., Lorenz, H., Hehn, B., and Lemberg, M.K. (2015). Intramembrane protease PARL defines a negative regulator of PINK1- and PARK2/Parkin-dependent mitophagy. Autophagy *11*, 1484–1498.

Miroux, B., and Walker, J.E. (1996). Over-production of proteins in *Escherichia coli*: mutant hosts that allow synthesis of some membrane proteins and globular proteins at high levels. J. Mol. Biol. *260*, 289–298.

Mitchell, E.M., Artymiuk, P.J., Rice, D.W., and Willett, P. (1990). Use of techniques derived from graph-theory to compare secondary structure motifs in proteins. J. Mol. Biol. *212*, 151–166.

Morrison, J.F. (1982). The slow-binding and slow, tight-binding inhibition of enzyme-catalysed reactions. Trends Biochem. Sci. *7*, 102–105.

Murshudov, G.N., Skubak, P., Lebedev, A.A., Pannu, N.S., Steiner, R.A., Nicholls, R.A., Winn, M.D., Long, F., and Vagin, A.A. (2011). REFMAC5 for the refinement of macromolecular crystal structures. Acta Crystallogr. Sect. D Biol. Crystallogr. *67*, 355–367.

Nguyen, M.T., Van Kersavond, T., and Verhelst, S.H. (2015). Chemical tools for the study of intramembrane proteases. ACS Chem. Biol. *10*, 2423–2434.

Njoroge, F.G., Chen, K.X., Shih, N.Y., and Piwinski, J.J. (2008). Challenges in modern drug discovery: a case study of boceprevir, an HCV protease inhibitor for the treatment of hepatitis C virus infection. Acc. Chem. Res. *41*, 50–59.

O'Donnell, R.A., Hackett, F., Howell, S.A., Treeck, M., Struck, N., Krnajski, Z., Withers-Martinez, C., Gilberger, T.W., and Blackman, M.J. (2006). Intramembrane proteolysis mediates shedding of a key adhesin during erythrocyte invasion by the malaria parasite. J. Cell Biol. *174*, 1023–1033.

O'Donoghue, A.J., Eroy-Reveles, A.A., Knudsen, G.M., Ingram, J., Zhou, M., Statnekov, J.B., Greninger, A.L., Hostetter, D.R., Qu, G., Maltby, D.A., et al. (2012). Global identification of peptidase specificity by multiplex substrate profiling. Nat. Methods *9*, 1095–1100.

Pierrat, O.A., Strisovsky, K., Christova, Y., Large, J., Ansell, K., Bouloc, N., Smiljanic, E., and Freeman, M. (2011). Monocyclic beta-lactams are selective, mechanism-based inhibitors of rhomboid intramembrane proteases. ACS Chem. Biol. *6*, 325–335.

Powers, J.C., Kam, C.M., Narasimhan, L., Oleksyszyn, J., Hernandez, M.A., and Ueda, T. (1989). Mechanism-based isocoumarin inhibitors for serine proteases: use of active site structure and substrate specificity in inhibitor design. J. Cell. Biochem. *39*, 33–46.

Powers, J.C., Asgian, J.L., Ekici, O.D., and James, K.E. (2002). Irreversible inhibitors of serine, cysteine, and threonine proteases. Chem. Rev. *102*, 4639–4750.

Purich, D.L. (2010). Kinetic behavior of enzyme inhibitors. In Enzyme Kinetics: Catalysis & Control (Elsevier), pp. 485–574.

Ruiz, N., Falcone, B., Kahne, D., and Silhavy, T.J. (2005). Chemical conditionality. Cell *121*, 307–317.

Schechter, I., and Berger, A. (1967). On the size of the active site in proteases. I. Papain. Biochem. Biophys. Res. Commun. *27*, 157–162.

Schneider, C.A., Rasband, W.S., and Eliceiri, K.W. (2012). NIH Image to ImageJ: 25 years of image analysis. Nat. Methods *9*, 671–675.

Schrodinger. (2012). The PyMOL Molecular Graphics System, Version 1.5.0.4 (Schrödinger LLC).

Semple, J.E., Owens, T.D., Nguyen, K., and Levy, O.E. (2000). New synthetic technology for efficient construction of alpha-hydroxy-beta-amino amides via the Passerini reaction. Org. Lett. *2*, 2769–2772.

Serim, S., Haedke, U., and Verhelst, S.H. (2012). Activity-based probes for the study of proteases: recent advances and developments. ChemMedChem *7*, 1146–1159.

Singh, J., Petter, R.C., Baillie, T.A., and Whitty, A. (2011). The resurgence of covalent drugs. Nat. Rev. Drug Discov. *10*, 307–317.

Song, W., Liu, W., Zhao, H., Li, S., Guan, X., Ying, J., Zhang, Y., Miao, F., Zhang, M., Ren, X., et al. (2015). Rhomboid domain containing 1 promotes colorectal cancer growth through activation of the EGFR signalling pathway. Nat. Commun. *6*, 8022.

Souček, M., and Urban, J. (1995). An efficient method for preparation of optically active N-protected α-amino aldehydes from N-protected α-amino alcohols. Collect. Czechoslov. Chem. Commun. *60*, 693–696.

Stevenson, L.G., Strisovsky, K., Clemmer, K.M., Bhatt, S., Freeman, M., and Rather, P.N. (2007). Rhomboid protease AarA mediates quorum-sensing in *Providencia stuartii* by activating TatA of the twin-arginine translocase. Proc. Natl. Acad. Sci. USA *104*, 1003–1008.

Strisovsky, K. (2013). Structural and mechanistic principles of intramembrane proteolysis - lessons from rhomboids. FEBS J. *280*, 1579–1603.

Strisovsky, K. (2016a). Rhomboid protease inhibitors: emerging tools and future therapeutics. Semin. Cell Dev. Biol. *60*, 52–62.

Strisovsky, K. (2016b). Why cells need intramembrane proteases - a mechanistic perspective. FEBS J. *283*, 1837–1845.

Strisovsky, K., Sharpe, H.J., and Freeman, M. (2009). Sequence-specific intramembrane proteolysis: identification of a recognition motif in rhomboid substrates. Mol. Cell *36*, 1048–1059.

Ticha, A., Stanchev, S., Skerle, J., Began, J., Ingr, M., Svehlova, K., Polovinkin, L., Ruzicka, M., Bednarova, L., Hadravova, R., et al. (2017). Sensitive versatile fluorogenic transmembrane peptide substrates for rhomboid intramembrane proteases. J. Biol. Chem. *292*, 2703–2713.

Tulla-Puche, J., Torres, A., Calvo, P., Royo, M., and Albericio, F. (2008). N,N,N',N'-Tetramethylchloroformamidinium hexafluorophosphate (TCFH), a powerful coupling reagent for bioconjugation. Bioconj. Chem. *19*, 1968–1971.

Venkatraman, S., Bogen, S.L., Arasappan, A., Bennett, F., Chen, K., Jao, E., Liu, Y.T., Lovey, R., Hendrata, S., Huang, Y., et al. (2006). Discovery of (1R,5S)-N-[3-amino-1-(cyclobutylmethyl)-2,3-dioxopropyl]- 3-[2(S)-[[[(1,1-dimethylethyl)amino]carbonyl]amino]-3,3-dimethyl-1-oxobutyl]- 6,6-dimethyl-3-azabicyclo[3.1.0]hexan-2(S)-carboxamide (SCH 503034), a selective, potent, orally bioavailable hepatitis C virus NS3 protease inhibitor: a potential therapeutic agent for the treatment of hepatitis C infection. J. Med. Chem. *49*, 6074–6086.

Vinothkumar, K.R., Strisovsky, K., Andreeva, A., Christova, Y., Verhelst, S., and Freeman, M. (2010). The structural basis for catalysis and substrate specificity of a rhomboid protease. EMBO J. *29*, 3797–3809.

Vinothkumar, K.R., Pierrat, O.A., Large, J.M., and Freeman, M. (2013). Structure of rhomboid protease in complex with beta-lactam inhibitors defines the S2' cavity. Structure *21*, 1051–1058.

Vosyka, O., Vinothkumar, K.R., Wolf, E.V., Brouwer, A.J., Liskamp, R.M., and Verhelst, S.H. (2013). Activity-based probes for rhomboid proteases discovered in a mass spectrometry-based assay. Proc. Natl. Acad. Sci. USA *110*, 2472–2477.

Walker, B., and Lynas, J.F. (2001). Strategies for the inhibition of serine proteases. Cell. Mol. Life Sci. *58*, 596–624.

Wang, Y., and Ha, Y. (2007). Open-cap conformation of intramembrane protease GlpG. Proc. Natl. Acad. Sci. USA *104*, 2098–2102.

Wang, Y., Zhang, Y., and Ha, Y. (2006). Crystal structure of a rhomboid family intramembrane protease. Nature *444*, 179–180.

Wolf, E.V., Zeissler, A., Vosyka, O., Zeiler, E., Sieber, S., and Verhelst, S.H. (2013). A new class of rhomboid protease inhibitors discovered by activity-based fluorescence polarization. PLoS One *8*, e72307.

Wolf, E.V., Zeissler, A., and Verhelst, S.H. (2015). Inhibitor fingerprinting of rhomboid proteases by activity-based protein profiling reveals inhibitor selectivity and rhomboid autoprocessing. ACS Chem. Biol. *10*, 2325–2333.

Xue, Y., Chowdhury, S., Liu, X., Akiyama, Y., Ellman, J., and Ha, Y. (2012). Conformational change in rhomboid protease GlpG induced by inhibitor binding to its S' subsites. Biochemistry *51*, 3723–3731.

Yin, J., Gallis, C.E., and Chisholm, J.D. (2007). Tandem oxidation/halogenation of aryl allylic alcohols under Moffatt-Swern conditions. J. Org. Chem. *72*, 7054–7057.

Zoll, S., Stanchev, S., Began, J., Skerle, J., Lepsik, M., Peclinovska, L., Majer, P., and Strisovsky, K. (2014). Substrate binding and specificity of rhomboid intramembrane protease revealed by substrate-peptide complex structures. EMBO J. *33*, 2408–2421.

STAR★METHODS

KEY RESOURCES TABLE

REAGENT or RESOURCE	SOURCE	IDENTIFIER
Antibodies		
Rabbit anti-DYKDDDDK	Cell Signaling Technology	Cat#2368
Monoclonal ANTI-FLAG® M2 antibody produced in mouse	Sigma	Cat#F1804
Donkey anti-Rabbit IgG (H+L) Cross-Adsorbed Secondary Antibody, DyLight 800	Invitrogen	Cat#SA5-10044
Bacterial and Virus Strains		
E. coli NR698	Laboratory of Tom Silhavy (Princeton)	(Ruiz et al., 2005)
E. coli NR698*ÄglpG::tet*	Laboratory of Matthew Freeman (Oxford)	(Pierrat et al., 2011)
Bacillus subtilis 168	Bacillus Genetic Stock Center	
Bacillus subtilis 168 *ydcA::neo*	This work	BS2
Bacillus subtilis 168 *ydcA::neo, yqgP::tet*	This work	BS4
Bacillus subtilis 168 *ydcA::neo*, *xdkE::MBP-LacYTM2-Trx(erm, lin)*	This work	BS87
Bacillus subtilis 168 *ydcA::neo*, *yqgP::tet, xdkE::MBP-LacYTM2-Trx(erm, lin)*	This work	BS88
E. coli C41(DE3)	Lucigen	Cat#60452-1
Chemicals, Peptides, and Recombinant Proteins		
h-KRHDIN(E-edans)ISKSDTG(K-dabcyl) IFAAISLFSLLFQPLFGLSKK-nh_2	(Ticha et al., 2017)	KSp35
h-KRHRI(E-edans)RVRHADTG(K-dabcyl) IFAAISLFSLLFQPLFGLSKKR-nh_2	This work	KSp93
h-KRHRI(K-tamra)RVRHADTG(C-qxl610) IFAAISLFSLLFQPLFGLSKKR-nh_2	(Ticha et al., 2017)	KSp64
h-KRHRINRVR(E-edans)ADTG(K-dabcyl) IFAAISLFSLLFQPLFGLSKKR-nh_2	This work	KSp96
TAMRA-XP	Thermo Fisher Scientific	Cat#88318
Revert Total Protein Stain Kit	LI-COR, Inc.	Cat#926-11010
3,4-dichloroisocoumarin (DCI)	Sigma	D7910
AcRVRHAcmk	This paper	
AcVRHAcmk	This paper	
AcIATAcmk	Zoll et al., 2014	
AcVATAcmk	This paper	
AcIAHAcmk	This paper	
AcIRTAcmk	This paper	
AcRVRHA-trifluoromethylketone	This paper	
AcRVRHA-boronate	This paper	
AcRVRHA-acylsulfonamide	This paper	
AcRVRHA-thiazolylketone	This paper	
AcRVRHA-$CONH_2$	This paper	
Ac-RVRHA-CONH-isopropyl	This paper	
Ac-RVRHA-CONH-cyclohexyl	This paper	
Ac-RVRHA-CONH-benzyl	This paper	
Ac-RVRHA-CONH-methylbenzyl	This paper	
Ac-RVRHA-CONH-ethyloxyacetyl	This paper	

(*Continued on next page*)

Continued

REAGENT or RESOURCE	SOURCE	IDENTIFIER
Ac-RVRHA-CONH-neopentyl	This paper	
Ac-RVRHA-CONH-pentyl	This paper	
Ac-RVRHA-CONH-phenylethyl	This paper	
Ac-RVRHA-CONH-(2,4-dimethyl)benzyl	This paper	
Ac-RVRHA-CONH-phenylbutyl	This paper	
Ac-RHA-CONH-phenylethyl	This paper	
Ac-RHA-CONH-phenylethyl	This paper	
Ac-A-CONH-phenylethyl	This paper	
Deposited Data		
Crystal structure of GlpG bound to AcRVRHAcmk	This paper	PDB: 5MT8
Crystal structure of GlpG bound to AcVRHAcmk	This paper	PDB: 5MT7
Crystal structure of GlpG bound to Ac-RVRHA-CONH-phenylethyl	This paper	PDB: 5MT6
Crystal structure of GlpG bound to Ac-RVRHA-CONH-(2,4-dimethyl)benzyl	This paper	PDB: 5MTF
Recombinant DNA		
pMALp2E_MBP-LacYTM2-Trx	(Zoll et al., 2014)	pKS506
pD881-SR	DNA2.0 Inc.	
pD881-SR_MBP-LacYTM2-Trx	This work	pPR61
pET25b+M_GlpG	(Lemberg et al., 2005)	
pGP886_pxyl-AmyE$_{SP}$-MBP-FLAG-LacYTM2-Trx-HA	This work	pPR200
Software and Algorithms		
XDS	(Kabsch, 2010)	http://xds.mpimf-heidelberg.mpg.de/html_doc/downloading.html
AIMLESS	(Evans, 2011)	http://www.ccp4.ac.uk/html/aimless.html
COOT 0.8.6	(Emsley and Cowtan, 2004)	https://www2.mrc-lmb.cam.ac.uk/personal/pemsley/coot/
Jligand	(Lebedev et al., 2012)	http://www.ysbl.york.ac.uk/mxstat/JLigand/
Refmac_5.8.0158	(Murshudov et al., 2011)	
Ligplot+	(Laskowski and Swindells, 2011)	http://www.ebi.ac.uk/thornton-srv/software/LigPlus/download.html
Phaser	(McCoy, 2007)	http://www.ccp4.ac.uk/html/phaser.html
Pymol 1.8.4.0	Schrodinger, LLC	https://www.pymol.org/
Turbomole 7	(Ahlrichs et al., 1989)	http://www.turbomole.com/
ImageJ 1.49	(Schneider et al., 2012)	https://imagej.net/Welcome
Image Studio Lite Version 5.2	LI-COR, Inc.	https://www.licor.com/bio/products/software/image_studio_lite/download.html
GraphPad Prism 7.02	GraphPad Software, Inc.	https://www.graphpad.com/scientific-software/prism/
GraFit 7	Erithacus Software Ltd.	http://www.erithacus.com/grafit/

CONTACT FOR REAGENTS AND RESOURCE SHARING

Further information and requests for reagents may be directed to, and will be fulfilled by the corresponding author Kvido Strisovsky (kvido.strisovsky@uochb.cas.cz).

EXPERIMENTAL MODEL AND SUBJECT DETAILS

Escherichia coli K12 strain NR698 (Ruiz et al., 2005), which has the MC4100 background with the *imp4213* allele carried from BE100 (Eggert et al., 2001) (an in-frame deletion of amino acids 330-352 of LptD) is a gift of Dr. Tom Silhavy (Princeton University). A GlpG-free variant was created by deleting *glpG* using a tetracyclin marker (Pierrat et al., 2011).

To generate a rhomboid activity free *Bacillus subtilis*, the *ydcA::neo* mutant (BS2, this work) of the wild type B*. subtilis* 168 strain (Bacillus Genetic Stock Center, USA) was modified by deleting the entire the *yqgP* gene and replacing it with a tetracyclin resistance gene using homologous recombination, yielding strain BS4 (*ydcA::neo*, *yqgP::tet*). Both modifications were verified by genomic PCR of the disrupted locus and Sanger sequencing of the amplified region.

METHOD DETAILS

Constructs and Cloning

To generate a model rhomboid substrate for *in vivo* activity assays in E*. coli*, the MBP-LacYTM2-Trx-Stag-Histag construct was PCR-amplified from pKS506 (Ticha et al., 2017) and cloned into the SapI linearized plasmid pD881-SR (DNA2.0 Inc., Newark, USA) using isothermal assembly (Gibson, 2011), yielding construct pPR61. For expression in B*. subtilis*, the substrate was modified by replacing the MBP signal peptide by the signal peptide from B*. subtilis* AmyE, and the AmyE$_{sp}$-MBP$_{mat}$-FLAG-LacYTM2-Trx-HA construct was cloned into the XbaI, SalI digested plasmid pGP886 (Gerwig et al., 2014) (gift of Dr. Libor Krasny, Prague, CR) using isothermal assembly (Gibson, 2011) to yield construct pPR200. This construct was linearized by ScaI and integrated into the *xkdE* locus of BS2 and BS4 using erythromycin-lincomycin selection yielding strains BS87 (BS2 *xkdE::Pxyl-LacYTM2(erm)*) and BS88 (BS4 *xkdE::Pxyl-LacYTM2(erm)*).

Protein Expression and Purification

The *E. coli* GlpG for crystallisation was expressed in *E. coli* C41(DE3) (Miroux and Walker, 1996) in PASM 5052 medium as described (Lee et al., 2014). Membrane isolation, purification by metal affinity chromatography, cleavage by chymotrypsin to produce GlpG transmembrane core domain and gel filtration chromatography were carried out as described previously (Vinothkumar et al., 2010; Zoll et al., 2014). The *E. coli* GlpG for inhibition studies was expressed in *E. coli* C41(DE3) (Miroux and Walker, 1996) in LB medium, and solubilised and purified in 0.05% (w/v) DDM as described (Ticha et al., 2017). Other rhomboid proteases were expressed and purified as reported previously (Wolf et al., 2015).

Chemical Synthesis

All reagents were acquired from commercial sources and used without purification. Protected amino acids and amino acid derivatives were purchased from Iris Biotech (Marktredwitz, Germany), Sigma-Aldrich (St. Louis, MO, U.S.A), Thermo Fischer Scientific (Waltham, Massachusets, U.S.A) and Fluorochem (Hadfield, Derbyshire, UK). Further details on chemical syntheses as well as compound characterisation data by mass spectrometry and NMR are available as Supplemental Information (Methods S1).

Protein Crystallography

Crystals of truncated wild type GlpG apoenzyme were obtained by mixing a solution of 2 - 3 M ammonium chloride or sodium chloride, 0.1 M Bis-Tris, pH 7.0 with protein (4-6 mg/mL) at ratio of 1:1 in hanging drops at 22°C (Vinothkumar et al., 2010; Wang and Ha, 2007). Inhibitors were diluted from 10 mM stock solutions in anhydrous DMSO into buffer resembling the mother liquor to yield final 1 mM inhibitor and 10% DMSO just before soaking. For the chloromethylketone inhibitors, the crystals were incubated with inhibitors at 0.3-0.5 mM concentrations for 24 h. The ketoamide inhibitors were incubated at final concentrations of 0.3-0.5 mM for 30-120 min. All crystals were cryo-protected by adding 25% (v/v) glycerol to the mother liquor and flash frozen in liquid nitrogen.

Data sets of the CMKs and ***9*** were collected at the I02 beam line at the Diamond Light Source (Harwell) and the data set of ***10*** was collected at BESSY (Berlin, Germany). Diffraction data were indexed, integrated and scaled with XDS (Kabsch, 2010) and AIMLESS (Evans, 2011). For the structures with inhibitor bound, the coordinates of GlpG (PDB 2XOV) with residues 245-249 (of Loop 5) omitted were used as an input model for Phaser (McCoy, 2007). Restrained refinement was carried out with Refmac (Murshudov et al., 2011) followed by manual model building in COOT (Emsley and Cowtan, 2004). In the final step, TLS was used using the enzyme and the inhibitor peptide as one group (Murshudov et al., 2011). The model, library and link files of the inhibitors were generated with Jligand (Lebedev et al., 2012). In the structures of Ac-(R)VRHA-cmk, H150 was modelled to hydrogen bond to the chloromethylketone oxygen. An additional density was observed close to M149 and H150 raising the possibility that the H150 residue could be also in an alternative conformation, but modeling the alternative conformation or both conformations of H150 was not conclusive in explaining the density. Other similar datasets of CMKs obtained by soaking show that this density might perhaps represent a bound ion, but due to ambiguity we have left the density unmodelled.

In order to find the best possible fit of the molecules of ***9*** and ***10*** to the experimental electron densities, quantum mechanical calculations were performed. The model systems comprised the whole inhibitors in their tetrahedral intermediate form with methoxy group representing the S201 side-chain. These models were made in several variants: i) *cis/trans* isomers of the ketoamide proximal/distal carbonyls, ii) *cis/trans* isomers of the distal carbonyl/NH, and iii) different rotameric forms of the His side chain in the P2 position of the inhibitors. All these variants were optimized in Turbomole ver. 7 program (Ahlrichs et al., 1989) using DFT-D3 method (Grimme, 2006) at B-LYP/DZVP level (Fanfrlik et al., 2016; Jensen, 2006) and COSMO implicit solvent model (Klamt and Schüürmann, 1993). Their intrinsic stabilities were assessed by comparing the final energies, and the conformer with the lowest energy was built into the electron density and chosen as a model for crystallographic refinement.

Noncovalent interactions between the ligands and protein were detected using Ligplot+ (Laskowski and Swindells, 2011) and hydrogen bonds were defined by canonical geometrical criteria (Laskowski and Swindells, 2011; McDonald and Thornton, 1994).

Structural alignments and all structure figures were made with Pymol (Schrodinger, 2012). The coordinates of the structures presented in this manuscript have been deposited in the PDB under the following IDs: 5MT7 (Ac-VRHA-cmk), 5MT8 (Ac-RVRHA-cmk), 5MT6 (compound ***9***) and 5MTF (compound ***10***). Data collection and refinement statistics are listed in Table S1.

Rhomboid Activity and Inhibition Assays

The activity of GlpG *in vitro* was determined as reported (Ticha et al., 2017). Concentrations of stock solutions of peptide substrates and inhibitors were determined by quantitative amino acid analysis. The IC_{50} and reversibility measurements were performed in 20 mM HEPES, pH 7.4, 150 mM NaCl, 0.05%(w/v) DDM, 12%(v/v) DMSO, and other kinetic measurements in 50 mM potassium phosphate, pH 7.4, 150 mM NaCl, 0.05%(w/v) DDM, 10%(v/v) DMSO, 0.05%(w/v) PEG8000, and 20%(v/v) glycerol unless noted otherwise. The reaction mixture typically consisted of 10 μM fluorogenic peptide substrate and the measurements were performed without enzyme-inhibitor pre-incubation unless noted otherwise. Note that the fluorogenic substrates used in Figure 4 had nearly identical amino acid sequences but for the point of attachment of the fluorophore or the identity of the fluorophore and quencher (see Key Resources Table).

For measuring the inhibition of GlpG *in vivo*, the *E. coli* strain NR698 with genetically permeabilised outer membrane (Ruiz et al., 2005) and its *glpG* knock-out derivative KS69 (*glpG::tet*) were used as described (Pierrat et al., 2011) with the following modifications. The chimeric substrate encoding LacY transmembrane domain 2 inserted between maltose binding protein and thioredoxin (Strisovsky et al., 2009) was expressed under control of rhamnose promoter (construct pPR61). To evaluate the *in vivo* inhibition by ketoamides, the NR698 cells were inoculated to the density of OD_{600} = 0.05 and grown to OD_{600}=0.6 at 37°C. The cells were then incubated with increasing concentrations of inhibitor for 15 min at room temperature, and expression of the chimeric substrate was induced by adding 1 mM L-rhamnose. Cells were grown for further 4 h at 25°C, after which steady-state level of substrate cleavage was evaluated by western blotting with near-infrared fluorescence detection as described (Ticha et al., 2017).

For measuring the inhibition of YqgP *in vivo*, the B. *subtilis* strains BS87 and its *yqgP* knock-out derivative BS88, generated in this work (see Constructs and Cloning section), were used as follows. The chimeric LacYTM2 substrate $AmyE_{sp}$-MBP-FLAG-LacYTM2-Trx-HA (this work) was expressed under control of xylose promoter from the *xdkE* genomic locus. Fresh LB medium, supplemented with appropriate antibiotic, was inoculated with a few colonies of the B. *subtilis* strain grown overnight on selective LB agar plate and pre-culture was grown for 2 h at 37°C to OD_{600} = 1. Pre-culture was then diluted with fresh LB medium to the density of OD_{600} = 0.05. At this point, the expression of LacYTM2 was induced by adding 1% (w/v) D-(+)-xylose (Sigma), rhomboid inhibitors were added at a range of concentrations, and the cultures were further incubated for 2.5 h at 37°C (reaching $OD_{600} \sim 1$). Steady-state conversion of the substrate was evaluated by western blotting with near-infrared fluorescence detection as described (Ticha et al., 2017), substracting the intensity of non-specific bands, closely co-migrating with the specific rhomboid-formed N-terminal cleavage product of the substrate.

Inhibitor Selectivity Profiling

For inhibitor selectivity profiling against rhomboid proteases (Wolf et al., 2015), 400 ng of a purified protein preparation of *E. coli* GlpG was diluted in 30 μL of reaction buffer (20 mM HEPES, pH 7.4, with 0.05% (w/v) DDM). For other rhomboids, amounts were taken that gave similar labeling intensity during profiling. Rhomboids were incubated for 30 min at room temperature with the indicated concentration of compound, 100 μM DCI as positive control, or an equal volume of DMSO as negative control. Next, TAMRA-FP serine hydrolase probe (Thermo Fisher #88318) was added to a final concentration of 1 ìM and incubated for 2 h at 37°C in the dark. The reaction was stopped by addition of 4× Laemmli buffer and the reaction mixture was resolved on 15% SDS-PAGE. Gels were scanned on a Typhoon Trio+ and analyzed using ImageJ. The intensity of each rhomboid protease band calculated by ImageJ was normalized against its corresponding DMSO-treated counterpart (100% activity) to indicate the residual activity left after inhibition. The remaining activity was used to calculate the percentage of inhibition depicted in the heatmap. Selectivity profiles against human serine hydrolases were determined by EnPlex as described previously (Bachovchin et al., 2014).

QUANTIFICATION AND STATISTICAL ANALYSIS

Enzyme kinetics and inhibition data were analysed in GraphPad Prism v7.02 using in-built algorithms. Means and standard deviations have been derived from the best fit of the data, or based on three independent measurements, as specified, unless noted otherwise. Quantitative western blots were evaluated using near infrared detection with the IRDye 800CW secondary antibody on a LiCor Odyssey CLx infrared scanner with normalisation to total protein using the Revert total protein stain (LiCor).

DATA AND SOFTWARE AVAILABILITY

All crystallographic coordinates of the protein structures presented in this manuscript have been deposited in and will be freely available from the Protein Data Bank (www.rcsb.org) under the following identifiers: 5MT7, 5MT8, 5MT6 and 5MTF.

Cell Chemical Biology, Volume *24*

Supplemental Information

General and Modular Strategy for Designing Potent, Selective, and Pharmacologically Compliant Inhibitors of Rhomboid Proteases

Anežka Tichá, Stancho Stanchev, Kutti R. Vinothkumar, David C. Mikles, Petr Pachl, Jakub Began, Jan Škerle, Kateřina Švehlová, Minh T.N. Nguyen, Steven H.L. Verhelst, Darren C. Johnson, Daniel A. Bachovchin, Martin Lepšík, Pavel Majer, and Kvido Strisovsky

Supplementary Information

Methods S1

Chemical Synthesis

Fluorogenic substrates KSp35 (Ticha, et al., 2017), KSp93 (KRHRI(E-EDANS)RVRHA-DTG(K-DABCYL)IFAAISLFSLLFQPLFGLSKKR-nh2), KSp64 (KRHRI(K-TAMRA)RVRHA-DTG(C-QXL610)IFAAISLFSLLFQPLFGLSKKR-nh2) and KSp96 (KRHRINRVR(E-EDANS)A-DTG(K-DABCYL)IFAAISLFSLLFQPLFGLSKKR-nh2) were synthesised and purified as described (Ticha, et al., 2017). N-substituted peptidyl-α-ketoamides were synthesised in four stages using published methods: (1) isocyanides corresponding to the tail modification were synthesised by Hoffman isocyanide synthesis (Cao, et al., 2010), (2) the Boc-protected P1 amino acid was converted to an aminoaldehyde and then to the respective α-hydroxy-β-aminoamide by the Passerini reaction with the corresponding isocyanide (Semple, et al., 2000; Venkatraman, et al., 2006), (3) the peptide component corresponding to the P5 to P2 segment of the inhibitor was synthesised on solid phase using the 2-chlorotrityl resin, and (4) the deprotected α-hydroxy-β-aminoamide hydrochloride was coupled to the P5-P2 peptide (Coste, et al., 1994) with consequent Dess-Martin oxidation and removal of the orthogonal protection groups (Souček and Urban, 1995; Yin, et al., 2007) to yield the final N-substituted peptidyl-α-ketoamides. The unsubstituted ketoamide has been synthesized by transforming the aldehyde moiety of the Boc-protected P1 aminoaldehyde formed in step (2) to a cyanhydrin and subsequent partial hydrolysis of the cyanide moiety to an amide (Venkatraman, et al., 2006).

Peptidyl chloromethylketones were synthesised by the mixed anhydride method as described (Zoll, et al., 2014). Peptidyl acylsufonamides were synthesised by transformation of the N-protected alanine to the protected N-acylsulfonamides as described (D'Andrea and Scola, 2008) and coupling of the deprotected N-acylsulfonamide to the peptides (Coste et al., 1994). Peptidyl trifluoromethylketones were synthesized by coupling of the peptide with 3-amino-1,1,1-trifluorobutan-2-ol hydrochloride by TBTU amide coupling (Bastiaans, et al., 1997) and subsequent Dess-Martin oxidation as described (Souček and Urban, 1995; Yin, et al., 2007). Peptidyl thiazolylketones were prepared by coupling of N-Boc-alanine to 2-bromothiazol as described (Dondoni and Perrone, 1993). Consequent deprotection and coupling to Ac-RVRH-OH was done using TCFH (tetramethylchloroformamidinium hexafluorophosphate) as reported (Tulla-Puche, et al., 2008). Peptidyl boronates were synthesised by coupling of (R)-Boroalanine-(1S,2S,3R,5S)-(+)-2,3-pinanediol ester hydrochloride to the protected Ac-RVRH-OH by the mixed anhydride method as described (Zoll, et al., 2014).

All compounds were characterised by mass spectrometry and NMR as documented in the next section. Analytical reversed-phase HPLC was run on a Reprosil-100, C18, 250×4.6 mm, 5 µm column (Watrex International, Inc, San Francisco, CA, USA) using gradients of acetonitrile (solvent B) and 0.1% (v/v) aqueous trifluoroacetic acid (solvent A), flow rate of 1 mL/min and UV detection at 210 nm. Preparative HPLC separations were performed on a YMC-Pack, ODS-AM, 250×20 mm, 5 µm column (YMC America, Inc., Allentown, PA, U.S.A.) using gradients of acetonitrile and 0.1% (v/v) aqueous trifluoroacetic acid (TFA) with UV detection at 210 – 235 nm at a flow rate of 10 mL/min. Mass-spectra were acquired on Waters Micromass ZQ ESCi multimode ionization mass-spectrometer and LTQ Orbitrap XL (Thermo Fisher Scientific) using ESI (+) ionization. LC-MS analysis was performed on Agilent Technologies Liquid Chromatograph coupled with TOF 6230 ESI-MS detector, using gradient of 2 to 100% solvent B over 10 min on a Waters 1.7 µm C18 , 100×2.1 mm column at a flow rate of 0.3 mL/min, where solvent A = 0.1% formic acid/water and solvent B = 0.1% formic acid/acetonitrile). NMR spectra were acquired on a Bruker AV-400 MHz spectrometer at room temperature.

Compound Characterization Data

<u>Peptidyl chlormethylketones</u> (Zoll, et al., 2014)

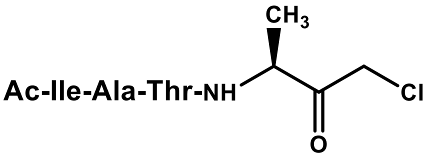

HPLC-analysis: Gradient 15-50 % B over 30 min; One peak at 13.3 min.

HR-MS: Monoisotopic mass 449.21672 calculated for $[M+H]^+$; found 449.21628.

^{1}H NMR (400 MHz, DMSO-d_6) δ (ppm) = 0.86 – 0.71(m, 6H, CH3), 1.04 (d, 3H, CH3, 3J = 6.4 Hz), 1.13 – 1.06 (m, 2H, CH2), 1.22 (d, 6H, CH3, J = 7.1 Hz), 1.47 – 1.35 (m, 1H, CH), 1.85 (s, 3H, acetyl), 4.06 – 3.93 (m, 1H, CH), 4.21 – 4.10 (m, 2H, CH), 4.40 – 4.28 (m, 2H, CH), 4.65 – 4.52 (m, 2H, COCH2Cl), 4.94 (s, 1H, OH), 7.6 (d, 1H, CONH, J = 7.9 Hz), 7.93 (d, 1H, CONH, J= 8.2 Hz), 8.23 (dd, 2H, CONH, J = 24.3, 6.9 Hz).

^{13}C NMR (101 MHz, DMSO-d_6) δ (ppm) = 201.17, 172.25, 171.12, 170.24, 169.33, 66.47, 58.04, 56.80, 52.37, 48.35, 47.45, 36.48, 24.36, 22.44, 19.78, 17.60, 15.70, 15.34, 10.98.

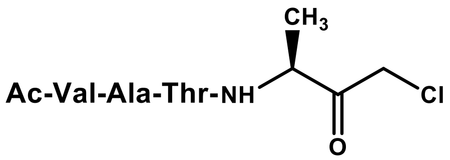

HPLC analysis: Gradient 2 – 30 % B over 30 min; One peak at 19.55 min.

ESI-MS: Monoisotopic mass: 434.2, calculated for $C_{18}H_{31}ClN_4O_6$; found $[M+H]^+$ 435.2, $[M+Na]^+$ 457.3.

^{1}H NMR (400 MHz, DMSO-d_6) δ (ppm) = 8.25 (d, J = 6.6 Hz, 1H, CONH), 8.17 (d, J = 7.1 Hz, 1H, CONH), 7.90 (d, J = 8.6 Hz, 1H, CONH), 7.59 (d, J = 8.2 Hz, 1H, CONH), 4.65 – 4.52 (m, 2H,COCH2Cl), 4.34 (td, J = 7.0, 3.9 Hz, 2H, CH), 4.19 – 4.08 (m, 2H, CH), 4.00 (dd, J = 6.3, 4.0 Hz, 1H, CH), 1.98 – 1.9 (m, 1H, CH), 1.86 (s, 3H, acetyl), 1.22 (d, J = 7.1 Hz, 6H, CH3), 1.04 (d, J = 6.3 Hz, 3H, CH3), 0.84 (dd, J = 8.2, 6.9 Hz, 6H, CH3).

^{13}C NMR (101 MHz, DMSO-d_6) δ (ppm) = 201.21, 172.32, 171.09, 170.27, 169.45, 66.50, 58.06, 57.68, 52.39, 48.33, 47.49, 30.37, 22.46, 19.83, 19.25, 18.19, 17.63, 15.73.

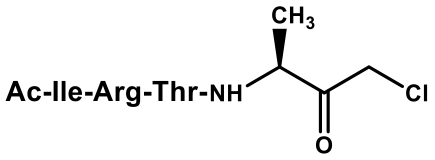

LC-MS analysis: One peak at 6.75 min, corresponding to the molecular mass $[M+H]^+$ 534.279.

HR-MS: Monoisotopic mass: 533.27286, calculated for $C_{22}H_{40}ClN_7O_6$, found $[M+H]^+$ 534.2804.

^{1}H NMR (400 MHz, DMSO-d_6) δ (ppm) = 8.33 (d, *J* = 6.5 Hz, 1H, CONH), 8.17 (d, *J* = 8.3 Hz, 1H, CONH), 7.96 (d, *J* = 8.6 Hz, 1H, CONH), 7.67 (d, *J* = 8.3 Hz, 1H, CONH), 7.53 – 7.44 (m, 1H, CONH), 4.97 (s, 1H, OH), 4.66 – 4.53 (m, 2H, $COCH_2Cl$), 4.39 – 4.26 (m, 2H, CH), 4.22 – 4.11 (m, 2H, CH), 4.06 – 3.95 (m, 1H, CH), 3.16 – 3.03 (m, 2H, CH_2), 1.85 (s, 3H, acetyl), 1.78 – 1.60 (m, 2H, CH_2), 1.60 – 1.36 (m, 5H, CH_2+CH), 1.22 (d, *J* = 7.1 Hz, 3H, CH_3), 1.04 (d, *J* = 6.3 Hz, 3H, CH_3), 0.86 – 0.70 (m, 7H, CH_3).

^{13}C NMR (101 MHz, DMSO-d_6) δ (ppm) = 201.27, 171.47, 170.26, 169.51, 156.64, 66.58, 57.99, 57.06, 52.49, 47.49, 36.34, 28.73, 25.03, 24.45, 22.44, 19.83, 15.71, 15.39, 10.97.

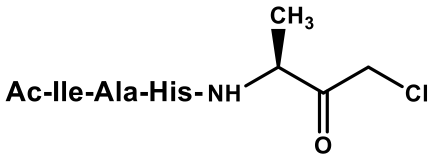

HPLC analysis: Two peaks at 6.84 and 6.94 min, both corresponding to the molecular mass $[M+H]^+$ 485.25 (diastereoisomers).

HR-MS: Monoisotopic mass 484.220096, calculated for $C_{21}H_{33}ClN_6O_5$, found $[M+H]^+$ 485.22729.

^{1}H NMR (400 MHz, DMSO-d_6) δ (ppm) = 14.15 (s, 1H, NH imidazole), 8.98 (dd, *J* = 4.3, 1.3 Hz, 1H, aromatic), 8.41 (d, *J* = 6.8 Hz, 1H, CONH), 8.34 – 8.24 (m, 1H, CONH), 8.19 (dd, *J* = 11.3, 7.0 Hz, 1H, CONH), 7.90 (dd, *J* = 8.3, 2.0 Hz, 1H, CONH), 7.36 (d, *J* = 11.3 Hz, 1H, aromatic), 4.60 (d, *J* = 2.5 Hz, 1H, $COCH_2Cl$), 4.56 (d, *J* = 5.5 Hz, 1H, $COCH_2Cl$), 4.53 – 4.48 (m, 1H, CH), 4.43 – 4.31 (m, 1H, CH), 4.22 – 4.04 (m, 2H, CH), 3.19 – 3.05 (m, 1H, CH_2), 3.02 – 2.90 (m, 1H, CH_2), 1.86 (d, *J* = 2.9 Hz, 3H, acetyl), 1.71 – 1.61 (m, 1H, CH), 1.44 – 1.36 (m, 1H, CH_2), 1.33 (s, 1H, CH_2), 1.25 – 1.15 (m, 5H, CH_3), 1.13 (d, *J* = 7.1 Hz, 2H, CH_3), 0.84 – 0.76 (m, 7H, CH_3).

^{13}C NMR (101 MHz, DMSO-d_6) δ (ppm) = 200.98, 172.42, 171.62, 170.20, 133.72, 130.17, 129.32, 116.89, 56.95, 53.58, 52.31, 51.44, 47.45, 36.25, 24.46, 22.50, 17.50, 17.23, 15.62, 15.26, 10.90.

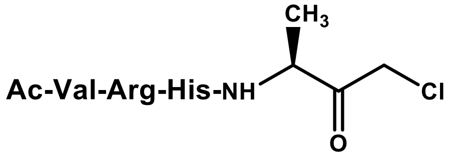

HPLC analysis: Gradient 2 – 30 % B over 30 min; One peak at 17.00 min.

ESI-MS: Monoisotopic mass: 555.27, calculated for $C_{23}H_{38}ClN_9O_5$; found $[M+H]^+$ 556.3, $[M+Na]^+$ 578.4.

^{1}H NMR (400 MHz, DMSO-d_6) δ (ppm) = 0.82 (d, *J* = 6.8 Hz, 6H, CH_3), 1.19 (d, *J* = 7.1 Hz, 3H), 1.58 – 1.34 (m, 3H, CH_2), 1.72 – 1.58 (m, 1H, CH_2), 1.87 (s, 3H, acetyl), 1.96 – 1.88 (m, 1H, CH_2), 2.95 (dd, *J* = 15.1, 7.5 Hz, 1H, CH_2), 3.17 – 3.01 (m, 3H, CH_2, CH), 4.14 – 4.02 (m, 1H, CH), 4.21 (q, *J* = 6.9 Hz, 1H, CH), 4.32 (q, *J* = 7.1 Hz, 1H, CH), 4.64 – 4.45 (m, 3H, $COCH_2Cl$ + CH), 7.35 (s, 1H, imidazole), 7.52 (t, *J* = 5.6 Hz, 1H, imidazole), 7.91 (d, *J* = 8.2 Hz, 1H, CONH), 8.12 (d, *J* = 7.6 Hz, 1H, CONH), 8.28 (d, *J* = 7.7 Hz, 1H, CONH), 8.49 (d, *J* = 6.5 Hz, 1H, CONH), 8.95 (s, 1H, NH), 14.15 (s, 1H, NH imidazole).

^{13}C NMR (101 MHz, DMSO-d_6) δ (ppm) = 200.79, 171.49, 169.91, 156.63, 135.99, 133.77, 58.07, 52.25, 51.41, 47.36, 30.07, 29.56, 28.59, 26.12, 25.02, 22.45, 19.13, 18.32, 15.54.

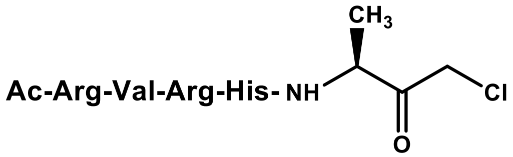

HPLC analysis: Gradient 2 – 30 % B over 30 min; One peak at 16.40 min.

HR-MS: Monoisotopic mass: 711.36955 calculated for $C_{29}H_{50}ClN_{13}O_6$; found $[M+H]^+$ 712.37716.

^{1}H NMR (400 MHz, DMSO-d_6) δ(ppm) = 0.80 (dd, *J* = 11.2, 6.8 Hz, 6H, CH_3), 1.19 (d, *J* = 7.1 Hz, 3H, CH_3), 1.57 – 1.36 (m, 6H, CH_2), 1.73 – 1.56 (m, 2H, CH_2), 1.87 (s, 3H, acetyl), 2.02 – 1.89 (m, 1H, CH), 2.95 (dd, *J* = 15.3, 8.0 Hz, 1H, CH), 3.08 (d, *J* = 4.6 Hz, 5H, CH_2, CH), 4.39 – 4.13 (m, 4H, CH), 4.61 – 4.48 (m, 3H, $COCH_2Cl$, CH), 7.34 (s, 1H, CH-imidazole), 7.74 – 7.55 (m, 3H, CONH + NH), 8.16 (d, *J* = 7.8 Hz, 2H, CONH), 8.34 (d, *J* = 7.5 Hz, 1H, CONH), 8.51 (d, *J* = 6.6 Hz, 1H, CONH), 8.94 (s, 1H, CH-imidazole), 14.26 (s, 1H).

^{13}C NMR (101 MHz, DMSO-d_6) δ (ppm) = 200.79, 171.44, 170.97, 169.94, 169.69, 156.72, 133.74, 129.11, 116.77, 57.21, 52.26, 51.42, 47.34, 30.71, 28.71, 25.03, 22.44, 21.57, 19.08, 17.91, 15.52.

Peptidyl sulfonamide

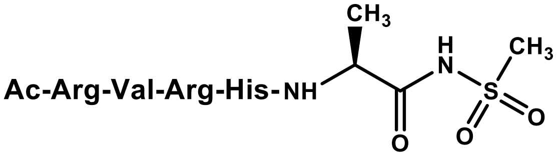

LC-MS analysis: One major peak at 1.47 min, corresponding to the mass $[M+H]^+$ 757.39.

HR-MS: Monoisotopic mass 756.381326 calculated for $C_{29}H_{52}N_{14}O_8S$, found $[M+H]^+$ 757.38868.

^{1}H NMR (401 MHz, DMSO-d_6) δ (ppm) = 8.95 (s, 1H, aromatic), 8.35 (d, *J* = 6.4 Hz, 1H CONH), 8.25 (d, *J* = 7.8 Hz, 1H, CONH), 8.15 (d, *J* = 7.8 Hz, 2H, CONH), 7.61 (t, *J* = 8.8 Hz, 3H, CONH), 7.32 (s, 1H, aromatic), 4.59 (q, *J* = 7.5 Hz, 1H, CH), 4.31 – 4.13 (m, 4H, CH), 3.21 (s, 3H, CH_3), 3.08 (d, *J* = 5.6 Hz, 5H, CH + CH_2), 2.95 (dd, *J* = 15.3, 7.8 Hz, 1H, CH), 2.00 – 1.89 (m, 1H, CH), 1.87 (s, 3H, $COCH_3$), 1.73 – 1.58 (m, 2H, CH_2), 1.57 – 1.35 (m, 6H, CH_2), 1.26 (d, *J* = 7.2 Hz, 3H, CH_3), 0.79 (dd, *J* = 11.7, 6.8 Hz, 6H, CH_3).

^{13}C NMR (101 MHz, DMSO-d_6) δ (ppm) = 171.52, 169.71, 158.56, 158.24, 156.71, 133.71, 124.59, 116.79, 115.53, 57.16, 52.42, 51.13, 48.92, 40.90, 40.42, 30.78, 28.73, 25.14, 22.47, 19.11, 17.90, 16.95.

Peptidyl thiazolylketone

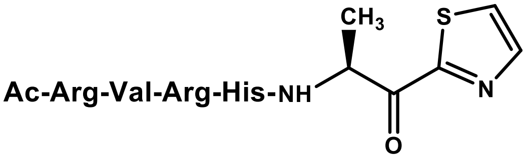

Peak 1 (used in this study)

HPLC analysis: Gradient 2-30 % B over 30 min. One peak at 19.23 min; ESI-MS $[M+H]^+$ 747.4.

HR-MS: Molecular mass 746.37588, calculated for $C_{31}H_{50}N_{14}O_6S$; found $[M+H]^+$ 747.38341.

^1H NMR (400 MHz, DMSO-d_6) δ (ppm) = 14.28 (s, 1H, NH imidazole), 9.08 (s, 1H, aromatic), 8.69 (d, J = 6.2 Hz, 1H, CONH), 8.44 – 8.35 (m, 2H, CONH), 8.35 – 8.21 (m, 3H, CONH+aromatic), 7.68 (dt, J = 13.0, 7.1 Hz, 3H, aromatic), 7.47 (d, J = 11.9 Hz, 1H, aromatic), 5.52 – 5.40 (m, 1H, CH), 4.76 (q, J = 7.4 Hz, 1H, CH), 4.43 – 4.24 (m, 3H, CH), 3.19 (dd, J = 12.2, 6.1 Hz, 5H, CH_2), 3.00 (s, 1H, CH_2), 2.04 (dd, J = 13.1, 6.4 Hz, 1H, CH), 1.97 (s, 3H, $COCH_3$), 1.84 – 1.52 (m, 8H, CH_2), 1.54 – 1.46 (m, 3H, CH_3), 0.90 (dd, J = 11.9, 6.7 Hz, 6H, CH_3).

Peak 2

HPLC analysis: Gradient 2-30 % B over 30 min. One peak at 20.01 min.

ESI-MS: $[M+H]^+$ 747.4.

HR-MS: Molecular mass 746.37588, calculated for $C_{31}H_{50}N_{14}O_6S$; found $[M+H]^+$ 747.38330.

^1H NMR (400 MHz, DMSO-d_6) δ (ppm) = 8.95 (s, 1H, aromatic), 8.46 (d, J = 6.1 Hz, 1H, CONH), 8.41 (d, J = 8.5 Hz, 1H, CONH), 8.34 (d, J = 6.3 Hz, 1H, CONH), 8.30 (d, J = 3.0 Hz, 1H, aromatic), 8.26 (d, J = 6.2 Hz, 1H, CONH), 8.21 (d, J = 3.0 Hz, 1H, CONH), 8.17 (s, 1H, aromatic), 7.65 – 7.52 (m, 3H, aromatic+NH_2), 7.33 (d, J = 5.7 Hz, 1H, aromatic), 5.45 – 5.30 (m, 1H, CH), 4.76 – 4.57 (m, 1H, CH), 4.30 – 4.05 (m, 3H, CH), 3.20 – 2.98 (m, 5H, CH_2), 2.91 (dd, J = 14.9, 9.3 Hz, 1H, CH_2), 1.90 (dt, J = 13.1, 6.5 Hz, 1H, CH), 1.84 (d, J = 5.0 Hz, 3H, $COCH_3$), 1.72 – 1.61 (m, 1H), 1.60 – 1.37 (m, 8H, CH_2), 0.80 (dd, J = 11.8, 6.8 Hz, 6H, CH_3).

^{13}C NMR (101 MHz, DMSO-d_6) δ (ppm) = 197.29, 194.51, 171.55, 169.31, 167.53, 158.06, 156.66, 133.84, 128.59, 107.86, 104.97, 63.92, 57.16, 50.62, 50.26, 48.84, 30.77, 25.12, 22.40, 19.04, 17.98, 16.72.

<u>Peptidyl trifluoromethylketone</u>

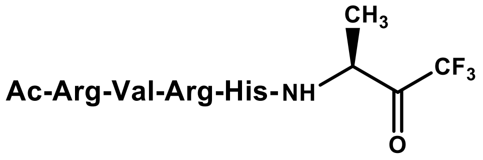

LC-MS analysis: Two peaks at 1.73 and 1.94 min, corresponding to the $[M+H]^+$ 732.39 (diastereomers). Peak 1 used in this study.

HR-MS: Molecular mass 731.38028, calculated for $C_{29}H_{48}F_3N_{13}O_6$, found $[M+H]^+$ 732.38776 and $[M+Na]^+$ 754.36969.

^1H NMR (401 MHz, DMSO-d_6) δ (ppm) = 14.22 (s, 2H, NH imidazole), 8.97 (s, 1H, aromatic), 8.28 (d, J = 8.0 Hz, 1H, CONH), 8.21 (d, J = 8.2 Hz, 1H, CONH), 8.19-8.10 (s, 2H, CONH), 7.82 (d, J = 9.3 Hz, 1H, CONH), 7.69 (d, J = 9.5 Hz, 1H, CONH), 7.64 – 7.50 (m, 3H, NH+NH_2), 7.36 (s, 1H, aromatic), 7.28 (s, 1H, NH), 7.06 (s, 2H, NH_2), 4.69 – 4.58 (m, 1H, CH), 4.33 – 4.07 (m, 4H, CH), 3.05 (ddt, J = 20.0, 14.9, 7.4 Hz, 5H, CH+CH_2), 2.92 – 2.82 (m, 1H, CH), 2.00 - 1.90 (m, 1H, CH), 1.87 (s, 3H, $COCH_3$), 1.63 (d, J =

5.4 Hz, 2H, CH_2), 1.46 (dt, *J* = 19.0, 10.3 Hz, 6H, CH_2), 1.08 (d, *J* = 6.8 Hz, CH_3), 1.00 (d, *J* = 6.8 Hz, CH_3), 0.78 (ddd, *J* = 11.1, 6.7, 2.5 Hz, 6H, CH_3).

^{13}C NMR (101 MHz, DMSO-d_6) δ (ppm) = 207.88, 172.52, 171.94, 169.77, 166.63, 158.63, 157.16, 152.24, 117.20, 113.16, 57.57, 52.85, 31.29, 27.23, 25.40, 22.94, 19.58, 18.35.

Peptidyl boronate

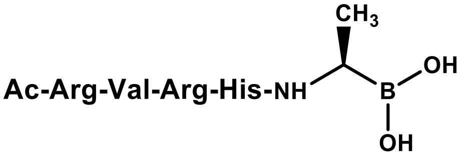

Peak 1

HPLC analysis: Gradient 2-30 % B over 30 min; One peak at 13.43 min

ESI-MS: Monoisotopic mass: 679.40, calculated for $C_{27}H_{50}BN_{13}O_7$, found $[M+H]^+$ 680.1, (diastereomer).

^1H NMR (401 MHz, DMSO-d_6) δ (ppm) = 14.20 (s, 1H, NH imidazole), 8.95 (s, 1H, aromatic), 8.18 (dd, *J* = 24.1, 7.7 Hz, 3H, CONH), 7.92 (t, *J* = 5.5 Hz, 1H, CONH), 7.60 (dt, *J* = 10.4, 5.0 Hz, 3H, NH_2+NH), 7.33 (d, *J* = 5.4 Hz, 2H, aromatic+NH), 4.48 (dq, *J* = 14.4, 7.6 Hz, 1H, CH), 4.30 – 4.23 (m, 1H, CH), 4.19 (q, *J* = 9.5, 8.2 Hz, 2H, CH), 3.06 (tt, *J* = 16.0, 7.1 Hz, 6H, CH_2+CH), 2.93 (dq, *J* = 15.0, 7.6 Hz, 2H,CH_2), 1.96 (dd, *J* = 13.5, 6.7 Hz, 1H, CH), 1.87 (s, 3H, $COCH_3$), 1.74 – 1.58 (m, 2H, CH_2), 1.56 – 1.35 (m, 6H, CH_2), 1.03 (d, *J* = 7.6 Hz, 1H, CH_3), 0.95 (t, *J* = 7.2 Hz, 2H, CH_3), 0.80 (ddd, *J* = 11.5, 6.7, 2.4 Hz, 6H, CH_3).

Peak 2 (used in this study)

HPLC analysis: Gradient 2-30 % B over 30 min; One peak at 14.12 min

ESI-MS: Monoisotopic mass: 679.40, calculated for $C_{27}H_{50}BN_{13}O_7$, found $[M+H]^+$ 680.1, (diastereomer).

^1H NMR (401 MHz, DMSO-d_6) δ (ppm) = 8.93 (s, 1H, aromatic), 8.40 (d, *J* = 8.5 Hz, 1H, CONH), 8.29 (d, *J* = 6.1 Hz, 1H, CONH), 8.18 (dt, *J* = 13.4, 6.9 Hz, 2H, CONH), 7.85 (t, *J* = 5.5 Hz, 1H, CONH), 7.60 (d, *J* = 7.4 Hz, 2H, NH_2), 7.32 (d, *J* = 7.7 Hz, 1H, aromatic), 4.61 – 4.45 (m, 1H, CH), 4.37 – 4.13 (m, 3H, CH), 4.14 – 4.03 (m, 1H, CH), 3.22 – 3.01 (m, 6H, CH_2), 3.00 – 2.82 (m, 2H, CH_2), 2.02 – 1.90 (m, 1H, CH), 1.88 (s, 3H, $COCH_3$), 1.75 – 1.58 (m, 2H, CH_2), 1.49 (dt, *J* = 17.7, 8.1 Hz, 6H, CH_2), 1.27 – 1.09 (m, 1H, CH_3), 1.07 – 0.91 (m, 2H, CH_3), 0.88 – 0.69 (m, 6H, CH_3).

Peptidyl ketoamides

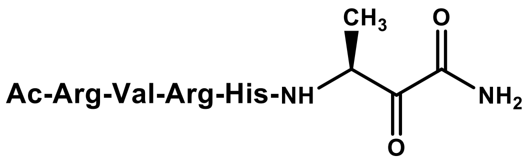

Compound ***1***

LC-MS analysis: Gradient 2 – 30 % B over 7 min; Three diastereomeric peaks at 1.85, 2.03 and 2.2 min, corresponding to $[M+H]^+$ 707.4.

HR-MS: Monoisotopic mass: 706.40, calculated for $C_{29}H_{50}N_{14}O_7$; found $[M+H]^+$ 707.40638 and $[M+2H]^{2+}$ 354.20689.

Insufficient amount for quality NMR.

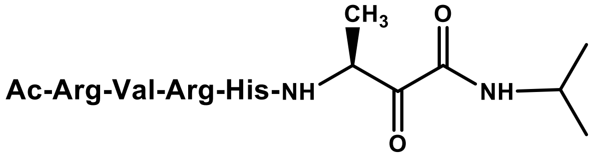

Compound ***2***

HPLC analysis: Gradient 2 – 100 % B over 36 min.; One peak at 13.45 min.

ESI-MS: $[M+H]^+$ 749.6, $[M+Na]^+$ 771.6, $[M+2H]^{2+}$ 375.3.

HR-MS: Monoisotopic mass: 748.45, calculated for $C_{32}H_{56}N_{14}O_7$; found $[M+H]^+$ 749.45324, $[M+Na]^+$ 771.43518 and $[M+2H]^{2+}$ 375.23028.

^{1}H NMR (401 MHz, DMSO-d_6) δ (ppm) = 8.93 (s, 1H, COCONH), 8.53 (d, *J* = 8.3 Hz, 1H, aromatic), 8.43 (d, *J* = 6.3 Hz, 1H, CONH), 8.25 (d, *J* = 7.9 Hz, 1H, CONH), 8.21 – 8.11 (m, 2H, CONH), 7.74 – 7.58 (m, 3H, CONH + NH_2), 7.33 (s, 1H, aromatic), 5.13 – 4.93 (m, 1H, CH), 4.69 – 4.56 (m, 1H, CH), 4.36 – 4.13 (m, 3H, CH), 3.93 (dt, *J* = 14.8, 7.3 Hz, 1H, CH), 3.09 (d, *J* = 5.6 Hz, 5H, CH+ CH_2), 3.00 – 2.85 (m, 1H, CH), 1.97 (dd, *J* = 15.0, 8.3 Hz, 1H, CH), 1.88 (s, 3H, $COCH_3$), 1.73 – 1.58 (m, 2H, CH_2), 1.49 (dt, *J* = 15.2, 9.0 Hz, 6H, CH_2), 1.26 (d, *J* = 7.3 Hz, 3H, CH_3), 1.11 (d, *J* = 6.6 Hz, 6H, CH_3), 0.80 (dd, *J* = 11.5, 6.7 Hz, 6H, CH_3).

^{13}C NMR (101 MHz, DMSO-d_6) δ (ppm) = 187.06, 171.49, 171.27, 159.60, 156.72, 135.61, 133.76, 115.70, 104.52, 57.14, 52.38, 49.54, 30.78, 28.71, 25.07, 22.46, 21.79, 19.10, 17.88, 15.61.

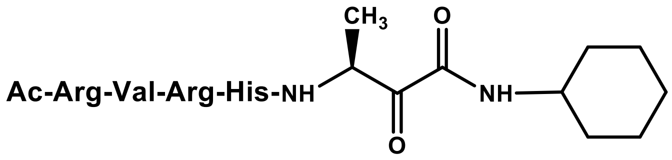

Peak 1 (compound ***3***)

LC - MS analysis: One peak at 5.87 min with the corresponding mass of the product $[M+H]^+$ 789.48.

HR-MS: Monoisotopic mass: 788.4769 calculated for $C_{35}H_{60}N_{14}O_7$ found: $[M+2H]^{2+}$ 395.24606 and $[M+3H]^{3+}$ 263.83329.

^{1}H NMR (401 MHz, DMSO-d_6) δ (ppm) = 14.30 (s, 2H, NH), 9.03 – 8.93 (m, 1H, CONH), 8.55 – 8.47 (m, 1H, aromatic), 8.42 (t, *J* = 6.4 Hz, 1H, CONH), 8.25 (d, *J* = 8.1 Hz, 1H, CONH), 8.16 (q, *J* = 6.7 Hz, 2H, CONH), 7.64 (dtd, *J* = 14.0, 8.7, 4.9 Hz, 3H, CONH), 7.39 – 7.31 (m, 1H, aromatic), 5.02 (tt, *J* = 9.7, 5.8 Hz, 1H, CH), 4.62 (td, *J* = 8.1, 5.6 Hz, 1H, CH), 4.31 – 4.22 (m, 1H, CH), 4.19 (dtd, *J* = 11.2, 6.2, 2.9 Hz, 2H, CH), 3.57 (dt, *J* = 19.8, 6.2 Hz, 1H, CH), 3.07 (q, *J* = 6.4 Hz, 5H, CH_2), 2.96 – 2.83 (m, 1H, CH_2 His), 2.02 – 1.89 (m, 1H, CH), 1.87 (d, *J* = 2.2 Hz, 3H, acetyl), 1.74 – 1.58 (m, 6H, CH_2), 1.49 (dtd, *J* = 25.9, 15.5, 14.7, 6.3 Hz, 7H, CH_2), 1.26 (dq, *J* = 14.3, 11.4, 10.8 Hz, 7H), 0.79 (dd, *J* = 11.4, 6.8 Hz, 6H, CH_3).

^{13}C NMR (101 MHz, DMSO-d_6) δ (ppm) = 171.49, 171.28, 169.62, 158.58 , 158.26 , 156.73 , 133.72, 129.06, 115.37, 57.16, 52.40, 49.59, 48.07, 31.78, 30.78, 28.75, 25.07, 24.73, 22.46, 19.10, 17.89, 15.60.

Peak 2

LC-MS analysis: One peak at 6.18 min with the corresponding mass of the product $[M+H]^+$ 789.48.

HR-MS: Monoisotopic mass: 788.4769 calculated for $C_{35}H_{60}N_{14}O_7$ found: $[M+2H]^{2+}$ 395.24567 and $[M+3H]^{3+}$ 263.83298.

^{1}H NMR (401 MHz, DMSO-d_6) δ (ppm) = 14.23 (s, 1H, NH), 9.05 – 8.93 (m, 1H, CONH), 8.51 (d, *J* = 8.3 Hz, 1H, aromatic), 8.40 (q, *J* = 9.3, 7.9 Hz, 1H, CONH), 8.30 – 8.11 (m, 3H, CONH), 7.67 – 7.53 (m, 2H, CONH), 7.35 (d, *J* = 4.0 Hz, 1H, aromatic), 5.07 – 4.97 (m, 1H, CH), 4.70 – 4.56 (m, 1H, CH), 4.25 (qd, *J* = 9.8, 8.4, 3.8 Hz, 1H, CH), 4.22 – 4.05 (m, 2H, CH), 3.65 – 3.50 (m, 1H, CH), 3.18 – 2.98 (m, 5H, CH_2), 2.90 (td, *J* = 14.7, 13.5, 7.3 Hz, 1H, CH_2), 1.87 (dd, *J* = 6.1, 2.0 Hz, 3H, acetyl), 1.76 – 1.62 (m, 5H, CH_2), 1.63 – 1.39 (m, 7H, CH_2), 1.39 – 1.15 (m, 8H, CH_2, CH_3), 1.16 -1.00 (m, 2H, CH_2), 0.80 (ddd, *J* = 11.7, 7.1, 4.6 Hz, 6H, CH_3).

^{13}C NMR (101 MHz, DMSO-d_6) δ (ppm) = 195.26, 188.02, 158.45, 156.68, 140.04, 115.29, 64.61, 48.07, 46.50, 43.74, 31.78, 30.72, 28.43, 26.91, 24.74, 22.45, 19.06, 18.00.

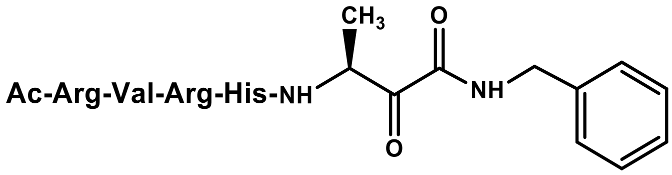

Peak 1

LC-MS analysis: One peak at 5.81 min with the corresponding mass of the product $[M+H]^+$ 797.45.

HR-MS: Monoisotopic mass: 796.4456 calculated for $C_{36}H_{56}N_{14}O_7$ found: $[M+H]^+$ 797.45301.

^{1}H NMR (401 MHz, DMSO-d_6) δ 14.27 (s, 1H, NH imidazole), 8.94 (d, J = 8.1 Hz, 1H, aromatic), 8.55 – 8.39 (m, 1H, CONH), 8.24 (t, J = 7.3 Hz, 1H, CONH), 8.21 – 8.12 (m, 1H, CONH), 7.75 – 7.55 (m, 3H, CONH), 7.49 – 7.15 (m, 8H, aromatic+NH_2), 5.08 – 4.97 (m, 1H, CH), 4.69 – 4.55 (m, 1H, CH), 4.41 – 4.06 (m, 5H, CH + CH_2), 3.19 (dd, J = 15.3, 5.2 Hz, 1H, CH_2 His), 3.06 (dq, J = 12.7, 6.2 Hz, 4H, CH_2), 2.92 (dd, J = 15.2, 9.0 Hz, 1H, CH_2 His), 1.98 – 1.80 (m, 4H, + CH), 1.73 – 1.57 (m, 2H, CH_2), 1.47 (dt, J = 15.9, 8.0 Hz, 5H, CH_2), 1.37 – 1.14 (m, 3H, CH_3), 0.87 – 0.69 (m, 6H, CH_3).

Peak 2 (compound ***4*****)**

LC-MS analysis: One peak at 6.07 min with the corresponding mass of the product $[M+H]^+$ 797.45.

HR-MS: Monoisotopic mass: 796.4456 calculated for $C_{36}H_{56}N_{14}O_7$ found: $[M+H]^+$ 797.45282.

Yield: 1.3 mg (4.2%).

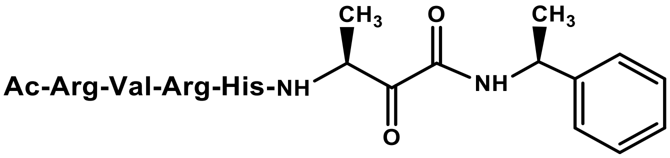

Peak 1 (compound ***5***)

HPLC analysis: One peak at 14.2 min Gradient 2 – 100 % B over 36 min.

ESI-MS: $[M+H]^+$ 811.5.

HR-MS: Monoisotopic mass: 810.46129, calculated for $C_{37}H_{58}N_{14}O_7$ found: $[M+H]^+$ 811.46909 and $[M+2H]^{2+}$ 406.23814.

^{1}H NMR (401 MHz, DMSO-d_6) δ (ppm) = 14.30 (s, 1H, NH), 9.23 – 9.10 (m, 1H, CONH), 8.97 (s, 1H, aromatic), 8.45 (dd, J = 14.2, 6.5 Hz, 1H, CONH), 8.19 (dd, J = 31.1, 7.9 Hz, 3H, CONH), 7.61 (s, 3H), 7.34 (dt, J = 9.9, 4.3 Hz, 6H, Ar), 7.28 – 7.20 (m, 1H, CONH), 4.98 (dt, J = 15.2, 7.5 Hz, 2H, CH), 4.61 (q, J = 7.8 Hz, 1H, CH), 4.22 (dq, J = 33.3, 7.4, 5.5 Hz, 3H, CH), 3.18 – 2.98 (m, 5H, CH_2), 2.87 (dd, J = 15.7, 8.2 Hz, 1H, CH_2), 1.93 (dd, J = 12.7, 6.0 Hz, 1H, CH), 1.87 (s, 3H, acetyl), 1.72 – 1.58 (m, 2H, CH_2), 1.43 (td, J = 25.5, 22.6, 8.5 Hz, 9H, CH_2, CH_3), 1.27 (d, J = 7.3 Hz, 2H, CH_3), 1.16 (d, J = 7.1 Hz, 1H, CH_3), 0.79 (dt, J = 10.9, 5.7 Hz, 6H, CH_3).

^{13}C NMR (101 MHz, DMSO-d_6) δ (ppm) = 198.27, 173.20, 171.62, 169.73, 156.75, 139.20, 128.31, 126.18, 104.52, 67.45, 59.85, 52.41, 51.24, 48.24, 47.47, 28.75, 25.13, 22.46, 21.70, 19.09, 17.87, 15.54.

Peak 2

HPLC analysis: One peak at 14.6 min; Gradient 2 – 100 % B over 36 min.

ESI-MS: $[M+H]^+$ 811.4.

HR-MS: Monoisotopic mass: 810.46129, calculated for $C_{37}H_{58}N_{14}O_7$ found: $[M+H]^+$ 811.46901 and $[M+2H]^{2+}$ 406.23809.

^{1}H NMR (401 MHz, DMSO-d_6) δ (ppm) = 9.20 – 9.11 (m, 1H, CONH), 8.93 (s, 1H, aromatic), 8.51 – 8.30 (m, 1H, CONH), 8.31 – 8.11 (m, 3H, CONH), 7.62 (s, 3H, NH_2), 7.47 – 7.28 (m, 6H, aromatic), 7.28 – 7.18 (m, 1H, CONH), 5.04 – 4.91 (m, 1H, CH), 4.71 – 4.52 (m, 1H, CH), 4.35 – 4.06 (m, 3H, CH), 3.17 – 2.97 (m, 4H, CH_2), 2.96 – 2.80 (m, 1H, CH_2), 1.90 – 1.80 (m, 3H, acetyl), 1.73 – 1.60 (m, 2H, CH_2), 1.44 (d, J = 17.1 Hz, 9H, CH_2, CH_3), 1.26 (dd, J = 14.0, 7.3 Hz, 3H, CH_3), 0.79 (dd, J = 10.3, 6.7 Hz, 6H, CH_3).

^{13}C NMR (101 MHz, DMSO-d_6) δ (ppm) = 198.92, 170.15, 162.64, 158.45, 156.70, 138.57, 128.30, 126.91, 126.18, 118.63, 117.47, 61.95, 56.71, 53.49, 49.73, 48.18, 28.71, 25.13, 22.45, 21.66, 19.07, 17.99.

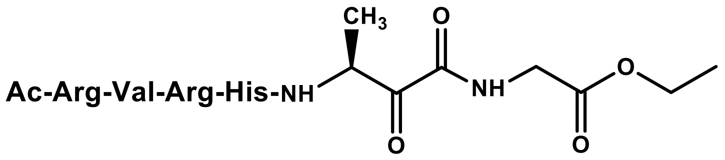

Compound ***6***

HPLC analysis: Gradient 2 – 30 % B over 30 min; One peak at 12.4 min.

ESI-MS: $[M+H]^+$ 792.4, $[M+2H]^{2+}$ 397.2.

HR-MS: Monoisotopic mass: 792.43552, calculated for $C_{33}H_{56}N_{14}O_9$; found: $[M+H]^+$ 793.44299, $[M+2H]^{2+}$ 397.22539.

^{1}H NMR (401 MHz, DMSO-d_6) δ (ppm) = 14.17 (s, 1H, NH imidazole), 9.03 (t, J = 5.9 Hz, 1H, CONH), 8.45 (d, J = 6.3 Hz, 1H, aromatic), 8.26 (d, J = 7.5 Hz, 1H, CONH), 8.15 (d, J = 7.8 Hz, 2H, CONH), 7.58 (t, J = 9.1 Hz, 2H, CONH), 7.37 – 7.26 (m, 1H, aromatic), 5.01 (p, J = 6.4, 5.8 Hz, 1H, CH), 4.61 (q, J = 8.0 Hz, 1H, CH), 4.35 – 4.05 (m, 5H, CH +CH_2), 3.90 (d, J = 6.8 Hz, 2H, CH_2), 3.19 – 2.97 (m, 5H, CH_2), 2.91 (dd, J = 15.2, 8.0 Hz, 1H, CH_2), 1.98 – 1.90 (m, 1H, CH), 1.87 (s, 3H, acetyl), 1.74 – 1.58 (m, 2H, CH_2), 1.49 (dq, J = 16.1, 7.7 Hz, 6H, CH_2), 1.31 – 1.12 (m, 6H, CH_3), 0.79 (dd, J = 11.9, 6.7 Hz, 6H, CH_3).

^{13}C NMR (101 MHz, DMSO-d_6) δ (ppm) = 168.83, 166.37, 164.30, 162.00, 156.67, 146.64, 139.66, 135.98, 134.39, 123.99, 60.73, 57.15, 53.86, 52.36, 49.54, 45.52, 28.75, 22.47, 21.18, 19.10, 14.05.

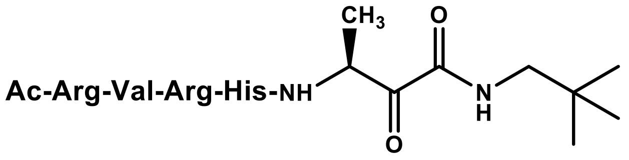

Compound **7**

LC-MS analysis: One peak at 5.66 min, corresponding to $[M+H]^+$ 777.5, $[M+2H]^{2+}$ 389.3, $[M+3H]^{3+}$ 259.85.

HR-MS: Monoisotopic mass: 776.476941, calculated for $C_{34}H_{60}N_{14}O_7$; found $[M+H]^+$ 777.48454, $[M+Na]^+$ 799.46650 and $[M+2H]^{2+}$ 389.24594.

^1H NMR (401 MHz, DMSO-d_6) δ (ppm) = 14.29 (s, 1H, NH imidazole), 8.94 (s, 1H, COCONH), 8.57 (s, 1H, aromatic), 8.42 (d, *J* = 6.4 Hz, 1H, CONH), 8.25 (d, *J* = 8.1 Hz, 1H, CONH), 8.15 (t, *J* = 7.4 Hz, 2H, CONH), 7.64 (dd, *J* = 21.6, 10.8 Hz, 2H, CONH+NH), 7.33 (s, 1H, aromatic), 5.12 – 4.94 (m, 1H,CH), 4.68 – 4.56 (m, 1H, CH), 4.35 – 4.12 (m, 3H, CH), 3.18 – 3.00 (m, 5H, CH_2), 2.96 (d, *J* = 6.8 Hz, 3H, CH_2), 2.03 – 1.90 (m, 1H, CH), 1.87 (s, 3H, $COCH_3$), 1.65 (dt, *J* = 16.3, 8.0 Hz, 2H, CH_2), 1.47 (dq, *J* = 24.5, 12.2, 10.4 Hz, 6H, CH_2), 1.23 (dd, *J* = 17.4, 7.3 Hz, 3H, CH_3), 0.94 – 0.63 (m, 15H, CH_3).

^{13}C NMR (101 MHz, DMSO-d_6) δ (ppm) = 196.92, 171.82, 171.51, 169.70, 161.17, 158.66, 156.75, 133.76, 57.16, 52.42, 51.05, 49.54, 32.62, 30.76, 28.75, 27.32, 25.14, 22.46, 19.10, 17.88, 15.46.

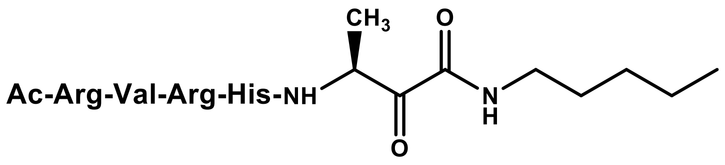

Peak 1 (compound **8**)

HPLC analysis: One peak at 14.34 min, Gradient 2 – 100 % B over 36 min.

ESI-MS: $[M+H]^+$ 777.5, $[M+Na]^+$ 799.5.

HR-MS: Monoisotopic mass: 776.476941, calculated for $C_{34}H_{60}N_{14}O_7$; found: $[M+H]^+$ 777.48440, $[M+2H]^{2+}$ 389.24586.

^1H NMR (401 MHz, DMSO-d_6) δ (ppm) = 14.20 (s, 1H, NH imidazole), 8.91 (s, 1H, CONH), 8.68 (t, *J* = 5.8 Hz, 1H, CONH), 8.41 (d, *J* = 6.4 Hz, 1H, aromatic), 8.20 (dd, *J* = 35.0, 7.4 Hz, 3H, CONH), 7.61 (d, *J* = 6.4 Hz, 3H, CONH + NH_2), 7.32 (s, 2H, NH_2), 5.02 (p, *J* = 7.2 Hz, 1H, CH), 4.61 (q, *J* = 7.8, 7.0 Hz, 1H, CH), 4.22 (dt, *J* = 31.2, 7.7 Hz, 3H, CH), 3.09 (dq, *J* = 12.1, 6.9 Hz, 7H, CH_2), 2.98 – 2.82 (m, 1H, CH_2), 2.18 – 2.03 (m, 1H, CH), 1.87 (s, 3H, acetyl), 1.64 (td, *J* = 14.1, 12.8, 7.1 Hz, 2H, CH_2), 1.58 – 1.34 (m, 8H, CH_2), 1.23 (tt, *J* = 15.5, 7.3 Hz, 7H, CH_2 + CH_3), 0.93 – 0.67 (m, 9H, CH_3).

Peak 2

HPLC analysis: One peak at 14.7 min, Gradient 2 – 100 % B over 36 min.

ESI-MS: $[M+H]^+$ 777.5, $[M+2H]^{2+}$ 389.2

HR-MS: Monoisotopic mass: 776.476941, calculated for $C_{34}H_{60}N_{14}O_7$; found: $[M+H]^+$ 777.48473, $[M+2H]^{2+}$ 389.24590.

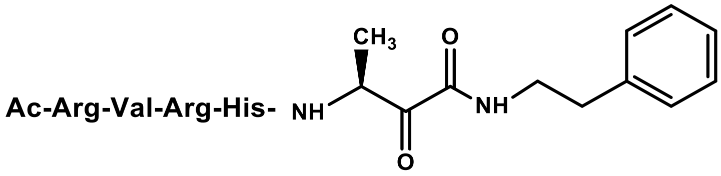

Compound ***9***

HPLC analysis: Gradient 2 – 30 % B over 30 min; One peak at 22.2 min.

ESI-MS: $[M+H]^+$ 811.3, $[M+2H]^{2+}$ 406.2

HR-MS: Monoisotopic mass 810.461290, calculated for $C_{37}H_{58}N_{14}O_7$; found: $[M+H]^+$ 811.46886, $[M+2H]^{2+}$ 406.23800.

^{1}H NMR (401 MHz, DMSO-d_6) δ (ppm) = 14.20 (s, 1H, NH imidazole), 8.93 (s, 1H, NH), 8.77 (t, *J* = 5.9 Hz, 1H, CONH), 8.41 (d, *J* = 6.2 Hz, 1H, aromatic), 8.25 (d, *J* = 7.9 Hz, 1H, CONH), 8.15 (t, *J* = 7.4 Hz, 2H, CONH), 7.62 (t, *J* = 10.9 Hz, 3H, CONH), 7.30 (dd, *J* = 14.5, 6.8 Hz, 5H, aromatic), 7.20 (d, *J* = 7.6 Hz, 5H, aromatic + NH_2), 5.01 (p, *J* = 7.2 Hz, 1H, CH), 4.62 (q, *J* = 8.0 Hz, 1H, CH), 4.34 – 4.12 (m, 3H, CH), 3.16 – 2.99 (m, 5H, CH_2), 2.91 (dd, *J* = 15.3, 8.1 Hz, 1H, CH_2), 2.77 (t, *J* = 7.5 Hz, 2H, CH_2), 2.00 – 1.90 (m, 1H, CH), 1.87 (s, 3H, acetyl), 1.73 – 1.57 (m, 2H, CH_2), 1.48 (dt, *J* = 19.0, 11.3 Hz, 6H, CH_2), 1.20 (d, *J* = 7.3 Hz, 3H, CH_3), 0.79 (dd, *J* = 11.0, 6.8 Hz, 6H, CH_3).

^{13}C NMR (101 MHz, DMSO-d_6) δ (ppm) = 196.62, 171.49, 171.28, 169.69, 169.59, 160.23, 158.33, 156.73, 138.95, 133.74, 128.60, 128.35, 126.21, 118.66, 57.16, 55.97, 52.41, 51.05, 49.45, 40.41, 34.57, 30.78, 28.74, 25.14, 22.47, 19.11, 17.89, 15.50.

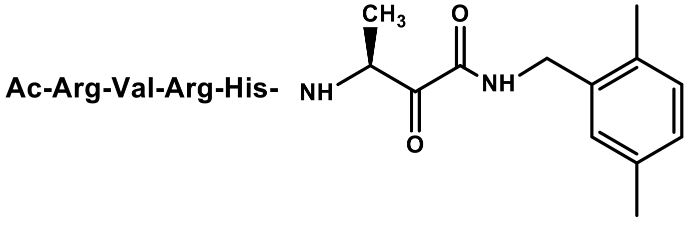

Compound ***10***

HPLC analysis: One peak at 6.35 min, corresponding to $[M+H]^+$ 825.5, $[M+2H]^{2+}$ 413.3, $[M+3H]^{3+}$ 275.84.

HR-MS: Monoisotopic mass: 824.47694, calculated for $C_{38}H_{60}N_{14}O_7$ found: $[M+H]^+$ 825.48462, $[M+2H]^{2+}$ 413.24584.

^{1}H NMR (401 MHz, DMSO-d_6) δ (ppm) = 14.25 (s, 1H, NH imidazole), 9.09 (t, J = 6.0 Hz, 1H, CONH), 8.95 (s, 1H, NH), 8.45 (d, J = 6.3 Hz, 1H, aromatic), 8.25 (d, J = 8.1 Hz, 1H, CONH), 8.15 (t, J = 6.7 Hz, 2H, CONH), 7.62 (dt, J = 13.8, 6.6 Hz, 2H, CONH), 7.33 (s, 1H, aromatic), 7.10 – 6.89 (m, 3H, aromatic), 5.04 (p, J = 7.2 Hz, 1H, CH), 4.61 (q, J = 7.9 Hz, 1H, CH), 4.33 – 4.11 (m, 5H, CH+CH_2), 3.16 – 3.00 (m, 5H, CH_2), 2.90 (dd, J = 15.1, 7.9 Hz, 1H, CH_2), 2.22 (s, 6H, CH_3),1.95 (dt, J = 13.3, 8.1 Hz, 1H, CH) , 1.87 (s, 3H, acetyl), 1.72 – 1.58 (m, 2H, CH_2), 1.57 – 1.33 (m, 6H, CH_2), 1.27 (d, J = 7.3 Hz, 3H, CH_3), 0.79 (dd, J = 11.1, 6.8 Hz, 6H, CH_3).

^{13}C NMR (101 MHz, DMSO-d_6) δ (ppm) = 200.01, 171.50, 171.30. 170.98, 169.70, 165.73, 160.58, 158.48, 156.72, 135.75, 134.51, 133.74, 129.93, 128.40, 127.59, 57.15, 52.41, 51.06, 49.58, 30.77, 28.71, 25.06, 22.46, 20.68, 19.10, 18.08, 15.53.

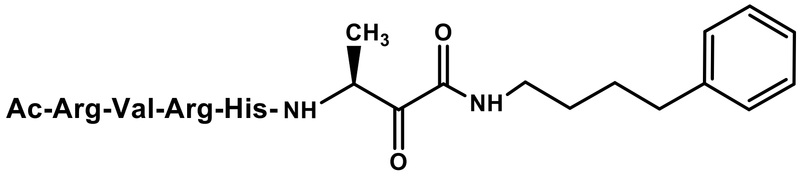

Compound ***11***

HPLC analysis: Gradient 2 – 100 % B over 36 min; One peak at 16.0 min.

ESI-MS: $[M+H]^+$ 839.5, $[M+2H]^{2+}$ 420.3.

HR-MS: Monoisotopic mass: 838.49263 calculated for $C_{39}H_{62}N_{14}O_7$; found $[M+H]^+$ 839.5007, $[M+2H]^{2+}$ 420.25366.

^{1}H NMR (401 MHz, DMSO-d_6) δ (ppm) = 14.17 (s, 1H, NH imidazole), 8.88 (s, 1H, COCONH), 8.71 (t, J = 5.8 Hz, 1H, aromatic), 8.41 (d, J = 6.4 Hz, 1H, CONH), 8.24 (d, J = 7.8 Hz, 1H, aromatic, CONH), 8.15 (d, J = 7.6 Hz, 2H, CONH), 7.59 (d, J = 6.2 Hz, 3H CONH), 7.27 (dd, J = 14.8, 7.1 Hz, 4H, aromatic), 7.18 (d, J = 7.9 Hz, 3H, aromatic), 5.08 – 4.95 (m, 1H, CH), 4.61 (q, J = 8.2, 7.0 Hz, 1H,CH), 4.22 (dt, J = 30.8, 8.0 Hz, 3H, CH), 3.11 (dd, J = 28.1, 6.1 Hz, 7H, CH_2), 2.99 – 2.84 (m, 1H, CH_2), 2.56 (d, J = 7.5 Hz, 3H, CH_2), 2.00 – 1.89 (m, 1H, CH), 1.87 (s, 3H, $COCH_3$), 1.75 – 1.33 (m, 12H, CH_2), 1.21 (dd, J = 18.8, 7.3 Hz, 3H, CH_3), 0.79 (dd, J = 11.5, 6.8 Hz, 6H, CH_3).

^{13}C NMR (101 MHz, DMSO-d_6) δ (ppm) = 207.16, 187.80, 169.69, 160.34, 156.70, 142.01, 128.27, 125.68, 71.09, 69.06, 57.12, 52.39, 44.45, 34.68, 28.29, 25.05, 22.46, 19.12, 17.87.

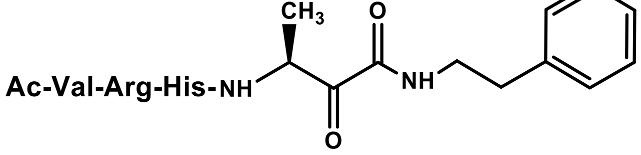

Compound ***12***

LC-MS analysis: One peak at 5.70 min, corresponding to $[M+H]^+$ 655.37 and $[M+2H]^{2+}$ 328.19.

HR-MS: Monoisotopic mass: 654.360179 calculated for $C_{31}H_{46}N_{10}O_6$, found $[M+H]^+$ 655.36774 and $[M+Na]^+$ 677.34979.

Yield: 1.5 mg (14 %).

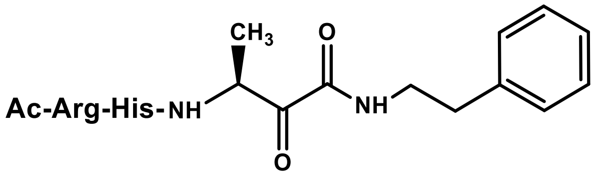

Compound ***13***

LC-MS analysis: One peak at 5.97 min, corresponding to $[M+H]^+$ 556.30 and $[M+2H]^{2+}$ 278.65.

HR-MS: Monoisotopic mass: 555.291765 calculated for $C_{26}H_{37}N_9O_5$, found $[M+H]^+$ 556.29939.

^{1}H NMR (401 MHz, DMSO-d_6) δ (ppm) = 14.14 (s, 2H, NH imidazole), 8.95 (s, 1H, aromatic), 8.77 (t, *J* = 4.7 Hz, 1H, COCONH), 8.38 – 8.08 (m, 3H, CONH), 7.54 (t, *J* = 5.3 Hz, 1H, aromatic), 7.41 – 7.25 (m, 4H, aromatic+NH_2), 7.25 – 7.16 (m, 3H, aromatic), 5.01 (p, *J* = 7.3 Hz, 1H, CH), 4.61 (t, *J* = 11.0 Hz, 1H, CH), 4.17 (dt, *J* = 13.4, 6.5 Hz, 1H, CH), 3.16 – 3.00 (m, 3H, CH_2 + CH), 2.92 (dt, *J* = 15.8, 8.3 Hz, 1H, CH), 2.77 (t, *J* = 7.7 Hz, 2H, CH_2), 1.86 (s, 3H, $COCH_3$), 1.67 – 1.53 (m, 1H, CH), 1.44 (tt, *J* = 12.7, 6.0 Hz, 3H, CH_2 + CH), 1.20 (dd, *J* = 10.6, 7.3 Hz, 3H, CH_3).

^{13}C NMR (101 MHz, DMSO-d_6) δ (ppm) = 213.71, 172.17, 166.88, 160.73, 157.11, 150.18, 134.19, 129.08, 128.83, 126.69, 52.99, 51.34, 49.87, 35.03, 29.04, 25.49, 22.87, 15.95.

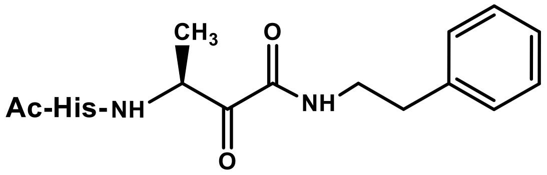

Compound ***14***

LC-MS analysis: One peak at 6.66 min, corresponding to $[M+H]^+$ 400.199 and $[M+H_2O+H]^+$ 418.21.

HR-MS: Monoisotopic mass: calculated 399.190654 for $C_{20}H_{25}N_5O_4$, found $[M+H]^+$ 400.19802 and $[M+Na]^+$ 422.17988.

^{1}H NMR (400 MHz, DMSO-d_6) δ (ppm) = 14.16 (s, 1H, imidazole), 8.96 (s, 1H, aromatic), 8.76 (dt, *J* = 11.7, 6.0 Hz, 1H, CONH), 8.49 – 8.38 (m, 1H, CONH), 8.21 – 8.11 (m, 1H, CONH), 7.38 – 7.25 (m, 3H, aromatic), 7.20 (d, *J* = 8.7 Hz, 3H, aromatic), 5.04 – 4.94 (m, 1H, CH), 4.70 – 4.60 (m, 1H, CH), 3.10 – 2.97 (m, 1H, CH_2), 2.93 – 2.81 (m, 1H, CH_2), 2.77 (t, *J* = 7.7 Hz, 2H, CH_2), 1.82 (d, *J* = 3.7 Hz, 3H, $COCH_3$), 1.19 (dd, *J* = 15.4, 7.3 Hz, 3H, CH_3).

^{13}C NMR (101 MHz, DMSO-d_6) δ (ppm) = 193.52, 169.85, 160.86, 129.07, 128.81, 126.66, 51.35, 49.85, 35.03, 22.99, 15.89.

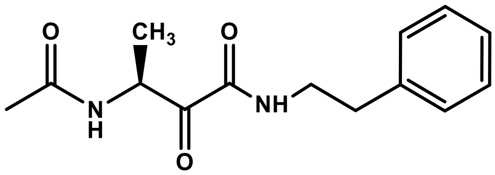

Compound ***15***

LC-MS analysis: One peak at 7.34 min, corresponding to the molecular mass: $[M+H]^+$ 263.14.

HR-MS: Monoisotopic mass: calculated 262.13176 for $C_{14}H_{18}N_2O_3$, found $[M+H]^+$ 236.13902 and $[M+Na]^+$ 285.12096.

^{1}H NMR (401 MHz, DMSO-d_6) δ (ppm) = 8.71 (t, *J* = 5.8 Hz, 1H, COCONH), 8.31 – 8.23 (m, 1H, CONH), 7.34 – 7.14 (m, 5H, aromatic), 4.98 – 4.89 (m, 1H, CH), 2.77 (t, *J* = 7.5 Hz, 3H, aliphatic), 1.82 (s, 3H, $COCH_3$), 1.18 (d, *J* = 7.3 Hz, 3H, CH_3).

^{13}C NMR (101 MHz, DMSO-d_6) δ (ppm) = 197.36, 169.06, 160.60, 139.04, 128.61, 126.17, 49.22, 34.57, 21.97, 15.57.

Supplementary Tables

Table S1, related to Fig. 1 and 7: Crystallographic data collection and refinement statistics

Ligand	Ac-VRHA-cmk	Ac-RVRHA-cmk	Compound ***9***	Compound ***10***
PDB code	5MT7	5MT8	5MT6	5MTF
**Data Collection**				
Cell dimensions				
a, *b*, *c* (Å)	110.9, 110.9, 126.3	110.2, 110.2, 128.9	111.7, 111.7, 124.2	111.3, 111.3, 124.5
γ (°)	120	120	120	120
Resolution (Å)	44.9-2.05 (2.11-2.05)*	44.9-1.95 (2.0-1.95)*	52.3-2.16 (2.23-2.16)*	44.9-1.78 (1.8-1.78)*
R_{merge}	0.036 (0.95)	0.032 (1.0)	0.08 (0.72)	0.045 (0.75)
$I / \sigma I$	28.0 (2.4)	24.5 (1.6)	10.2 (1.9)	18.64 (1.86)
Completeness (%)	99.7 (96.5)	99.6 (95.5)	99.4 (99.6)	99.8 (99.6)
CC1/2	0.99 (0.8)	0.99 (0.59)	0.99 (0.56)	1.0 (0.70)
Redundancy	9.9 (9.4)	6.5 (6.0)	4.7 (4.6)	5.1 (5.0)
Wilson B factor ($Å^2$)	53.6	48.2	43.9	37.9
**Refinement**				
Resolution (Å)	44.9-2.05	44.9-1.95	52.3-2.16	44.969-1.79
No. of reflections	18930	21617	16096	26791
R_{work} / R_{free}**	0.201/0.224	0.199/0.225	0.213/0.266	0.169/0.192
No. of atoms				
Total	1486	1531	1528	1670
Protein	1431	1426	1431	1480
Ligand***	37	42	58	60
Heteroatoms***	-	34	11	-
Water	18	29	28	68
B-factors ($Å^2$)				
Total	59.7	53.2	47.5	35.7
Protein	60.3	54.5	47.9	35.1
Ligand	66.3	57.4	53.8	38.1
Heteroatoms	-	71.5	68.5	39.9
Water	58.2	56.2	49.8	41.7
R.m.s.deviations				
Bond lengths (Å)	0.01	0.01	0.01	0.018
Bond angles (°)	1.4	1.3	1.5	1.65

* Values in parentheses are for highest-resolution shell.
** A subset of reflections (5 %) was used for calculation of R_{free} and remaining (95 %) reflections were used for calculation of R_{work}.
*** Heteroatoms denote detergent and ions. Ligand denotes the inhibitor covalently bonded to S201 and or H254.
